# One-shot K-region-selective annulative *π*-extension for nanographene synthesis and functionalization

**DOI:** 10.1038/ncomms7251

**Published:** 2015-02-16

**Authors:** Kyohei Ozaki, Katsuaki Kawasumi, Mari Shibata, Hideto Ito, Kenichiro Itami

**Affiliations:** 1 Institute of Transformative Bio-Molecules (WPI-ITbM) and Graduate School of Science, Nagoya University, Chikusa, Nagoya 464-8602, Japan; 2 JST-ERATO, Itami Molecular Nanocarbon Project, Nagoya University, Chikusa, Nagoya 464-8602, Japan

## Abstract

The optoelectronic nature of two-dimensional sheets of *sp*^2^-hydridized carbons (for example, graphenes and nanographenes) can be dramatically altered and tuned by altering the degree of *π*-extension, shape, width and edge topology. Among various approaches to synthesize nanographenes with atom-by-atom precision, one-shot annulative *π*-extension (APEX) reactions of polycyclic aromatic hydrocarbons hold significant potential not only to achieve a ‘growth from template’ synthesis of nanographenes, but also to fine-tune the properties of nanographenes. Here we describe one-shot APEX reactions that occur at the K-region (convex armchair edge) of polycyclic aromatic hydrocarbons by the Pd(CH_3_CN)_4_(SbF_6_)_2_/*o*-chloranil catalytic system with silicon-bridged aromatics as *π*-extending agents. Density functional theory calculations suggest that the complete K-region selectivity stems from the olefinic (decreased aromatic) character of the K-region. The protocol is applicable to multiple APEX and sequential APEX reactions, to construct various nanographene structures in a rapid and programmable manner.

## Abstract (web summary)

Bottom-up synthesis of nanographenes is highly desirable. Here, the authors report one-shot annulative *π*-extension reactions that occur at the convex armchair edge of polycyclic aromatic hydrocarbons, and show that unfunctionalized precursors can be used for *π*-component assembly and extension.

## Graphical abstract

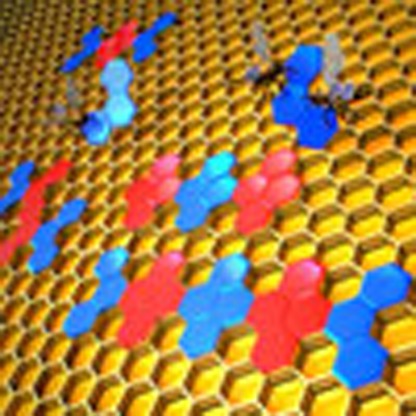

Nanographenes, which are nanometre-size subunits of graphenes (single-layer two-dimensional *sp*^2^-hybridized carbon sheets)^1^,^2^,^3^,^4^,^5^ with a tunable bandgap, have become hot molecular entities in the field of nanocarbon materials science^6^. As the properties of nanographenes depend heavily on the degree of *π*-extension, shape, width and edge topology, a novel bottom-up methodology for the precisely controlled synthesis of structurally uniform nanographenes is highly desirable^6^. Among various approaches to synthesize nanographenes with atom-by-atom precision^6^, one-shot annulative *π*-extension (APEX) reactions of polycyclic aromatic hydrocarbons (PAHs) hold significant potential not only to achieve a ‘growth from template’ synthesis of nanographenes, but also to fine-tune the properties of nanographenes.

In the last two decades, various bottom-up organic synthesis methods have been established for the controlled synthesis of large *π*-extended PAHs and nanographenes, as exemplified by the groundbreaking achievements of Müllen and colleagues^7^,^8^,^9^,^10^, Scott and colleagues^11^,^12^, Fasel and colleagues^13^,^14^ and others^15^ (Fig. 1a). In essence, most of the reported nanographene syntheses rely on a two-step sequence of (i) component assembly of small *π*-components, using reactions such as Diels–Alder reactions, Suzuki–Miyaura couplings and C–H activation reactions, to synthesize soluble nanographene precursors, followed by (ii) stitching (graphenization^6^) of soluble polyphenylene precursors by cyclodehydrogenation, flash-vacuum pyrolysis or photocyclization, to yield the target nanographenes (Fig. 1a)^6^,^16^. Although this state-of-the-art methodology has contributed significantly to the rapid progress of nanographene materials science, it has been well documented that the final and vital step of stitching (graphenization) is usually problematic^16^. For example, intramolecular oxidative cyclodehydrogenation by Lewis acids and oxidants (Scholl-type reactions) often suffer from problems such as incomplete stitching, lack of regioselectivity and undesirable rearrangements.

The continuing evolution of nanographene science is heavily dependent on the discovery of new reactions and strategies that allow rapid and predictable synthesis, and functionalization of nanographenes. Here we illustrate the significant potential of one-shot APEX reactions of template PAH molecules with *π*-extending agents (Fig. 1b) as an enabling concept that is complementary to the aforementioned two-step methodology. By devising APEX reactions that are selective to the specific regions of PAH structures (bay region, K-region and L-region, see Fig. 1b)^17^, the direction-controlled growth or polymerization of a template PAH producing structurally uniform nanographenes would become possible. These regions correspond to the concave armchair edge (bay region), convex armchair edge (K-region) and zigzag edge (L-region) in graphene nomenclature. APEX technology should also be useful for the late-stage fine-tuning of nanographene properties by subtly modifying their edge structures. Moreover, as a result of significant recent progress in surface-catalysed cyclodehydrogenation and subsequent epitaxial elongation reactions of PAHs by the group of Fasel and colleagues^18^, a number of *π*-extended PAHs provided by the APEX technology could also be converted into structurally uniform carbon nanotubes^18^,^19^.

Although the full potential of APEX reactions in nanographene chemistry is yet to be realized partly because the concept had not been formulated, significant experimental^20^,^21^,^22^,^23^,^24^,^25^ and theoretical^26^ advances have appeared, describing methods for bay-region-specific *π*-extension in PAHs. For example, Scott has revisited the finding of the Diels–Alder reactivity at the PAH bay regions by Clar *et al*.^27^ and proposed its use for metal-free growth of single-chirality carbon nanotubes^20^,^21^,^22^,^23^,^24^,^25^,^26^ (Fig. 1c). This represents a bay-region-selective APEX reaction in our definition. However, no APEX reactions at other PAH regions (L-region and K-region) have been developed up until now. Although the dimerization of perylene under Scholl-type coupling to furnish quaterrylene represents the closest example of an L-region-selective APEX reaction, this can be categorized as the two-step method having a serious problem at the stitching (cyclodehydrogenation) stage^28^. The reactions at K-regions (convex armchair edges) are considered to be particularly difficult, as K-region bonds tend to have relatively high bond orders, and most aromatic substitution reactions occur preferentially at other aromatic C–H bonds^17^.

Our APEX campaign^29^,^30^,^31^,^32^,^33^,^34^ began when we serendipitously discovered Pd(OAc)_2_/*o*-chloranil as the first-generation C–H activation catalyst for PAHs in 2011 (ref. 35). This catalyst uniquely and effectively promotes the C–H arylation of non-functionalized PAHs with arylboroxines, with complete K-region selectivity. When coupled with the Scholl-type cyclodehydrogenation of the thus-formed arylated PAHs, a number of structurally intriguing nanographenes such as warped nanographenes^34^ (Fig. 1a) have been synthesized. Although this was a two-step nanographene synthesis at the time, we felt that the observed C–H activation reactivity and selectivity might be translated into an APEX reaction when the K-region is activated and annulated with properly arranged 1,4-dimetal *π*-units in a [2+4] annulation manner. Here, in we describe a one-shot APEX reaction that occurs selectively at the K-region of PAHs via double C–H activation^36^,^37^,^38^,^39^ (Fig. 1d). This protocol is applicable to multiple APEX and sequential APEX reactions to construct various nanographene structures in a rapid and programmable manner.

## Results

### Development of catalyst

**Figure 2:**
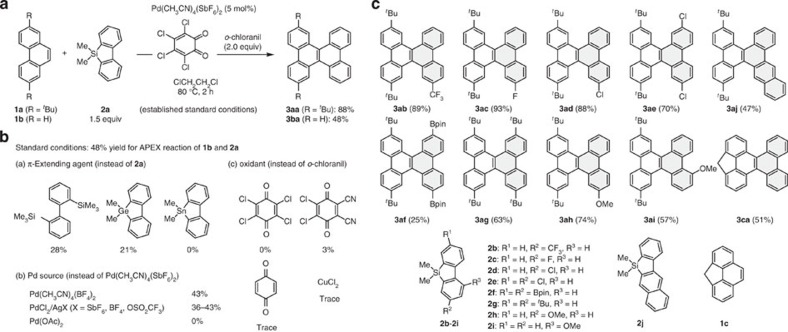
One-shot K-region-selective C–H activation APEX. (**a**) The established reaction conditions. (**b**) Deviation from standard conditions (effect of reaction parameters) in the reaction of **1b** and **2a**. (**c**) Scope of one-shot, K-region-selective, C–H activation APEX. Reaction conditions: polycyclic aromatic compounds **1** (0.2 mmol), silicon-bridged aromatics **2** (1.5 equiv), Pd(CH_3_CN)_4_(SbF_6_)_2_ (5 mol%), *o*-chloranil (2.0 equiv), 1,2-dichloroethane, 80 °C, 2 h. Bpin, pinacolatoboryl.

We began our study by examining various palladium salts, ligands, oxidants and *π*-extending agents for the APEX reaction of phenanthrenes **1a** and **1b**. After extensive screening, we determined that Pd(CH_3_CN)_4_(SbF_6_)_2_ and *o*-chloranil serve as an efficient pre-catalyst and oxidant respectively, while silicon-bridged aromatics **2** are optimal π-extending agents (Fig. 2a). For example, when 2,7-di-*tert*-butyl-phenanthrene (**1a**: 1.0 equiv) was treated with dimethyldibenzosilole^40^ (**2a**: 1.5 equiv) in 1,2-dichloroethane at 80 °C for 2 h in the presence of Pd(CH_3_CN)_4_(SbF_6_)_2_ (5 mol%) and *o*-chloranil (2.0 equiv), the corresponding K-region-annulated APEX product **3aa** was obtained in 88% isolated yield, representing our standard APEX conditions. Unfunctionalized phenanthrene (**1b**) also underwent an APEX reaction with **2a** to yield dibenzo[*g,p*]chrysene (**3ba**). It should be noted that under these conditions, we observed the annulation exclusively at the K-region of phenanthrene **1a** or **1b**.

Listed in Fig. 2b are the effects of variations from the standard APEX conditions (**1b**+**2a**→**3ba**: 48% yield). For full lists of the effects of reaction parameters, see Supplementary Tables 2–4. As for the *π*-extension agents, we found that other dimetallobiphenyl derivatives such as 2,2′-bis(trimethylsilyl)-1,1′-biphenyl and dimethyldibenzogermole also reacted but led to the desired product in much lower yield. Dimethyldibenzostannole did not yield the product but generated a considerable amount of tetraphenylene and quaterphenyl by homodimerization. Changing the methyl groups of **2a** only had detrimental effect on reaction efficiency. The commercially available cationic palladium complex Pd(CH_3_CN)_4_(BF_4_)_2_ showed APEX activity comparable to Pd(CH_3_CN)_4_(SbF_6_)_2_. Similar catalytic activities were also observed with the combined use of PdCl_2_ and silver salts such as AgOSO_2_CF_3_, AgBF_4_ and AgSbF_6_. On the other hand, neutral Pd(OAc)_2_ (our previous palladium pre-catalyst)^29^ did not promote the APEX reaction at all. These results clearly indicate the importance of a cationic palladium species for the APEX reaction to occur. In the investigation of oxidants, commonly used oxidants such as benzoquinone, *p*-chloranil, DDQ and CuCl_2_ (ref. 35) displayed virtually no APEX-type activity. We assume that the high reactivity of *o*-chloranil stems not only from its high oxidation aptitude but also from its unique *o*-quinone structure, which can bind to palladium in a bidentate manner and modulate the redox property effectively^41^,^42^. Our preferred solvent is 1,2-dichloroethane, but aromatic solvents such as toluene, chlorobenzene, 1,2-dichlorobenzene, fluorobenzene and trifluoromethylbenzene can also be used for the present APEX reactions.

### Scope of K-region-selective APEX

As shown in Fig. 2c, various structurally and electronically diverse silicon-bridged aromatics **2** were found to react with 2,7-di-*tert*-butylphenanthrene (**1a**), providing the corresponding dibenzo[*g,p*]chrysenes (**3ab**–**3aj**) in good-to-excellent yield with virtually complete K-region selectivity. In particular, dibenzosiloles having electron-deficient substituents (**2b**–**2e**)^40^,^43^ showed excellent reactivity. The tribenzo[*a*,*c*,*f*]tetraphene framework (**3aj**) can be readily constructed by the APEX reaction of **1a** and benzonaphthosilole **2j**^43^. Notably, dichloro- and diboryl-substituted dibenzosiloles underwent the APEX reaction smoothly, leaving C–Cl and C–B bonds intact (**3ad**, **3ae** and **3af**). The tolerance of the reaction for these bonds makes it attractive for further *π*-extension and functionalization, using well-established cross-coupling chemistry. Furthermore, methylene-bridged phenanthrene **1c** reacted with **2a** to afford benzoindenochrysene **3ca**, which can potentially lead to soluble nanographenes by facile substitution at the methylene moiety. Ease of post-functionalization is particularly advantageous for controlled surface alignment of nanographenes for device applications.

### Mechanistic considerations of K-region-selective APEX

**Figure 3:**
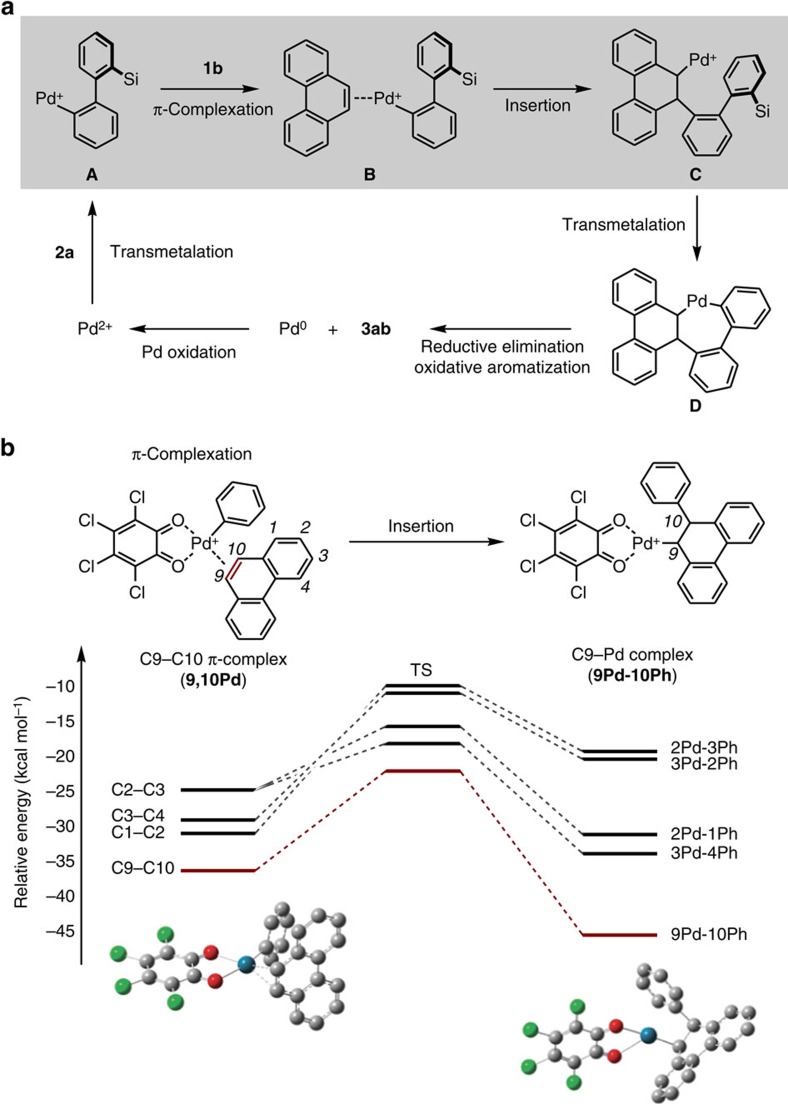
Mechanistic considerations. (**a**) A possible mechanism of K-region-selective C–H activation APEX. (**b**) Theoretical calculations of π-complexation and insertion steps. Structures were optimized by the density functional theory (DFT^44^) calculations using B3PW91 hybrid functional^45^,^46^ (hydrogen atoms are omitted for clarity). Reaction pathways were followed by intrinsic reaction coordinate (IRC^47^,^48^) computations, and high-accuracy, single-point energy calculations of DFT-optimized structures were performed with Møller–Plesset perturbation theory (MP2) (ref. 49). Energies are relative to that of *o*-chloranil-bound cationic phenylpalladium species.

Although the exact mechanism of the present APEX reaction remains unclear, our current assumption is shown in Fig. 3a. A palladium(II) species undergoes the first transmetalation with silicon-bridged aromatic **2a**, followed by coordination of phenanthrene (**1b**) to arylpalladium intermediate **A** at the K-region, yielding *π*-complex **B**. Coordination-induced insertion at the K-region C=C bond, followed by a second transmetalation forms palladacycle intermediate **D**. Reductive elimination and oxidative aromatization yields APEX product **3ab** and palladium(0), the latter of which is oxidized to the active palladium(II) species by the action of *o*-chloranil.

We assume that the K-region selectivity in the present APEX reaction stems from the preferential palladium *π*-complexation at the K-region of PAHs before the insertion step, as depicted in Fig. 3a. To prove this hypothesis, we conducted density functional theory^44^ calculations (using B3PW91 hybrid functional^45^,^46^) for the *π*-complexation and insertion steps on a model reaction of *o*-chloranil-bound cationic phenylpalladium species with phenanthrene yielding the alkylpalladium species shown in Fig. 3b (a model reaction relevant to **A**→**B**→**C** in Fig. 3a). Reaction pathways were followed by intrinsic reaction coordinate^47^,^48^ computations, and high-accuracy, single-point energy calculations of density functional theory-optimized structures were performed with Møller–Plesset perturbation theory^49^. Among possible *π*-coordination complexes at C1–C2, C2–C3, C3–C4 and C9–C10 bonds, the *π*-complex at C9–C10 (K-region) was found to be most stable (**9**,**10Pd**). This may be due to the tendency of this bond to have the most olefinic (less aromatic) character^17^,^50^. We also calculated all possible transition states of insertion from these *π*-complexes to give alkylpalladium complexes (Fig. 3b). The formation of C9–Pd complex (**9Pd–10Ph**), which leads to APEX at the K-region, was found to be most favourable both kinetically and thermodynamically. Thus, the basis of K-region selectivity in our new APEX reaction has been supported by computational theory. Detailed computational studies of Pd/*o*-chloranil-catalysed C–H activation, including the present APEX reactions, will be reported in due course. Based on these calculations, we predict the most olefinic (least aromatic) K-region *π*-bond to be the first APEX reaction site in future functionalizations of related *π*-extended PAHs and nanographenes.

### Multiple APEX and sequential APEX

**Figure 4:**
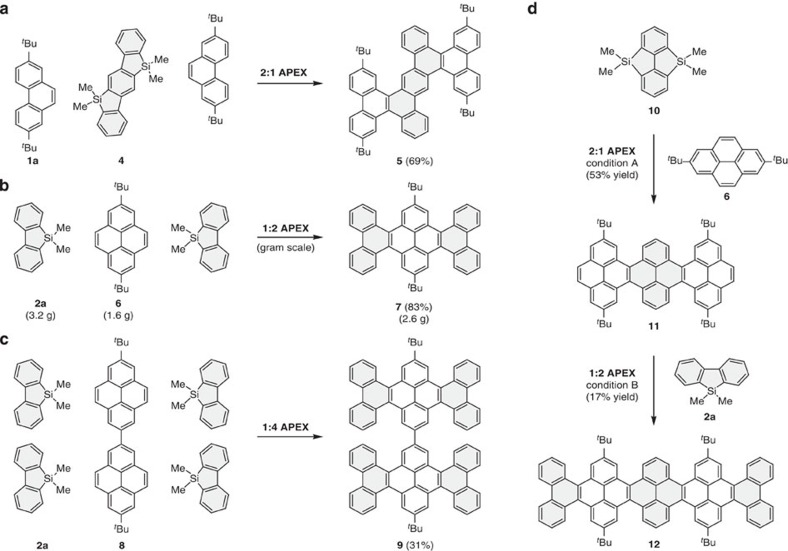
Multiple APEX and sequential APEX. Multiple APEX (**a**–**c**). (**a**) 2:1 APEX. Reaction conditions: **4** (1 equiv), **1a** (5 equiv), Pd(CH_3_CN)_4_(SbF_6_)_2_ (5 mol%), *o*-chloranil (4 equiv), 1,2-dichloroethane, 80 °C, 2 h. (**b**) 1:2 APEX. Reaction conditions: **6** (1 equiv), **2a** (3 equiv), Pd(CH_3_CN)_4_(SbF_6_)_2_ (5 mol%), *o*-chloranil (4 equiv), 1,2-dichloroethane, 80 °C, 8 h. (**c**) Reaction conditions: **8** (1 equiv), **2a** (10 equiv), Pd(CH_3_CN)_4_(SbF_6_)_2_ (15 mol%), *o*-chloranil (10 equiv), 1,2-dichloroethane, 80 °C, 1 h. (**d**) Sequential APEX. 2:1 APEX conditions (A): **6** (3 equiv), **10** (1 equiv), Pd(CH_3_CN)_4_(SbF_6_)_2_ (5 mol%), *o*-chloranil (4 equiv), 1,2-dichloroethane, 80 °C, 6 h. 1:2 APEX conditions (B): **11** (1 equiv), **2a** (4 equiv), Pd(CH_3_CN)_4_(SbF_6_)_2_ (15 mol%), *o*-chloranil (5 equiv), 1,2-dichloroethane, 80 °C, 4 h.

To showcase the utility of our APEX methodology in accessing a variety of nanographene (*π*-extended PAH) structures in a rapid and programmable manner, we examined several types of multiple APEX reactions (Fig. 4a–c). For example, a 2:1 APEX reaction occurs when treating ladder-type *bis*-silicon-bridged *p*-terphenyl **4** (ref. 51) with an excess of 2,7-di-*tert*-butylphenanthrene (**1a**) in the presence of Pd(CH_3_CN)_4_(SbF_6_)_2_/*o*-chloranil, to construct the dibenzodiphenanthroanthracene framework **5** in 69% yield (Fig. 4a). Other isomers were not observed in the reaction, highlighting the fidelity of the present method to specific reaction sites. An alternative mode of the double APEX reaction (1:2 APEX) was also possible by the reaction of 2,7-di-*tert*-butylpyrene **6** (1.6 g, 1.0 equiv) and dibenzosilole **2a** (3.2 g, 3.0 equiv) in the presence of Pd(CH_3_CN)_4_(SbF_6_)_2_ (5 mol%) and *o*-chloranil (4.0 equiv) (Fig. 4b). It is noteworthy that the reaction could be conducted on a gram scale to yield di-*tert*-butylhexabenzotetracene **7** in 2.6 g (83% yield). The gram-scale synthesis clearly underscores the high capability of the present reaction conditions for multiple APEX reactions. Furthermore, the Pd(CH_3_CN)_4_(SbF_6_)_2_/*o*-chloranil system effectively promoted the one-shot fourfold APEX reaction of 7,7′-di-*tert*-butyl-2,2′-bipyrene **8** (1.0 equiv) with dibenzosilole **2a** (10 equiv), to provide bihexabenzotetracene **9** in 31% yield (Fig. 4c).

To further examine the applicability of APEX technology for the construction of larger molecules, sequential APEX reactions were investigated (Fig. 4d). Pleasingly, the 2:1 APEX reaction of 2,7-di-*tert*-butylpyrene **6** (3.0 equiv) with *bis*-silicon bridged biphenyl **10** (ref. 51) (1.0 equiv) under Pd(CH_3_CN)_4_(SbF_6_)_2_/*o*-chloranil conditions afforded tetra-*tert*-butylhexabenzohexacene **11** in 53% yield. The follow-up 1:2 APEX reaction of **11** (1.0 equiv) with dibenzosilole **2a** (4.0 equiv) also took place to furnish the target decabenzooctacene framework **12** in 17% yield. In this particular reaction, we recovered a considerable amount of starting material likely to be attributed to poor solubility in the reaction media. Despite being relatively small in molecular size, the electronic structures of these nanographenes can be systematically altered. With the increase in molecular length, the HOMO-LUMO gap becomes smaller. In line with this trend, we observed decent red shift in both absorption and fluorescence (see Supplementary Fig. 41 for details).

## Discussion

It should also be mentioned that we observed the first sign of the possibility of employing APEX in an oligomerization/polymerization manifold when we detected oligomers by matrix-assisted laser desorption/ionization time-of-flight mass spectrometry analysis in the first 2:1 APEX reaction shown in Fig. 4d. Although we have not yet investigated this possible mode of reaction extensively, we envisage that the present APEX reaction could be applied to even larger molecules through judicious choice of solubilizing substituents on the substrates. Thus, the present result bodes well for the potential application of our APEX methodology to the bottom-up synthesis of graphene nanoribbons with controlled edge structures.

The present APEX methodology is complementary to the state-of-the-art two-step nanographene synthetic methods, thereby finding significant use in the ‘growth from template’ nanographene synthesis and in the late-stage fine-tuning of nanographene properties. Moreover, our APEX technology is not limited to the synthesis and functionalization of *π*-extended PAHs and nanographenes. One of the most significant features of the present APEX reaction is that unfunctionalized PAHs can be directly used for *π*-component assembly and *π*-extension without any pre-functionalization. Thus, various *π*-conjugated molecules made by many research groups (for many different purposes) will be suitable substrates for our APEX reaction, furnishing even more exciting classes of *π*-conjugated systems. The realization of APEX polymerization, the development of new APEX reactions for other PAH regions and acquisition of the first structure–property relationships for various nanographene structures are now ongoing in our laboratory.

## Methods

### Materials and characterization data

For the synthesis of all starting materials, siloles and *π*-extended PAHs, and their characterization, see Supplementary Methods. ^1^H, ^13^C and ^19^F NMR spectra were obtained for all compounds, see Supplementary Fig. 1–40. Ultraviolet–visible absorption and fluorescence spectra of **6**, **11** and **12** are provided in Supplementary Fig. 41. Calculated energy surface of *π*-complexation and insertion are provided in Supplementary Fig. 42. Calculated energies of stationary points and their Cartesian coordinates are summarized in Supplementary Table 1. Effects of reaction parameters are provided in Supplementary Tables 2–4.

### Typical procedure of K-region-selective APEX reaction

2,7-Di-*tert*-butyl-phenanthrene (**1a**: 58 mg, 0.2 mmol, 1.0 equiv), dimethyldibenzosilole (**2a**: 63 mg, 0.3 mmol, 1.5 equiv), Pd(CH_3_CN)_4_(SbF_6_)_2_ (7.4 mg, 10 μmol, 5 mol%), *o*-chloranil (98 mg, 0.4 mmol, 2.0 equiv) and a stirring bar were placed in a screw cap test tube. The tube was sealed with a perforated plastic cap and air was removed though a needle under reduced pressure. After back filling with nitrogen or argon, 1,2-dichloroethane (2 ml) was added and the needle was removed to seal up the test tube. The tube was fixed in an aluminum heating block and the mixture was stirred at 80 °C. After 2 h, the reaction mixture was cooled to room temperature and then passed through a short pad of silica gel (eluent: CH_2_Cl_2_). After the organic solvents were removed under reduced pressure, the residue was purified by silica-gel chromatography (eluent: hexane) to afford 3,14-di-*tert*-butyldibenzo[*g*,*p*]chrysene (**3aa**) in 88% yield (77.0 mg) as a white powder.

## Author contributions

K.I. conceived the concept. K.O., K.K. and H.I. conducted experiments. M.S. performed theoretical calculations. K.I and H.I. prepared the manuscript, with feedback from other authors.

## Additional information

**How to cite this article:** Ozaki, K. *et al*. One-shot K-region-selective annulative *π*-extension for nanographene synthesis and functionalization. *Nat. Commun.* 6:6251 doi: 10.1038/ncomms7251 (2015).

## Supplementary Material

Supplementary Information Supplementary Figures 1-42, Supplementary Tables 1-4, Supplementary Methods and Supplementary References

## Figures and Tables

**Figure 1:**
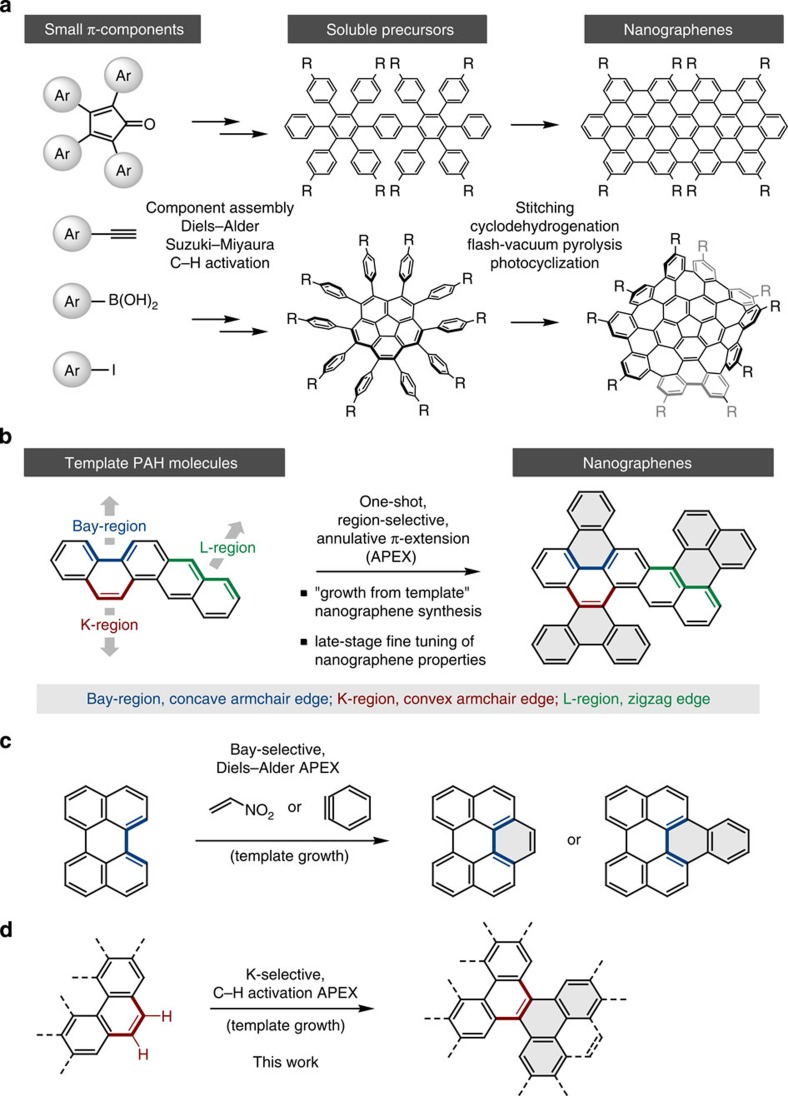
Organic synthesis approaches for structurally uniform nanographenes. (**a**) The well-established two-step synthesis of nanographenes through π-component-assembling reaction and stitching (graphenization). (**b**) One-shot, region-selective APEX as alternative synthesis and functionalization of nanographenes through ‘growth from template’. (**c**) One-shot, bay-region-selective APEX by Diels–Alder reaction. (**d**) One-shot, K-region-selective APEX by double C–H activation (this work).
