# Supplementary material for: One-shot K-region-selective annulative π-extension for nanographene synthesis and functionalization
Source: Nat Commun. 2015 Feb 16;6:6251. doi: 10.1038/ncomms7251 (PMC4339889; doi:10.1038/ncomms7251)
Supplement: Supplementary Information — Supplementary Figures 1-42, Supplementary Tables 1-4, Supplementary Methods and Supplementary References [file ncomms7251-s1.pdf]

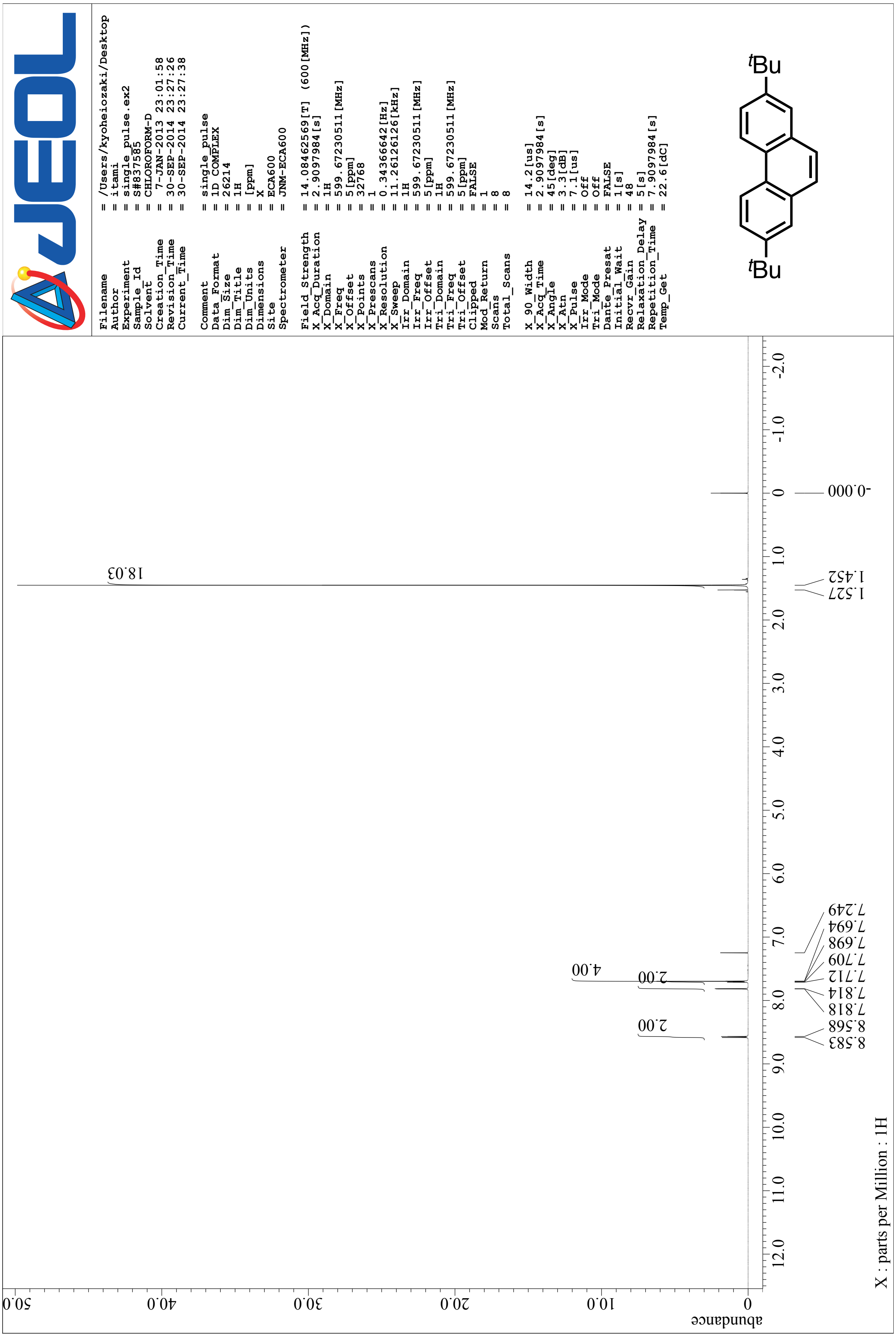

Supplementary Figure 1. ^{1}**H NMR (600 MHz,** $CDCl_3$**) of 1a**

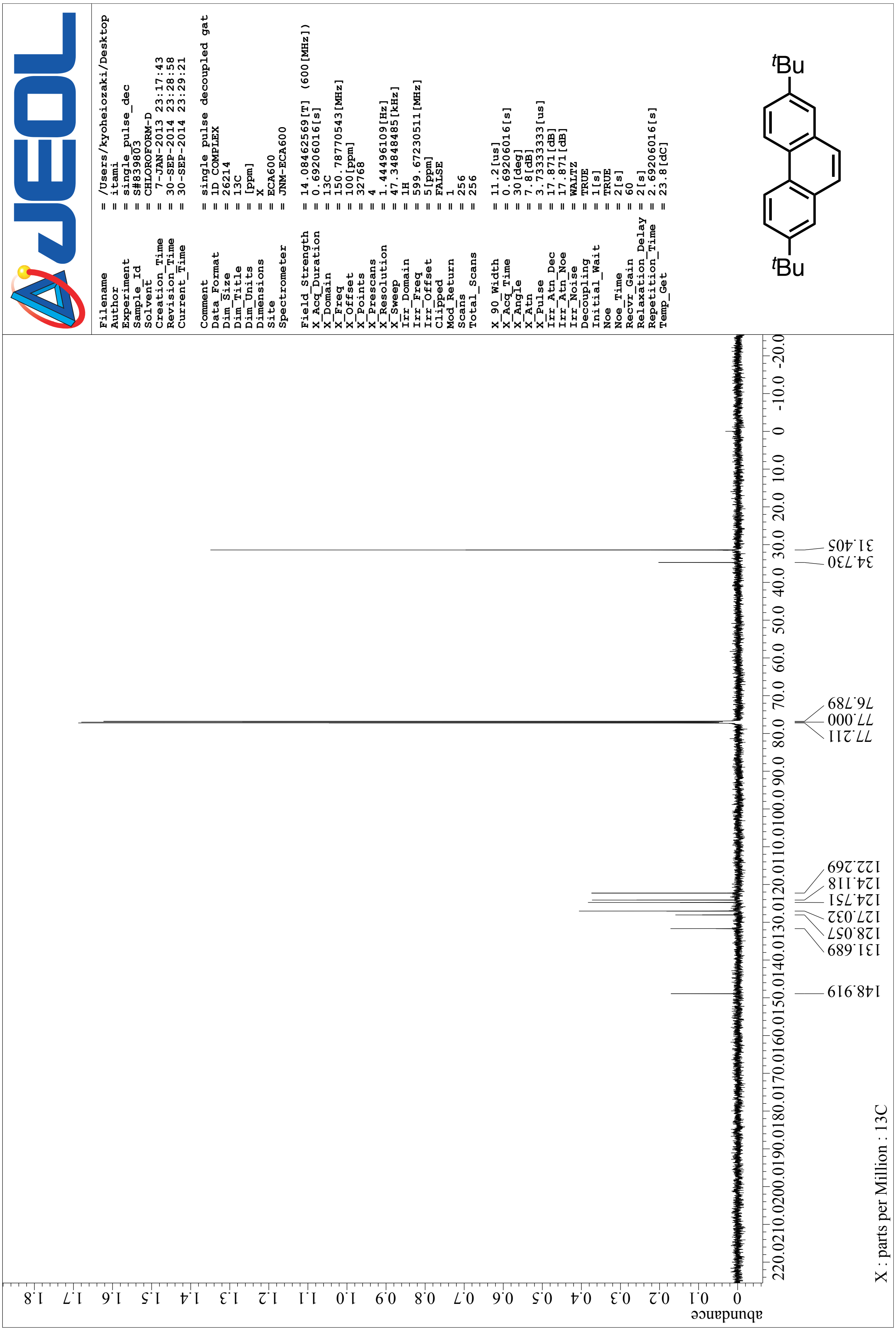

Supplementary Figure 2. ^{13}**C NMR (150 MHz, CDCl$_3$) of 1a**

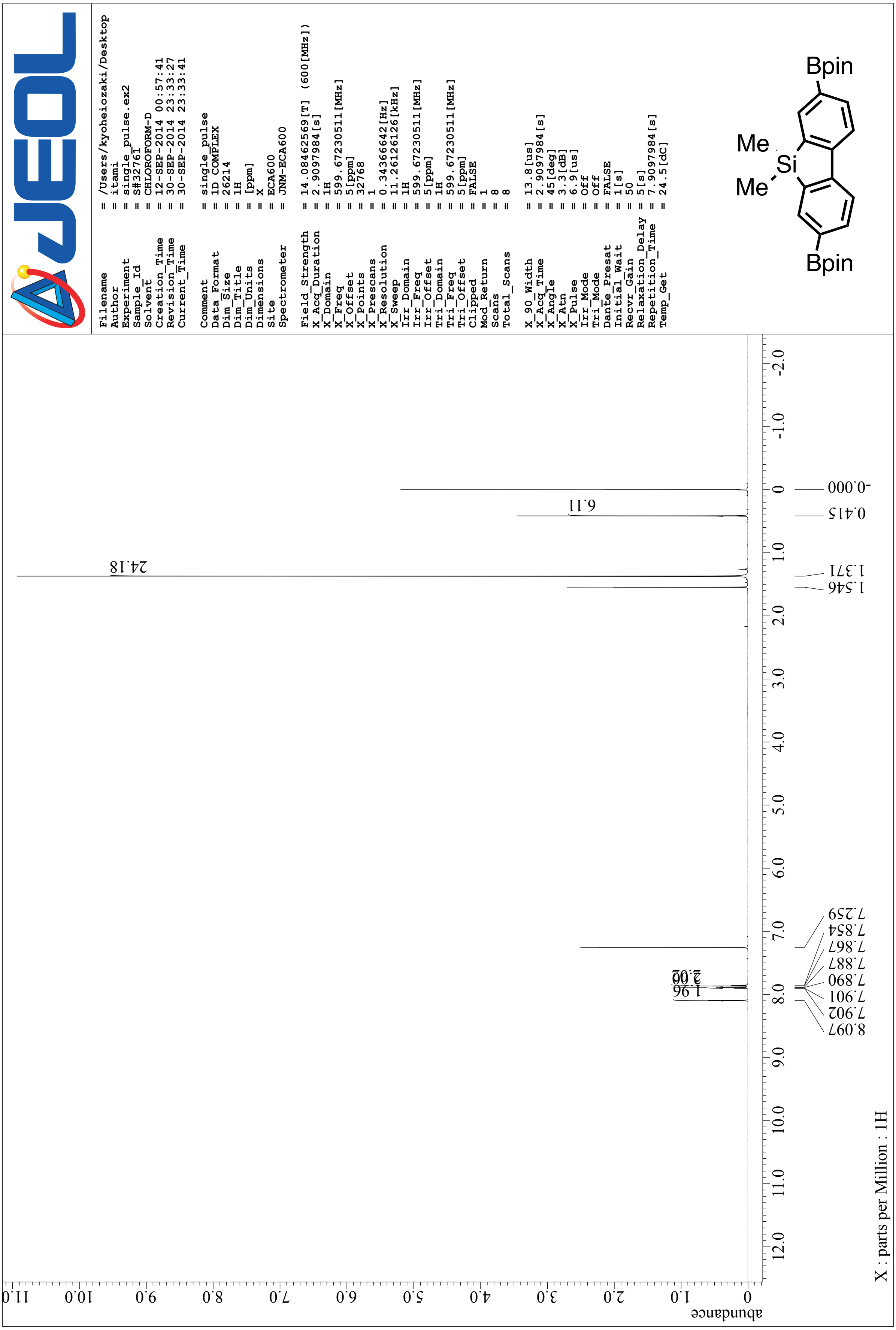

Supplementary Figure 3. ^{1}H NMR (600 MHz, $CDCl_3$) of **2f**

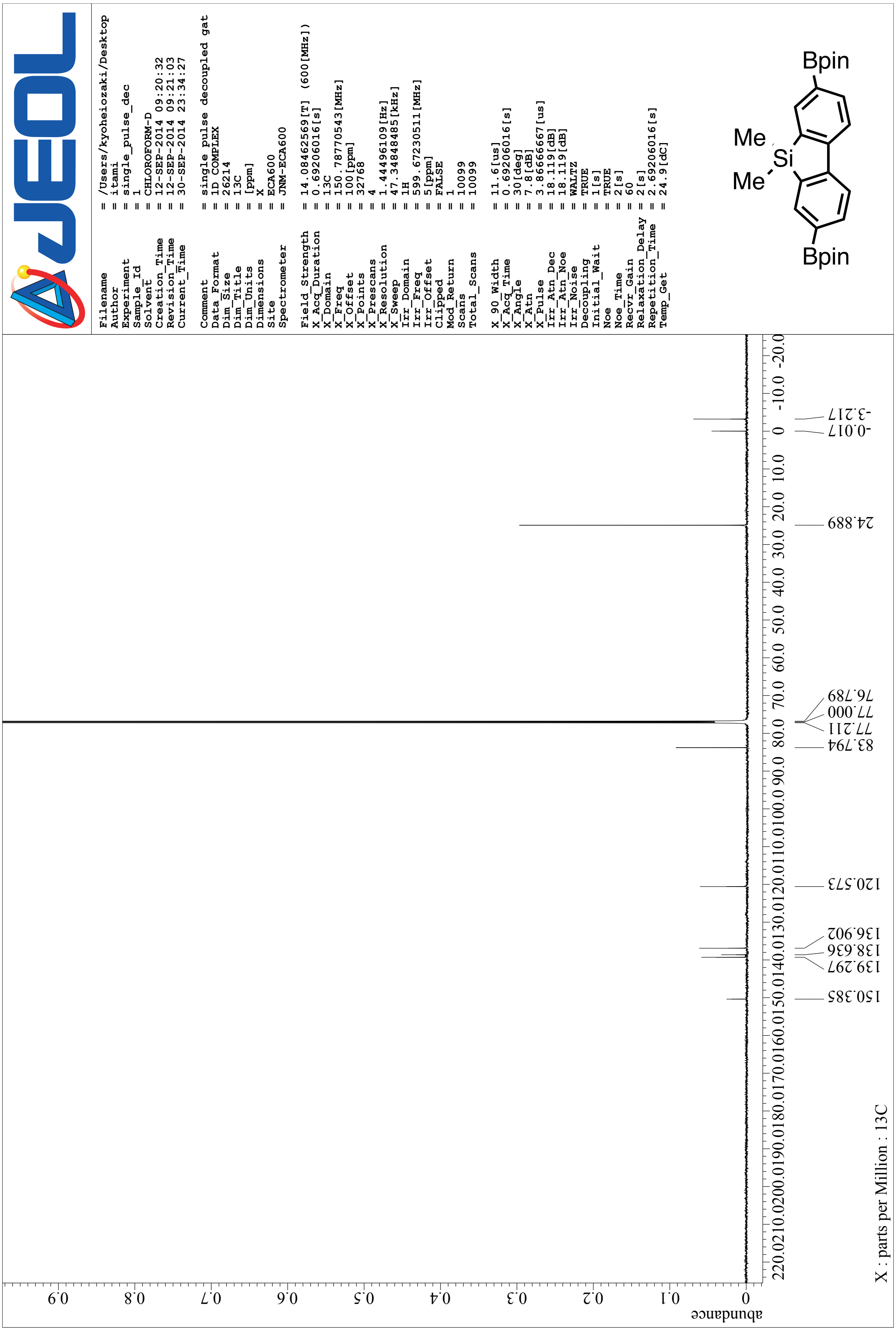

Supplementary Figure 4. ^{13}**C NMR (150 MHz, CDCl$_3$) of 2f**

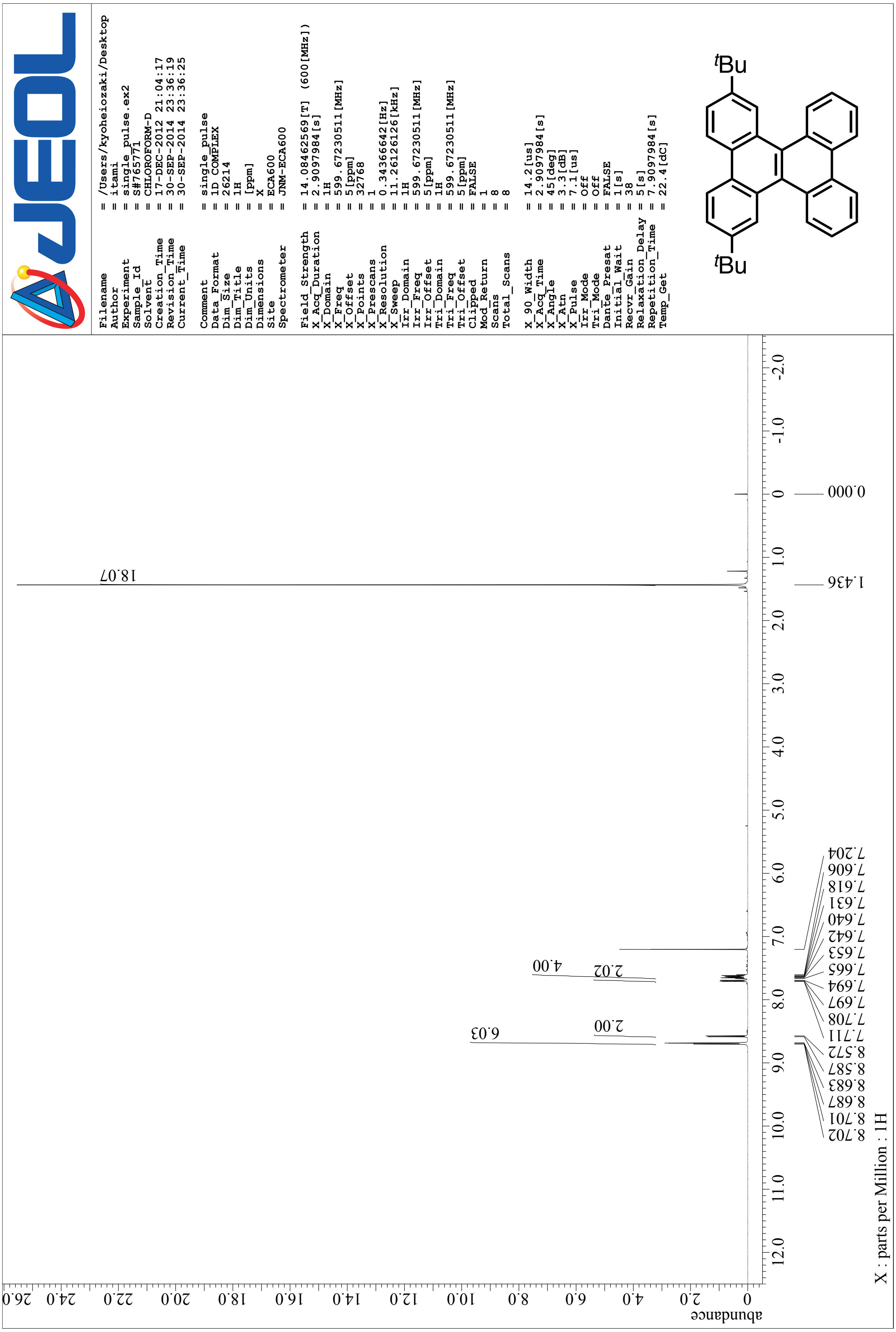

Supplementary Figure 5. ^{1}H NMR (600 MHz, $CDCl_3$) of 3aa

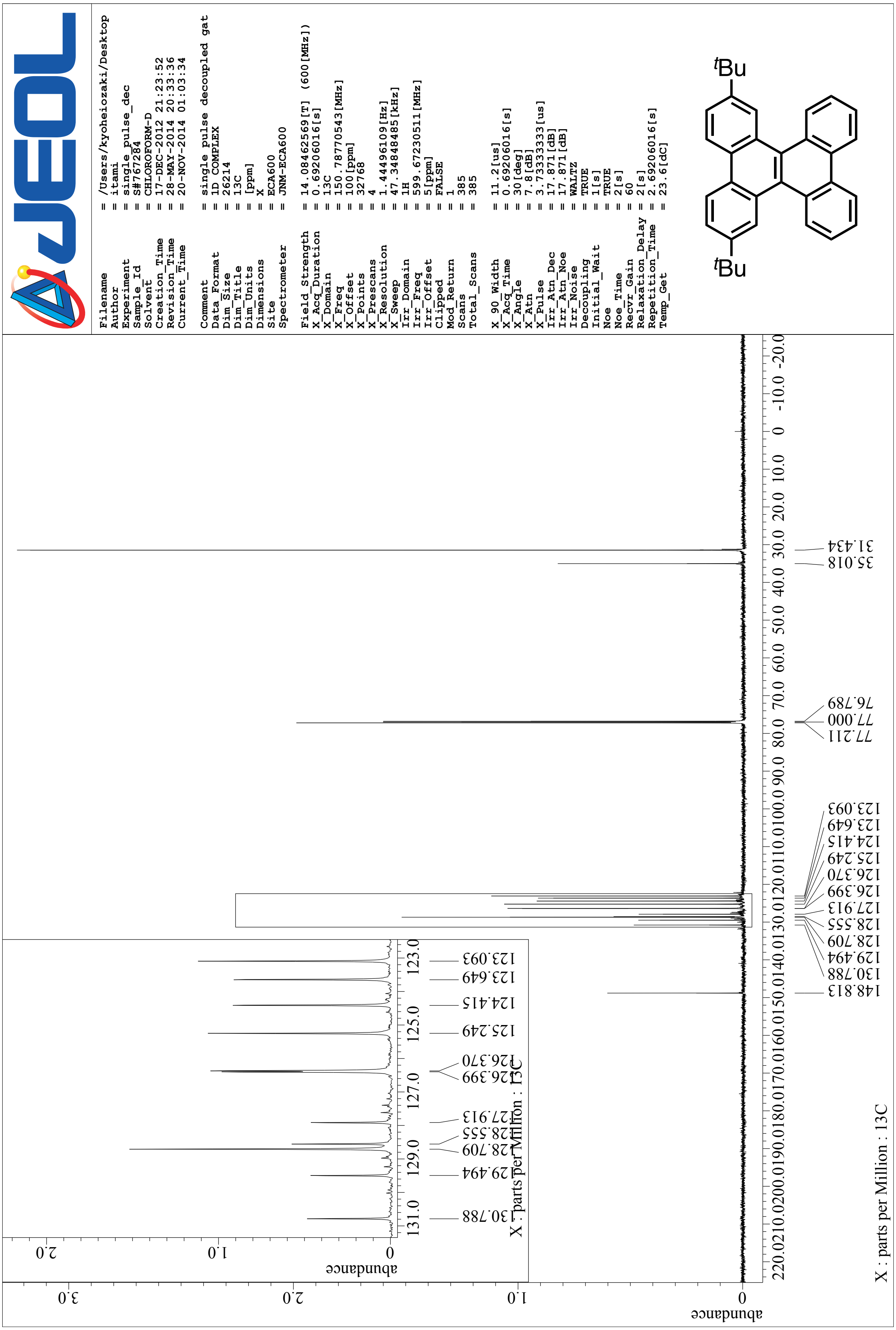

Supplementary Figure 6. ^{13}**C NMR (150 MHz,** $CDCl_3$**) of 3aa**

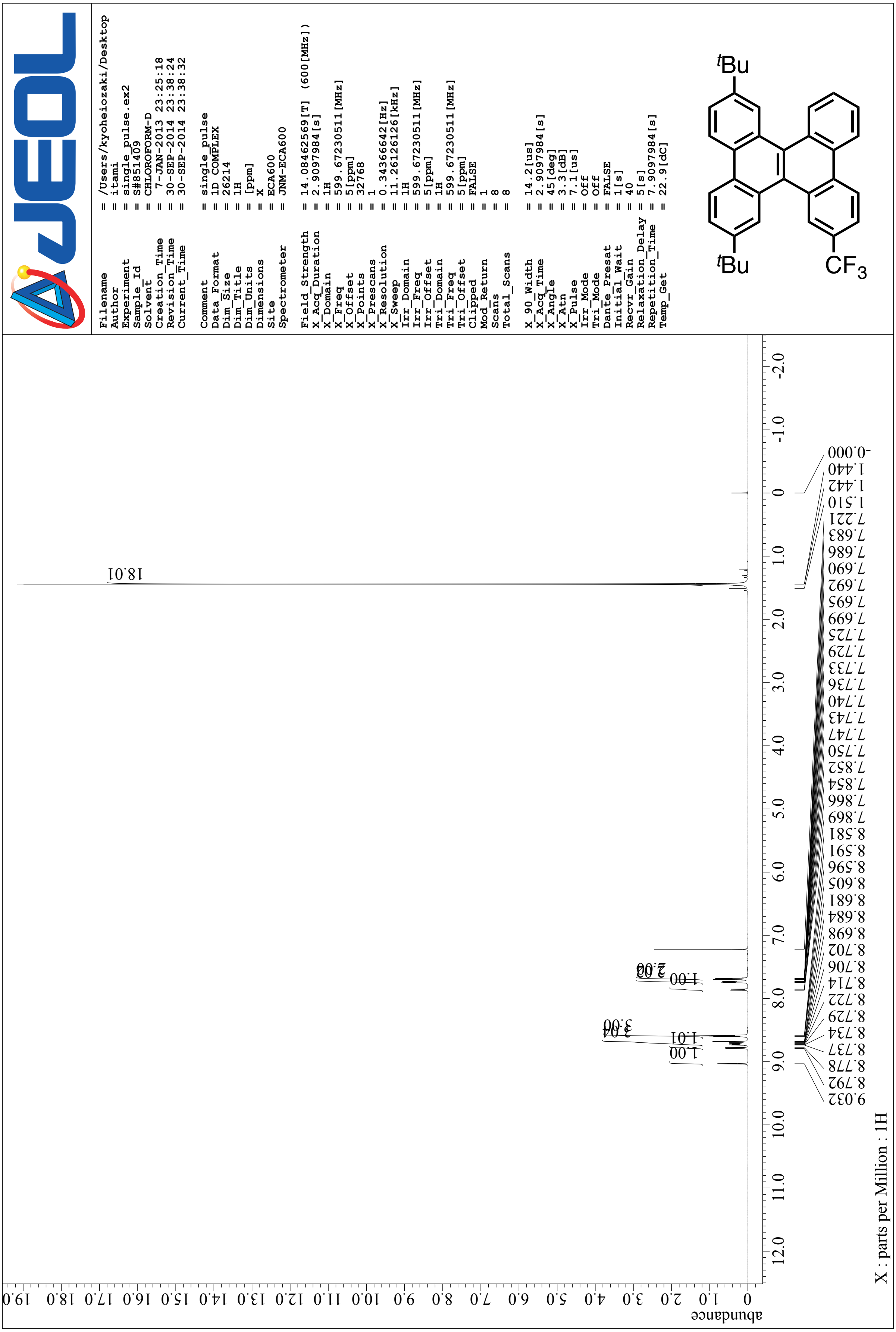

Supplementary Figure 7. ^{1}H NMR (600 MHz, $CDCl_3$) of 3ab

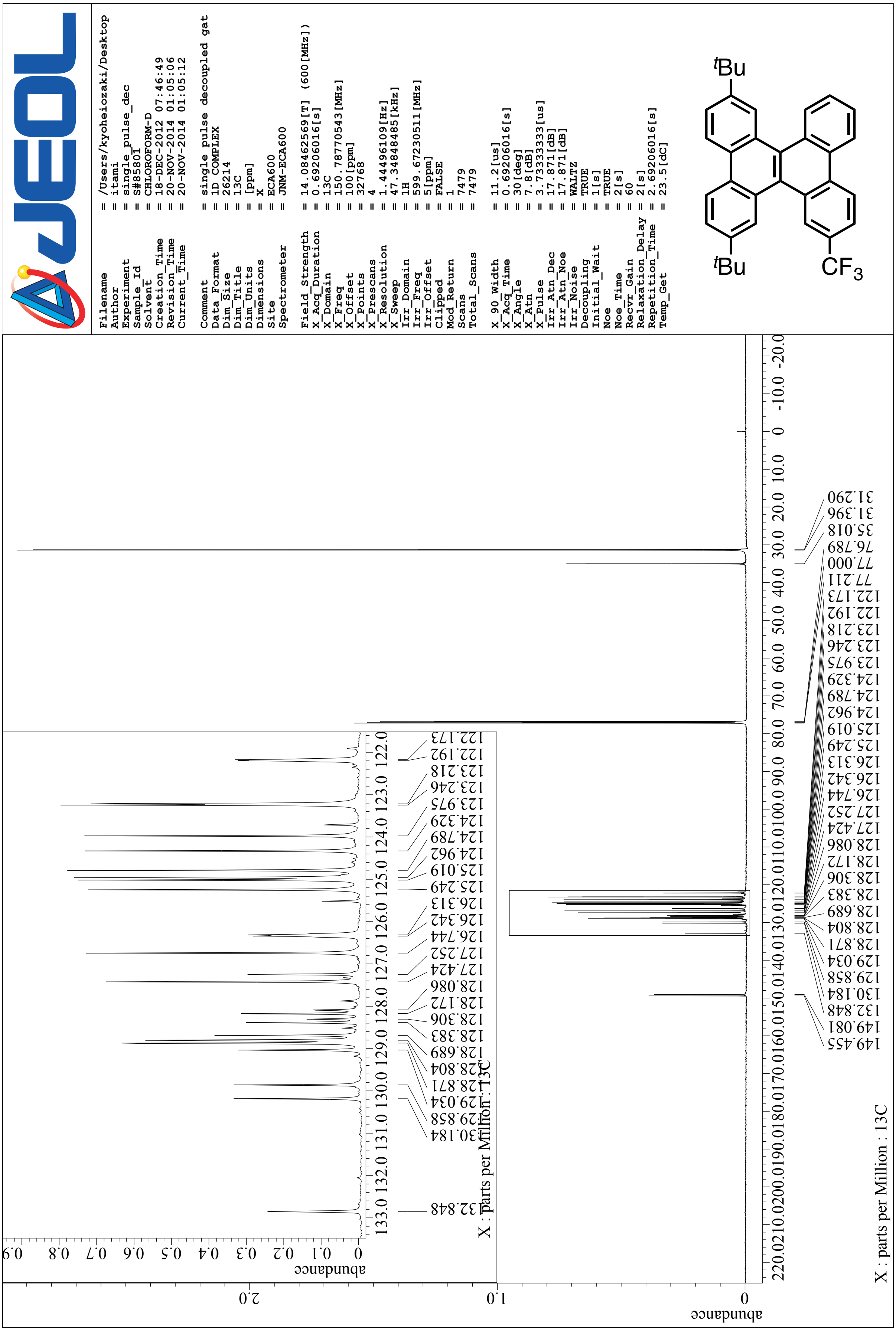

Supplementary Figure 8. ^{13}**C NMR (150 MHz, CDCl$_3$) of 3ab**

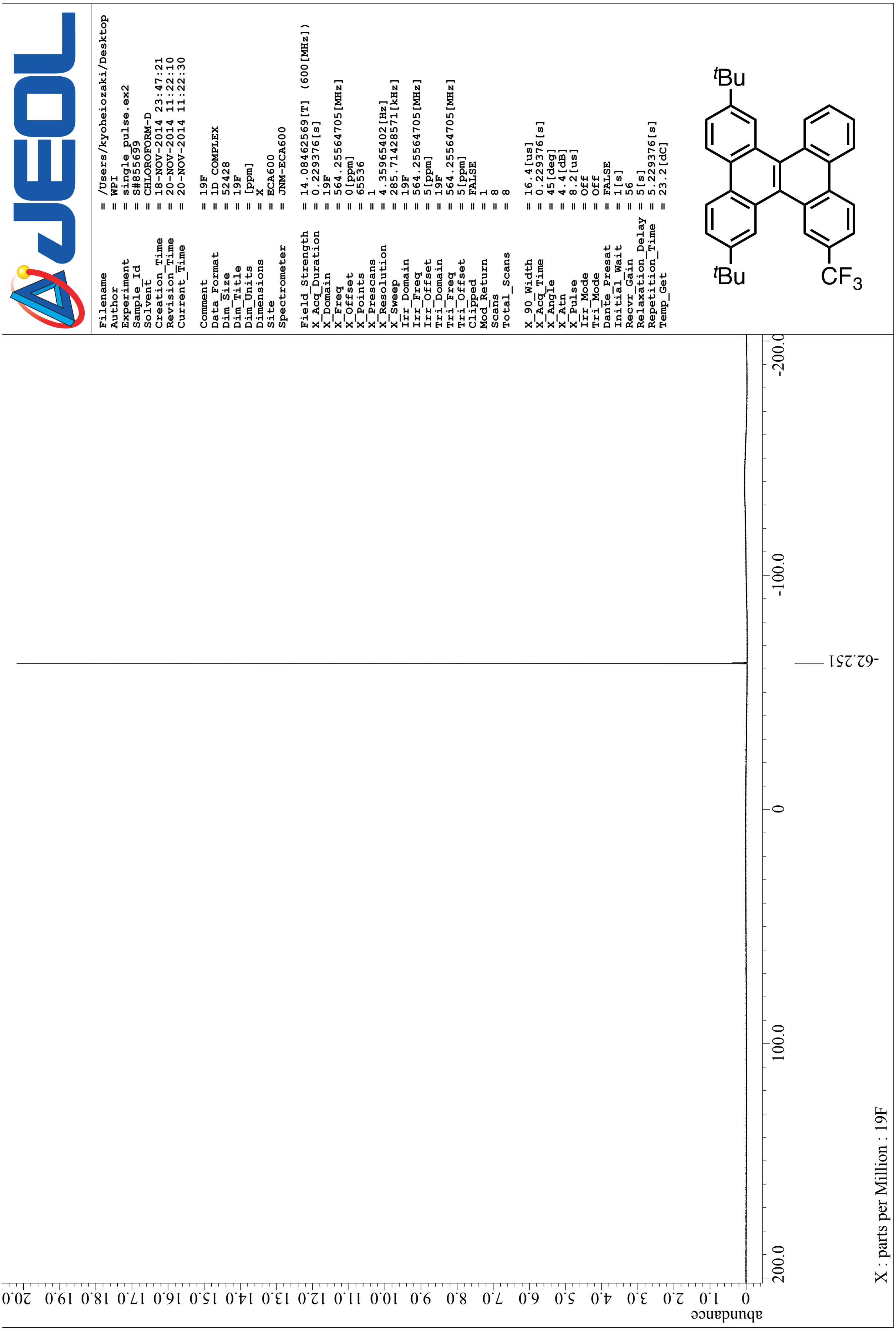

Supplementary Figure 9. ^{19}F NMR (565 MHz, $CDCl_3$) of 3ab

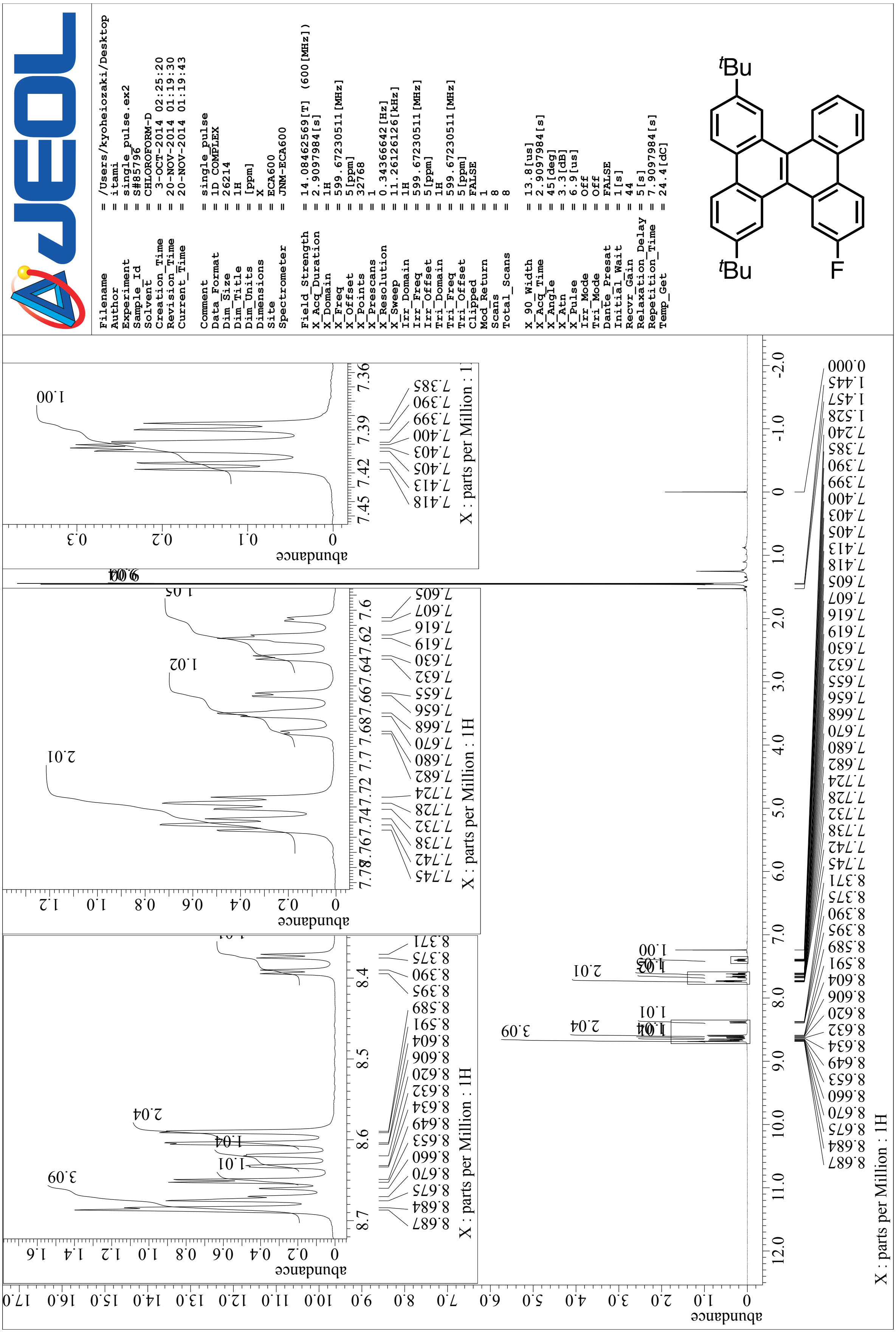

Supplementary Figure 10. ^{1}H NMR (600 MHz, $CDCl_3$) of 3ac

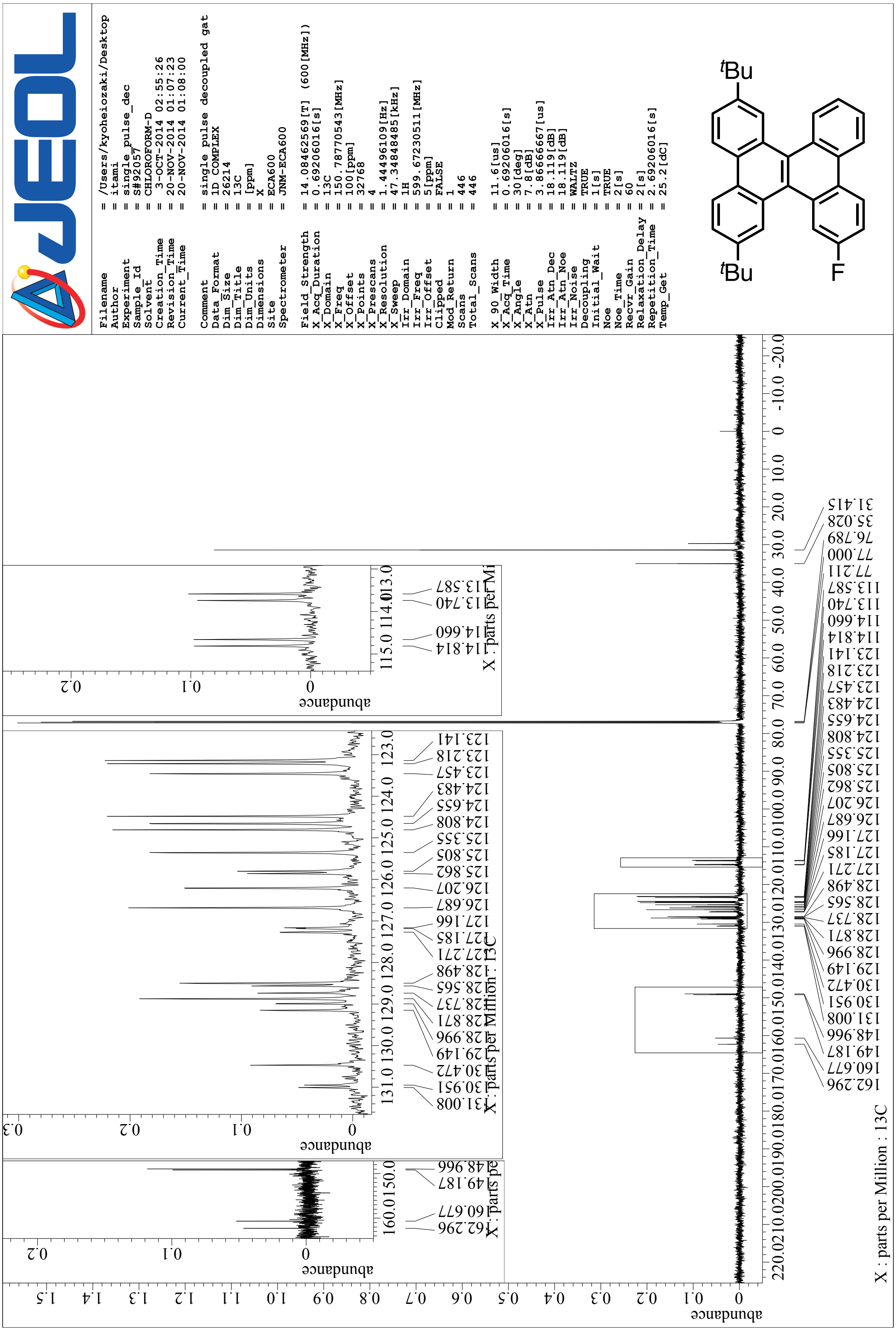

Supplementary Figure 11. ^{13}C NMR (150 MHz, $CDCl_3$) of **3ac**

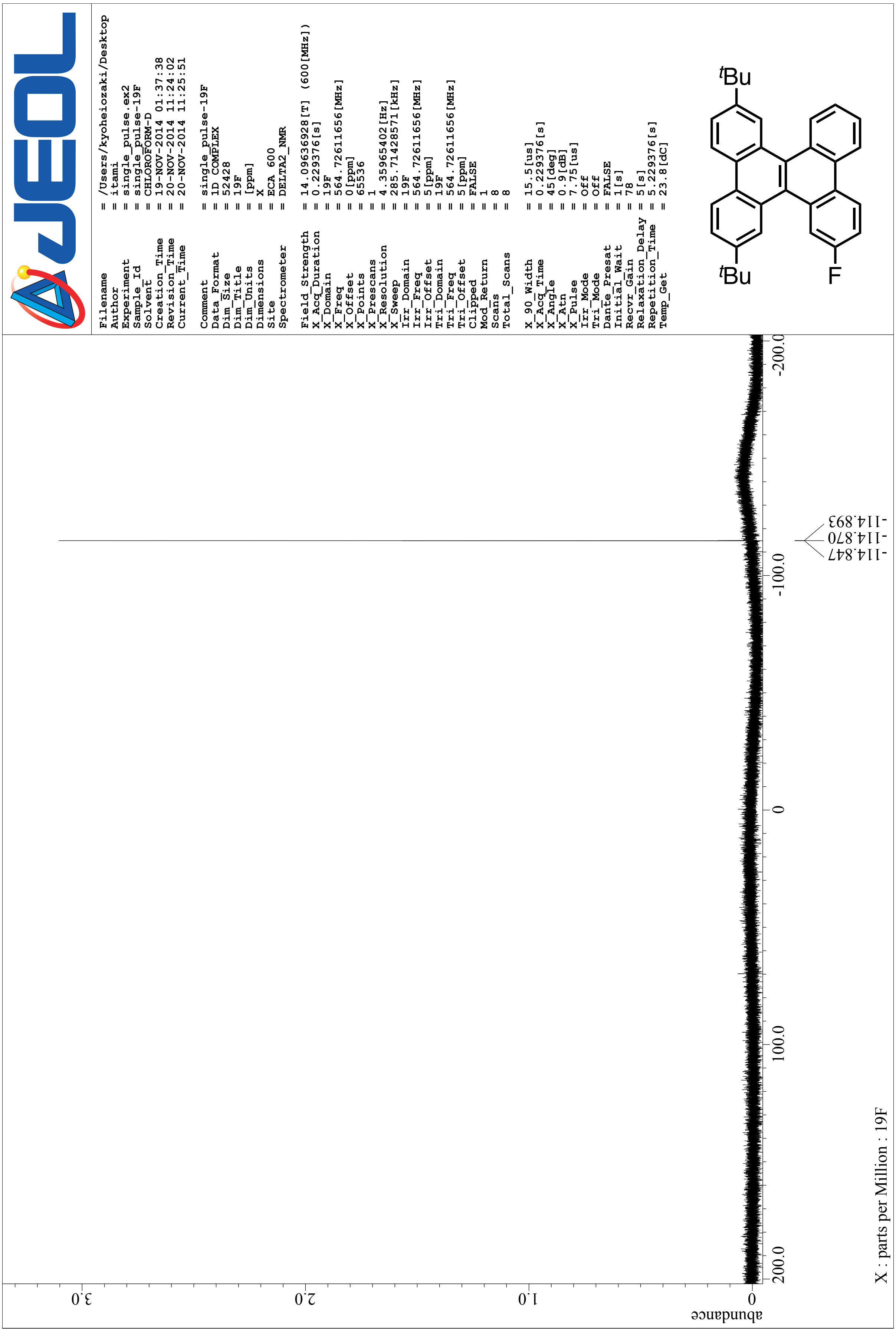

Supplementary Figure 12. ^{19}**F NMR (565 MHz, CDCl$_3$) of 3ac**

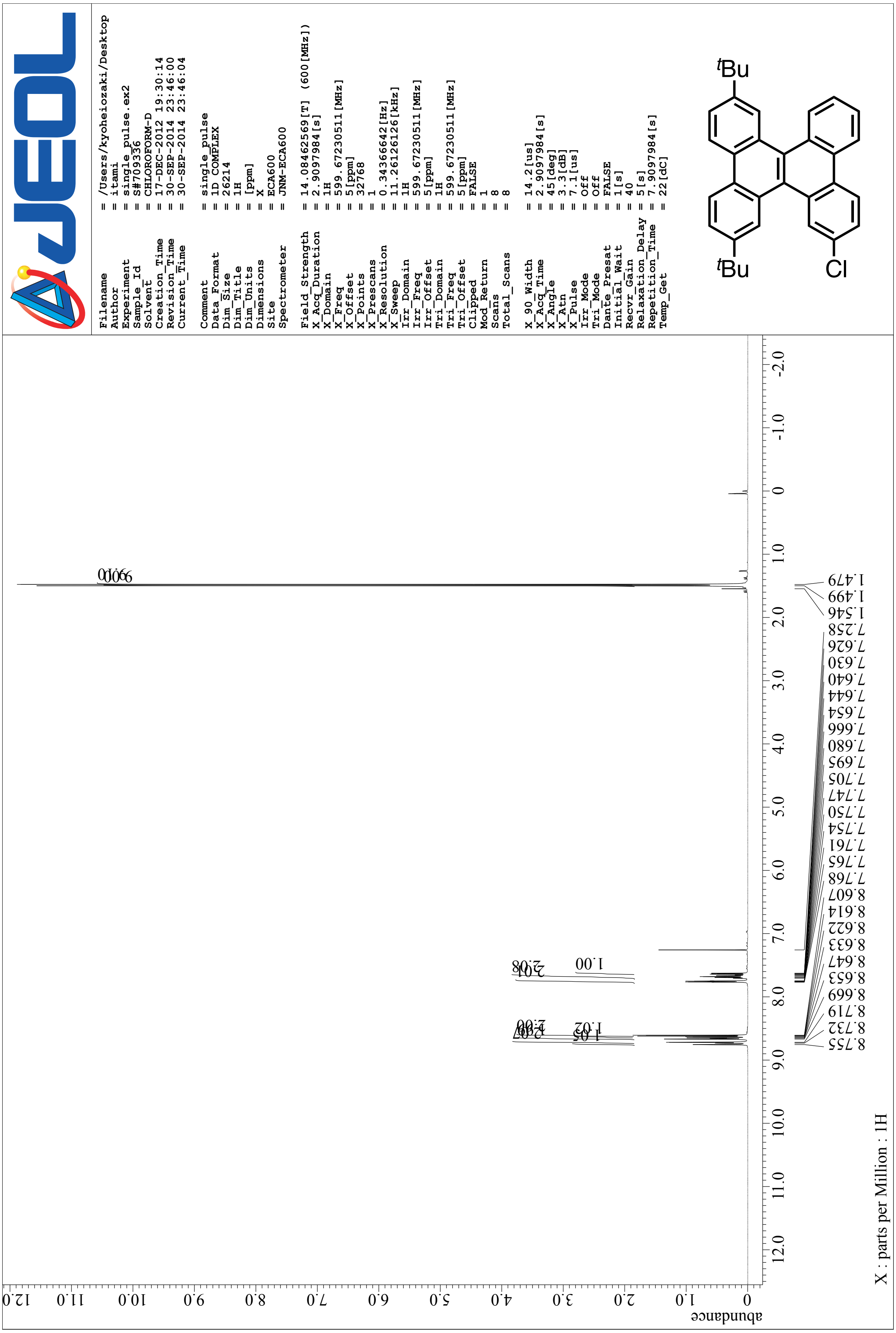

Supplementary Figure 13. ^{1}**H NMR (600 MHz,** $CDCl_3$**) of 3ad**

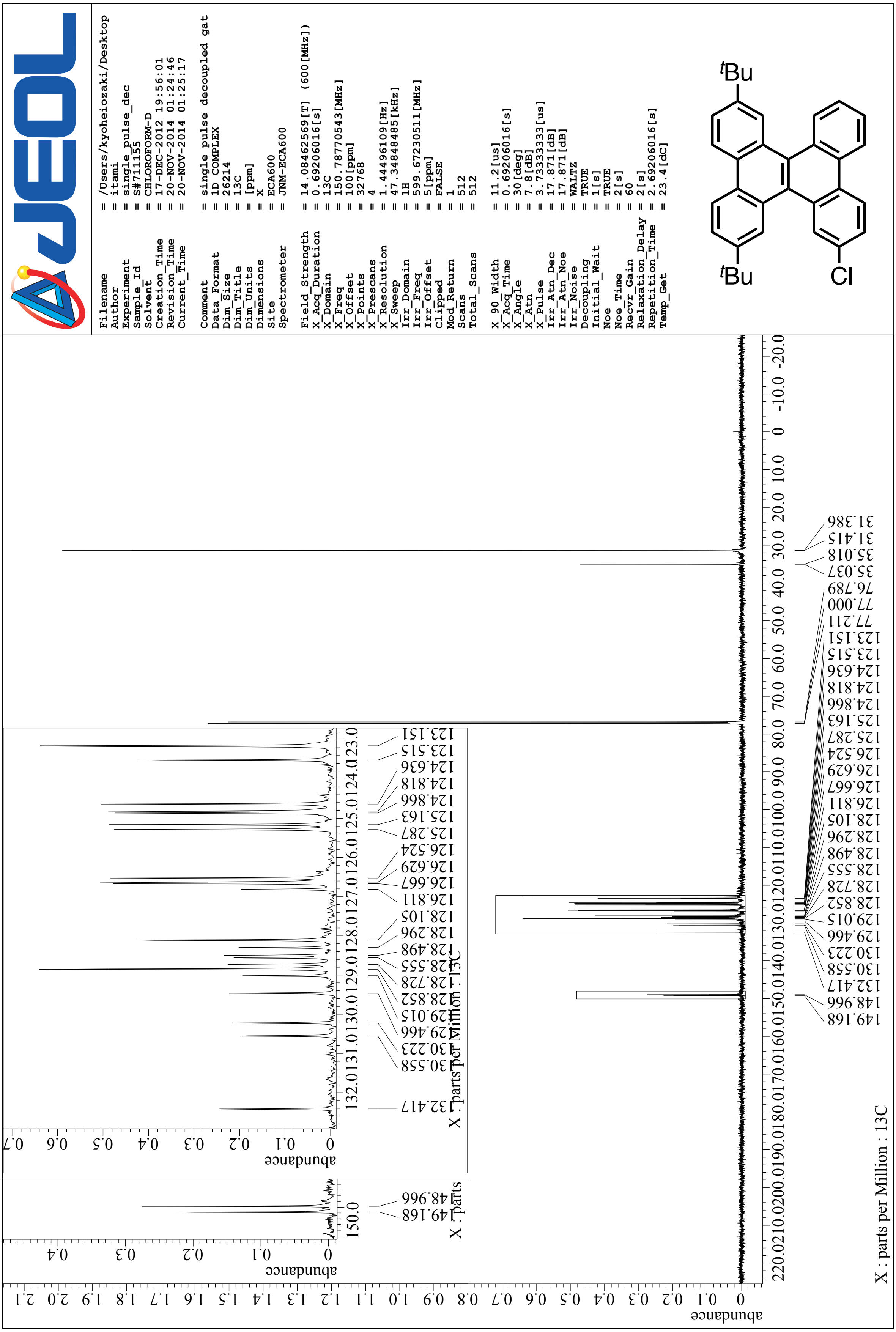

Supplementary Figure 14. ^{13}**C NMR (150 MHz,** $CDCl_3$**) of 3ad**

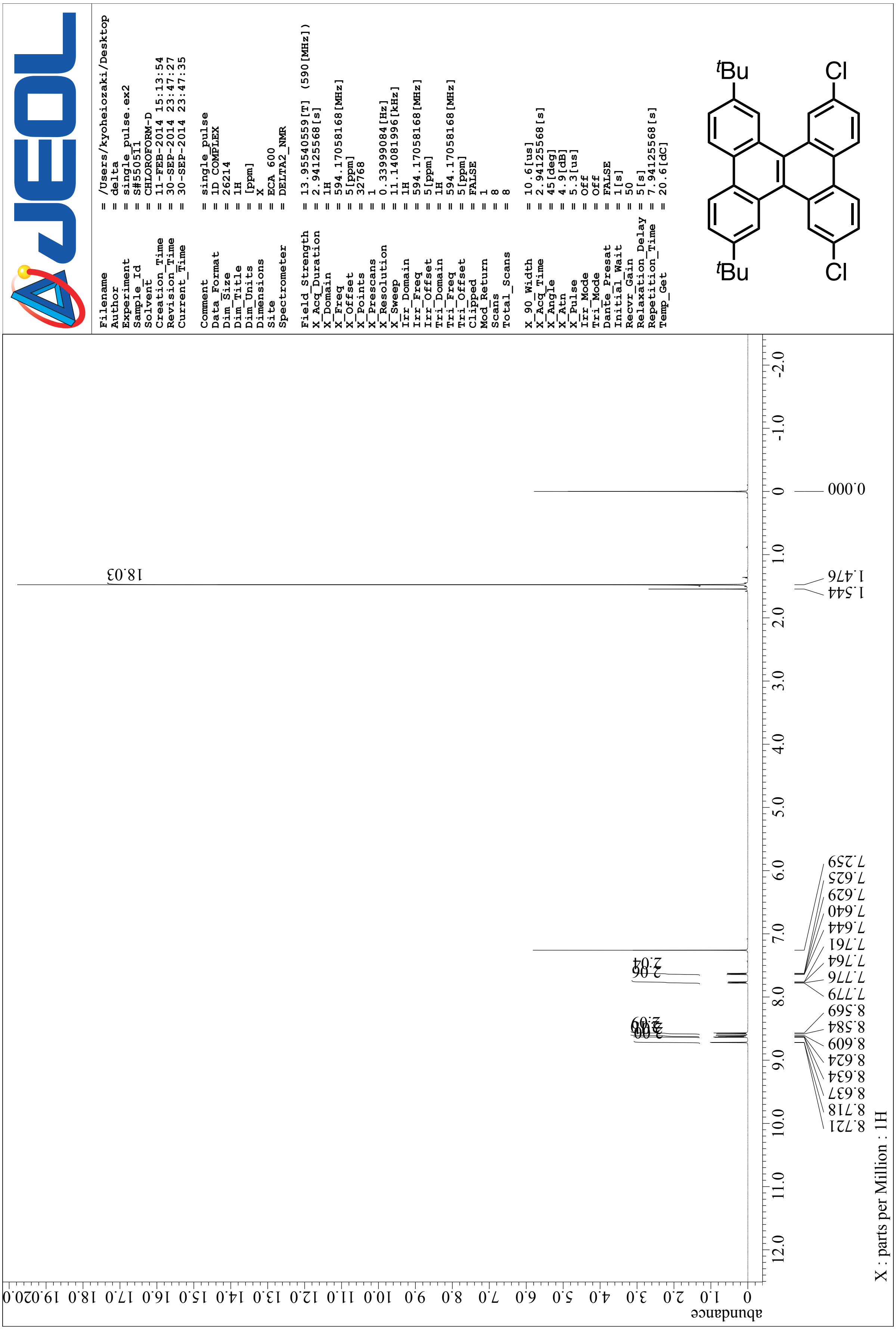

Supplementary Figure 15. ^{1}**H NMR (600 MHz,** $CDCl_3$**) of 3ae**

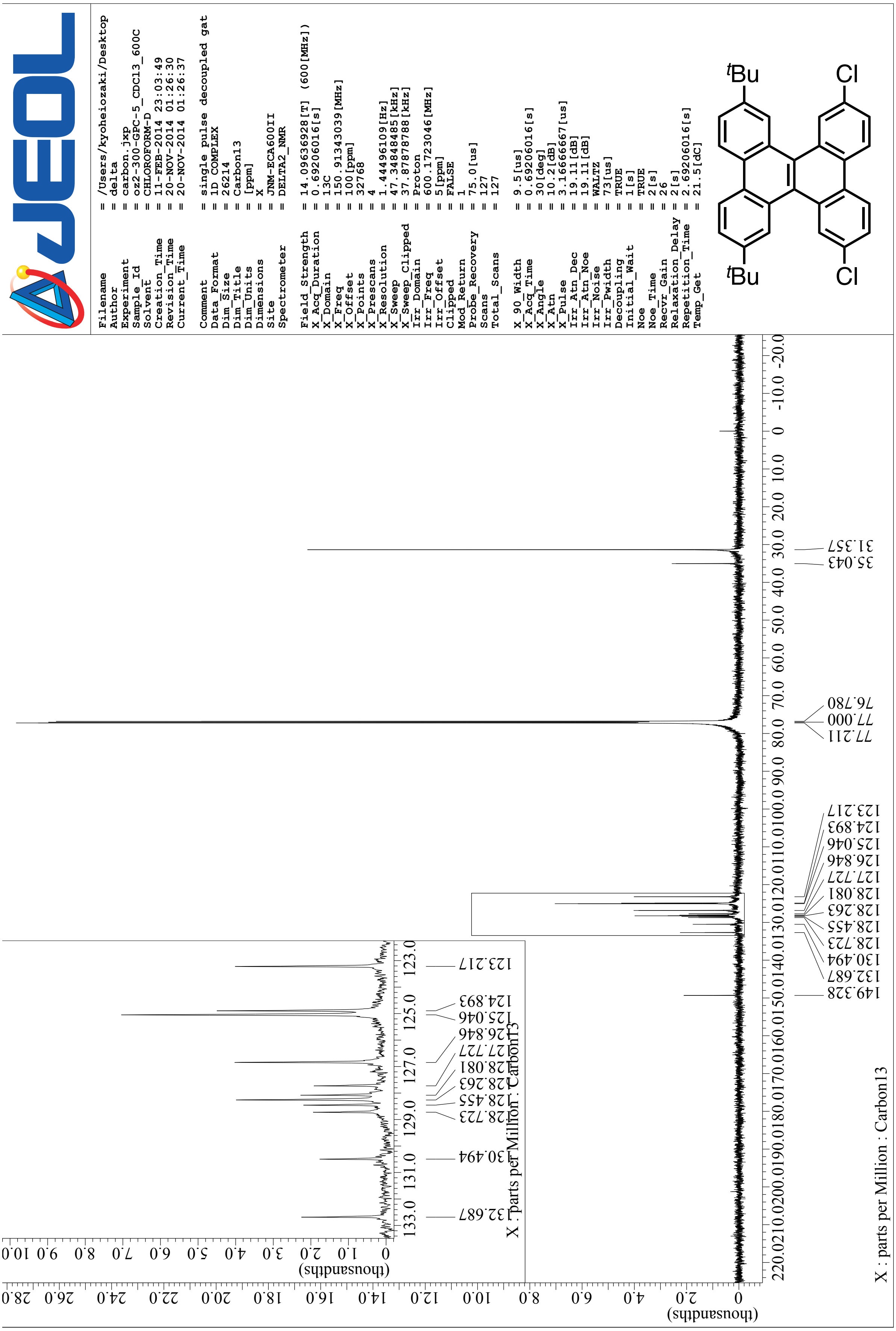

Supplementary Figure 16. ^{13}C NMR (150 MHz, $CDCl_3$) of **3ae**

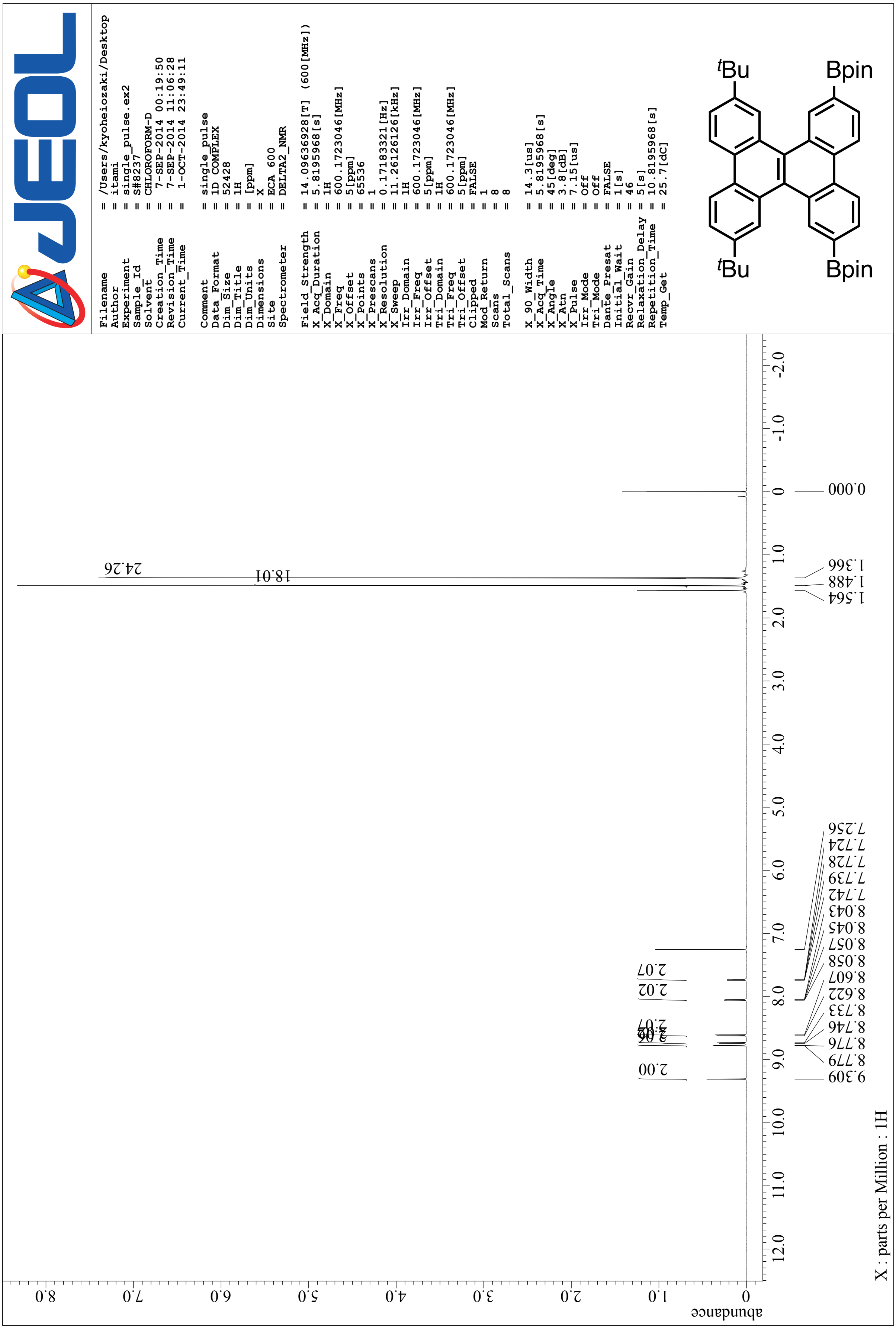

Supplementary Figure 17. ^{1}H NMR (600 MHz, $CDCl_3$) of 3af

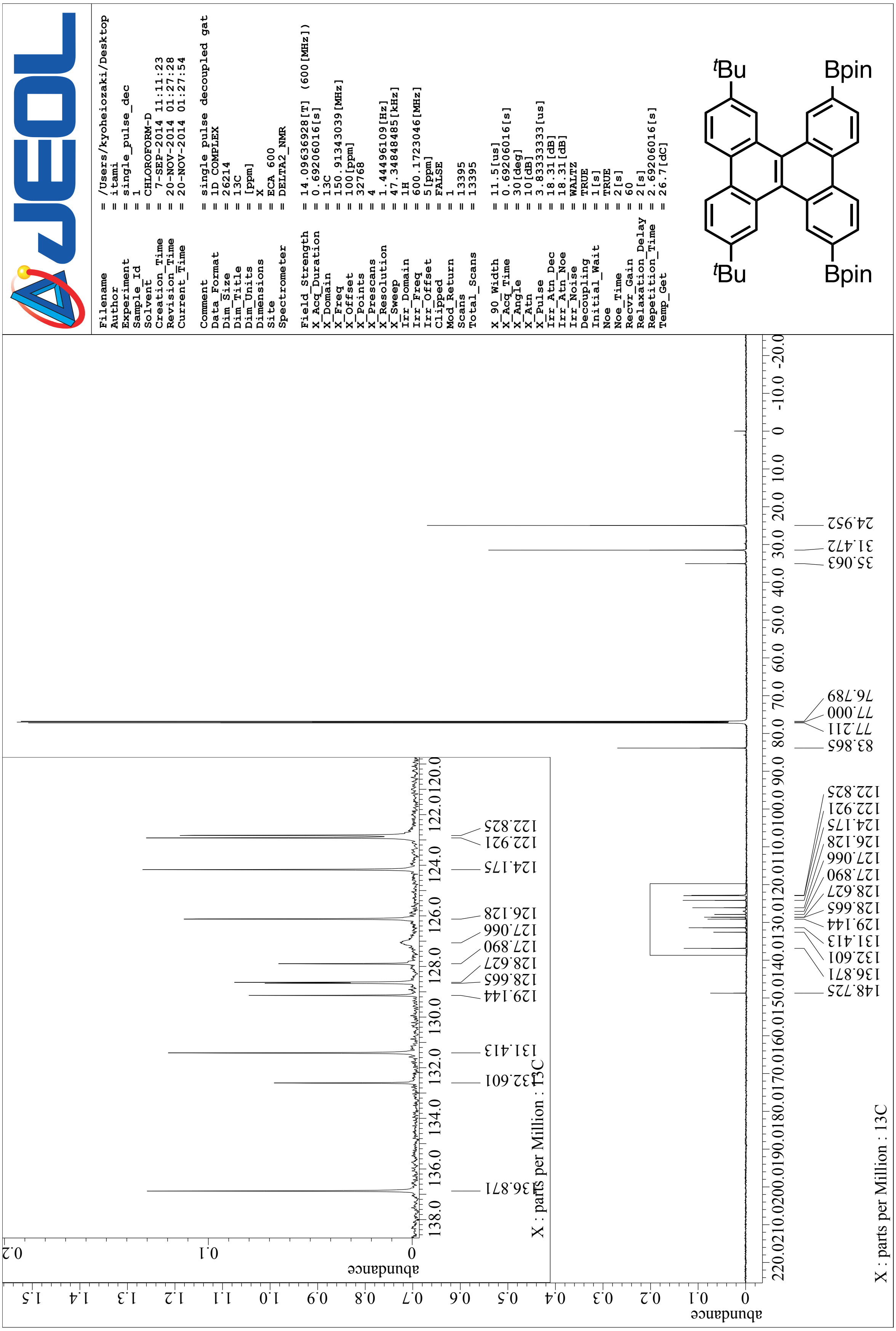

Supplementary Figure 18. ^{13}**C NMR (150 MHz, CDCl$_3$) of 3af**

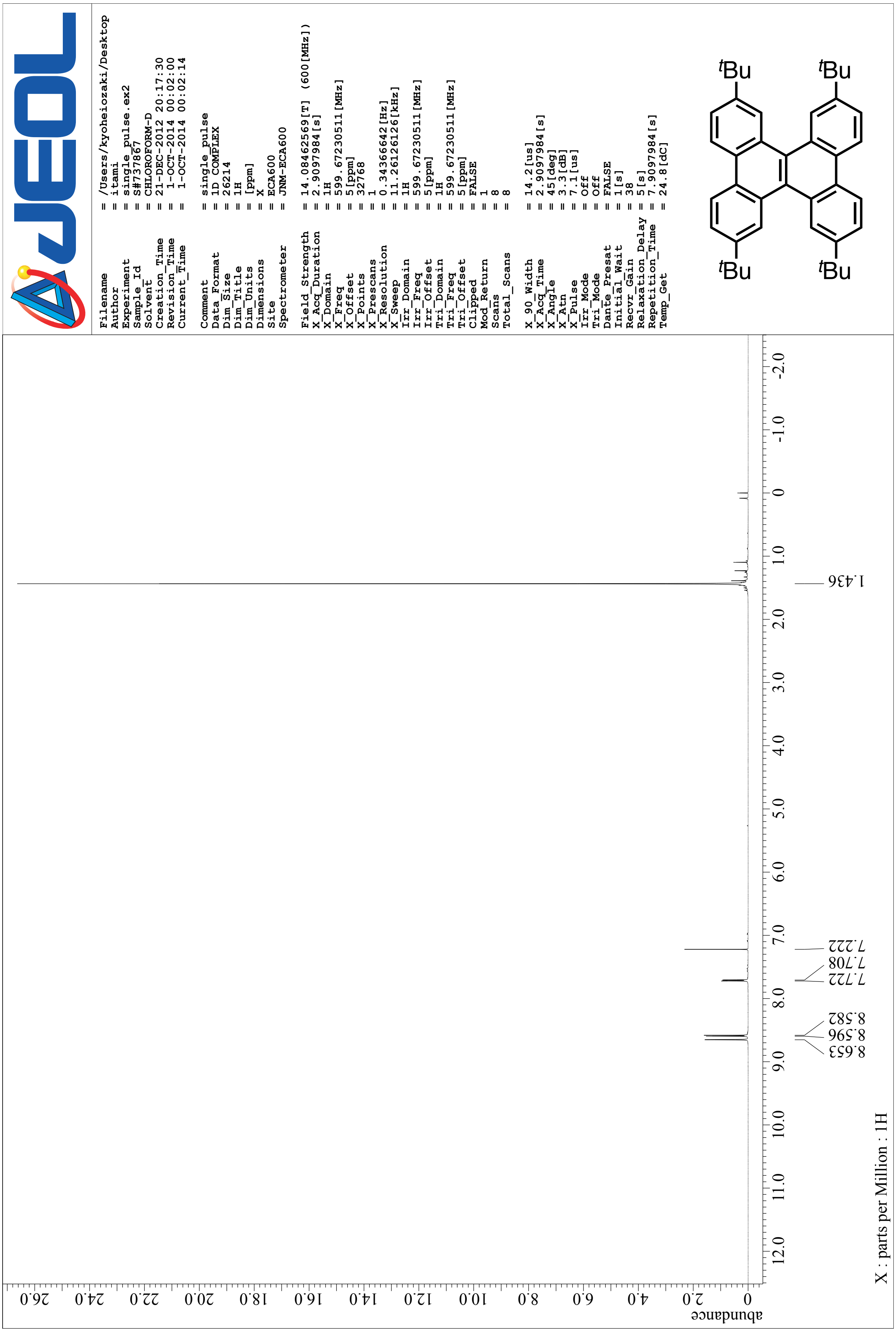

Supplementary Figure 19. ^{1}H NMR (600 MHz, $CDCl_3$) of 3ag

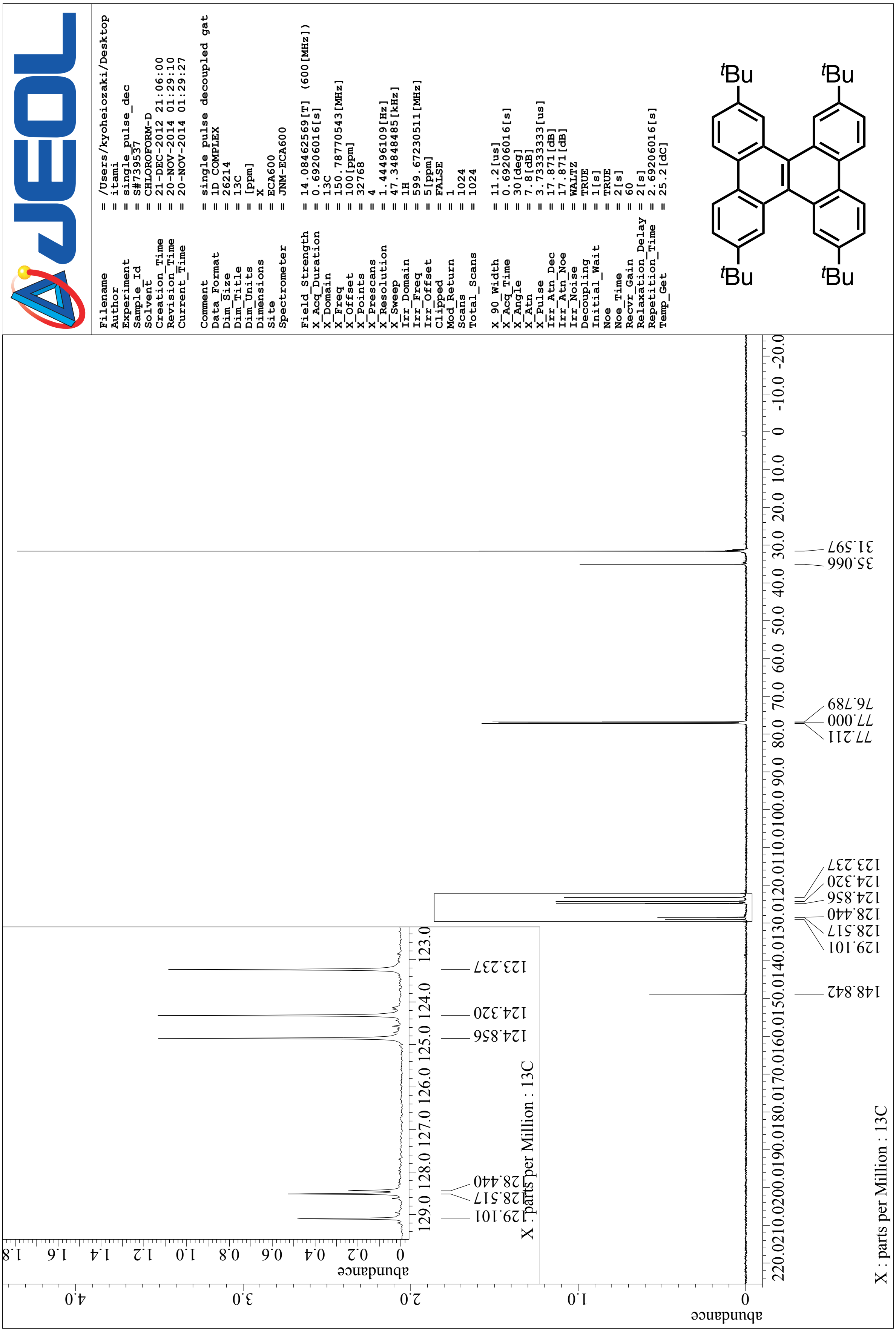

Supplementary Figure 20. ^{13}**C NMR (150 MHz, CDCl$_3$) of 3ag**

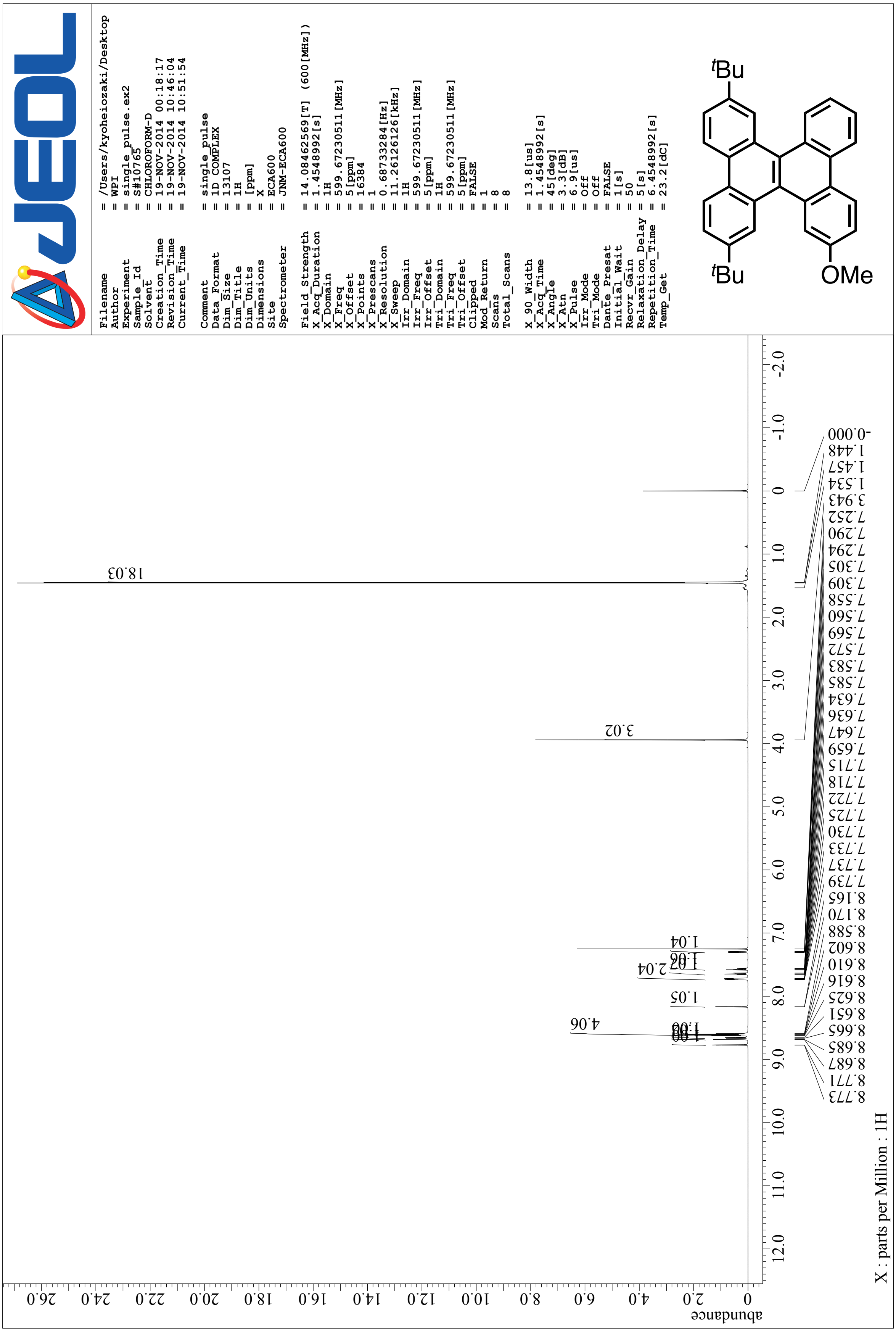

Supplementary Figure 21. ^{1}H NMR (600 MHz, $CDCl_3$) of 3ah

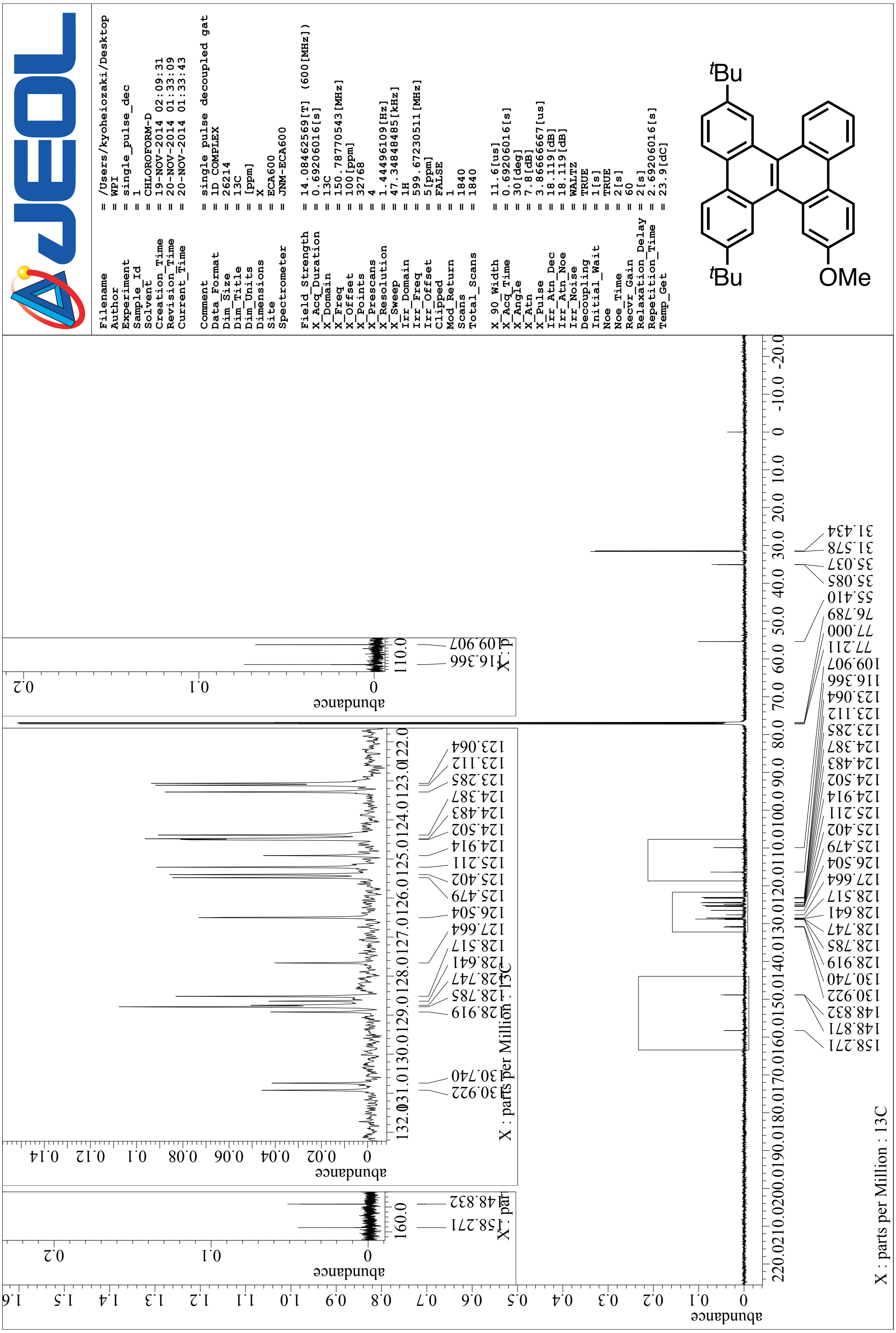

Supplementary Figure 22. ^{13}C NMR (150 MHz, $CDCl_3$) of **3ah**

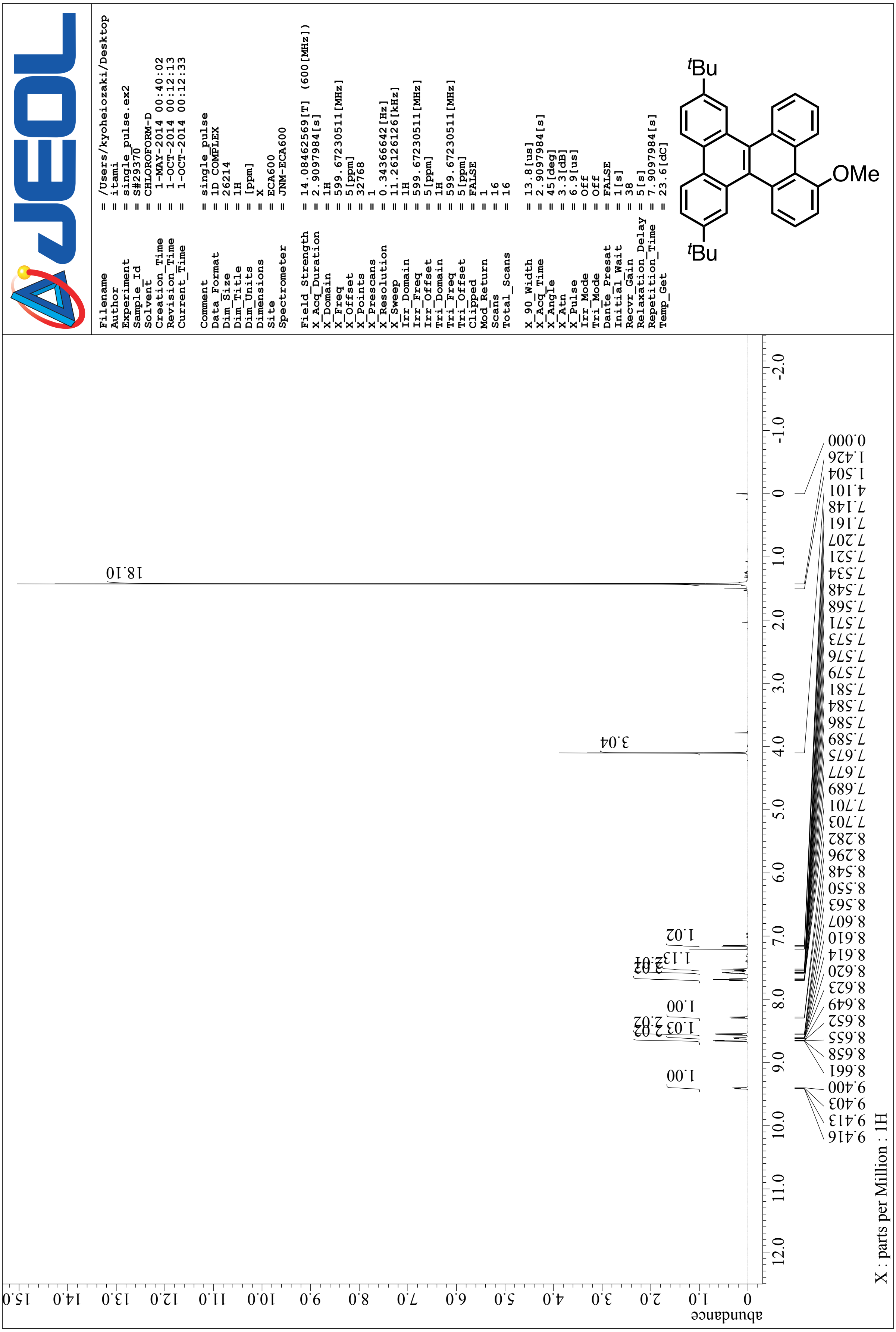

Supplementary Figure 23. ^{1}H **NMR (600 MHz,** $CDCl_3$**) of 3ai**

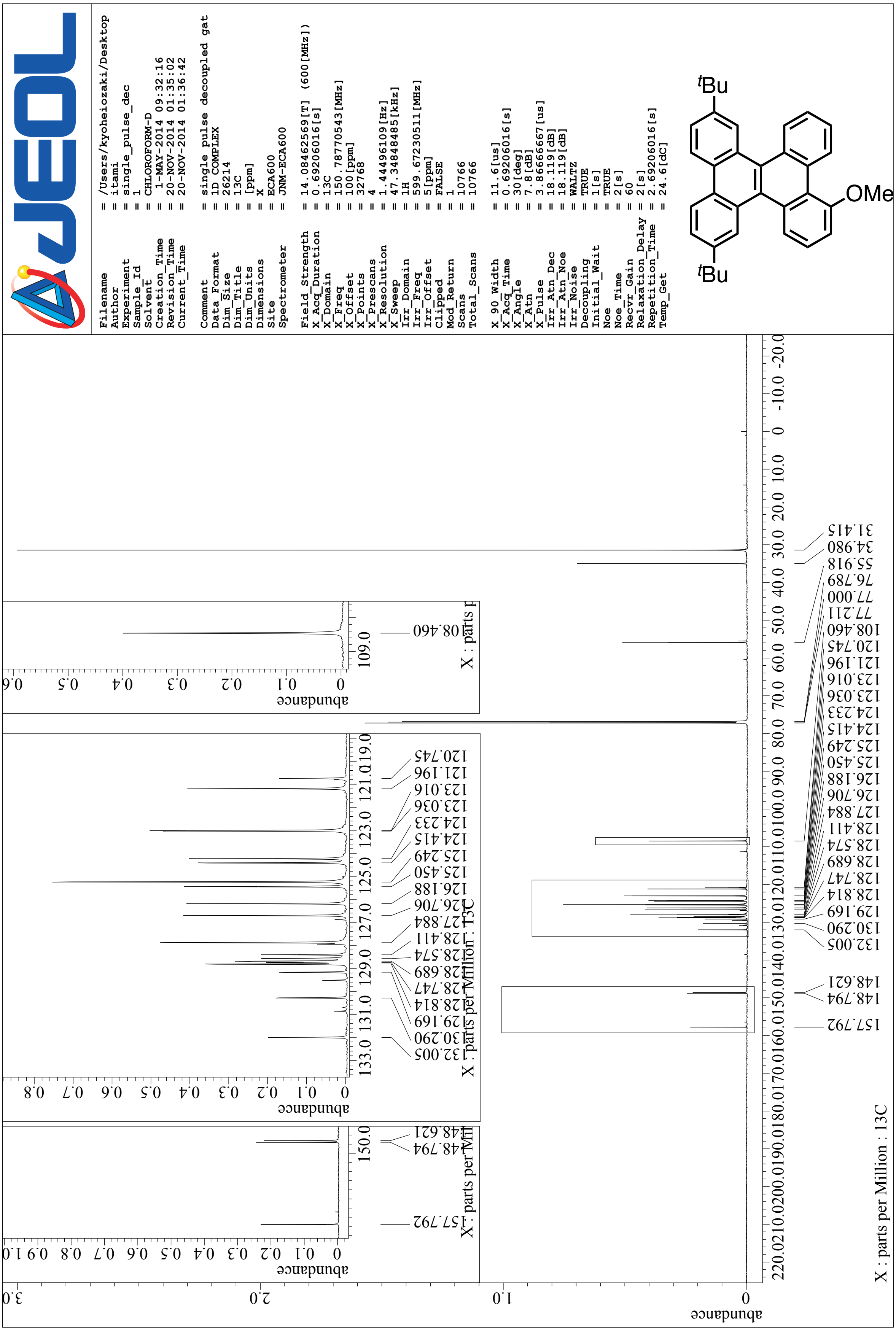

Supplementary Figure 24. ^{13}C NMR (150 MHz, $CDCl_3$) of 3ai

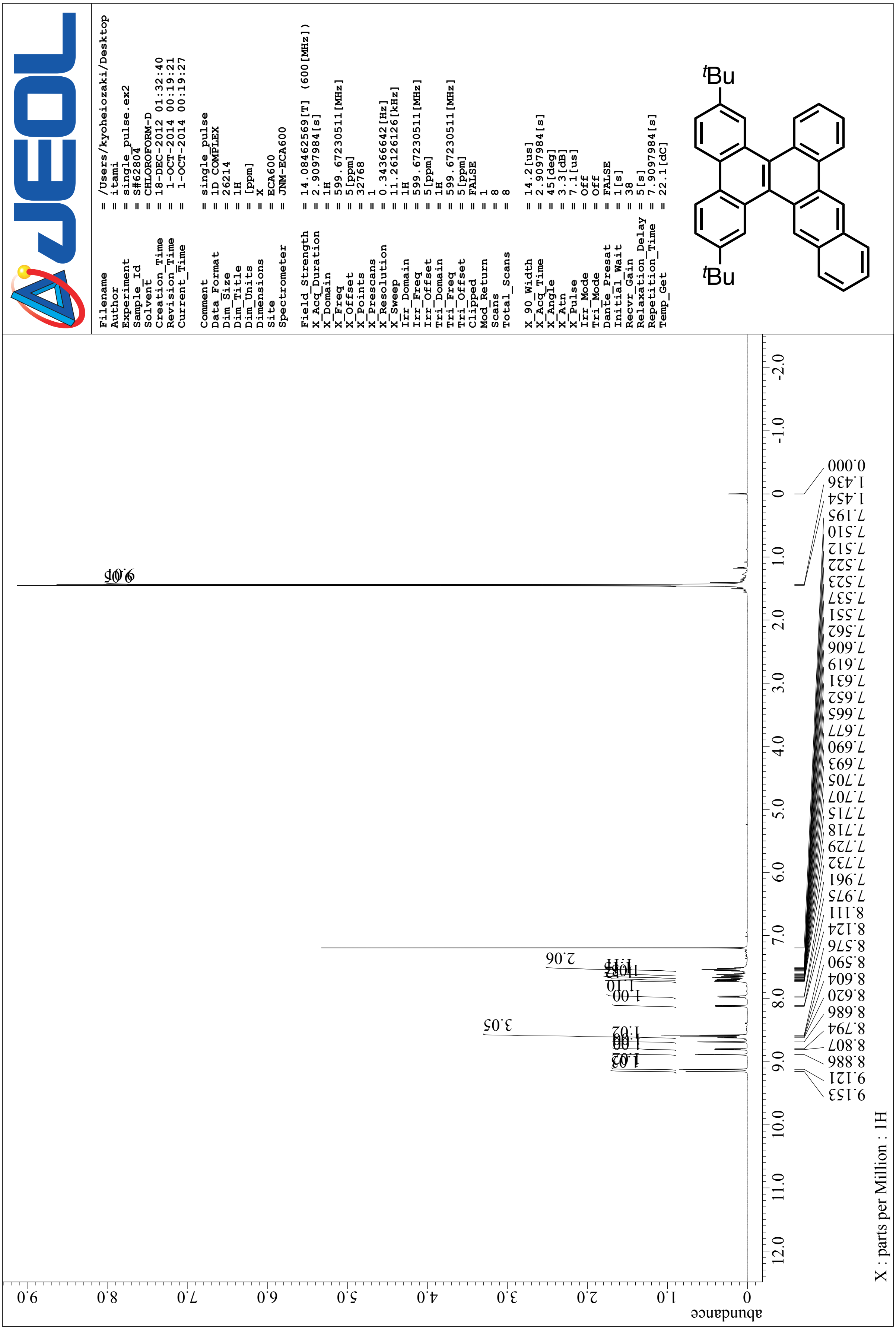

Supplementary Figure 25. ^{1}H NMR (600 MHz, $CDCl_3$) of 3aj

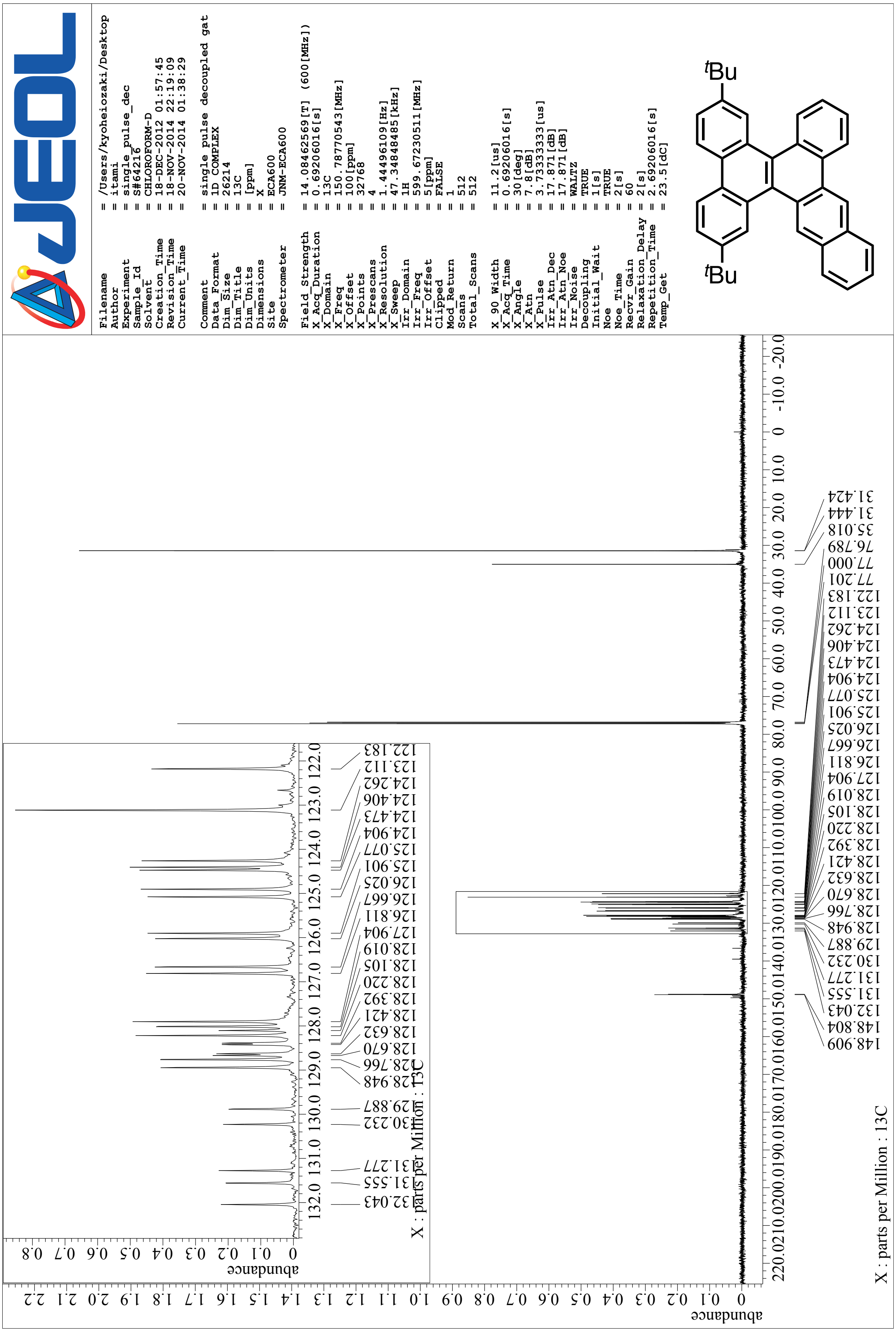

Supplementary Figure 26. ^{13}**C NMR (150 MHz, CDCl$_3$) of 3aj**

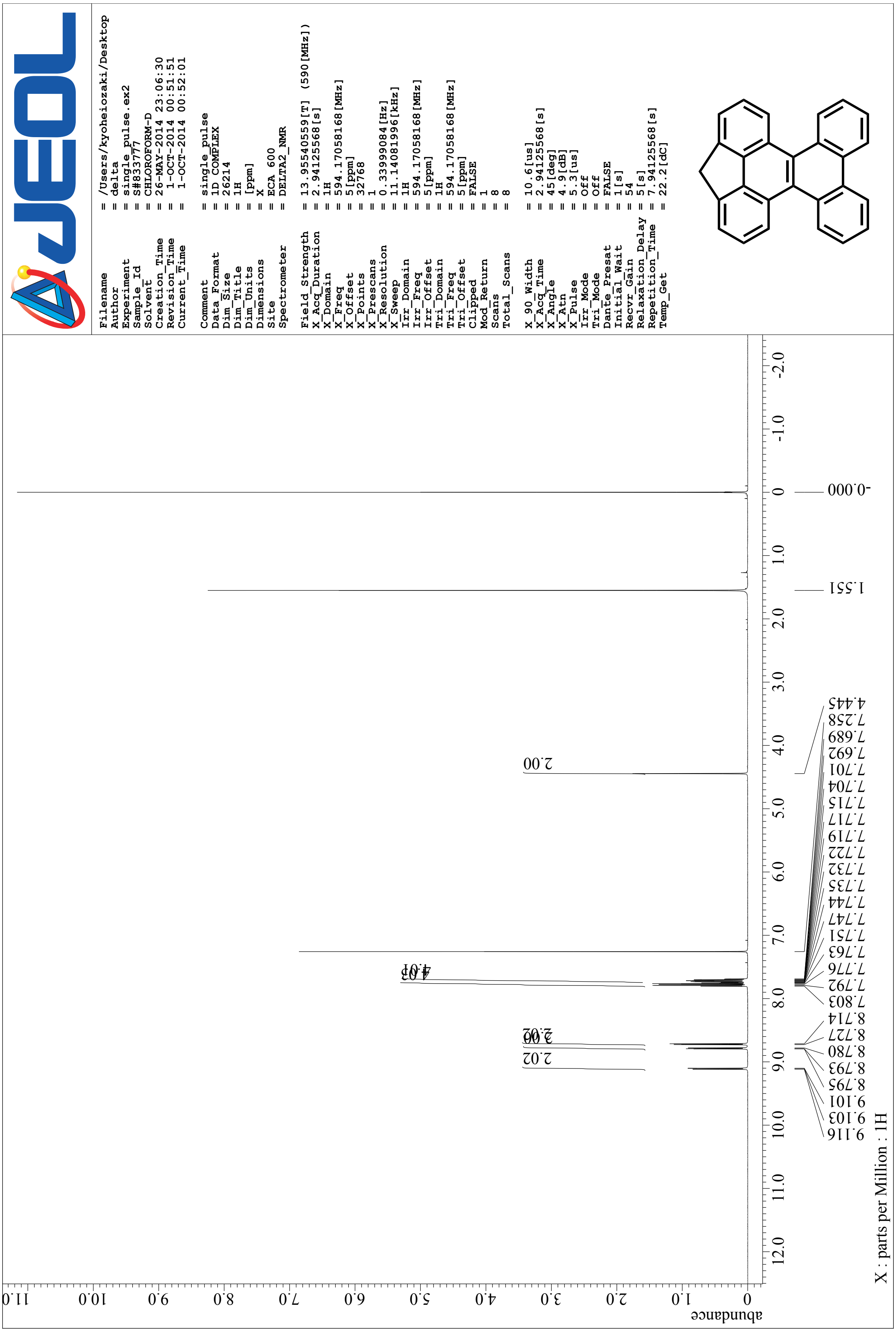

Supplementary Figure 27. ^{1}H NMR (600 MHz, $CDCl_3$) of 3ca

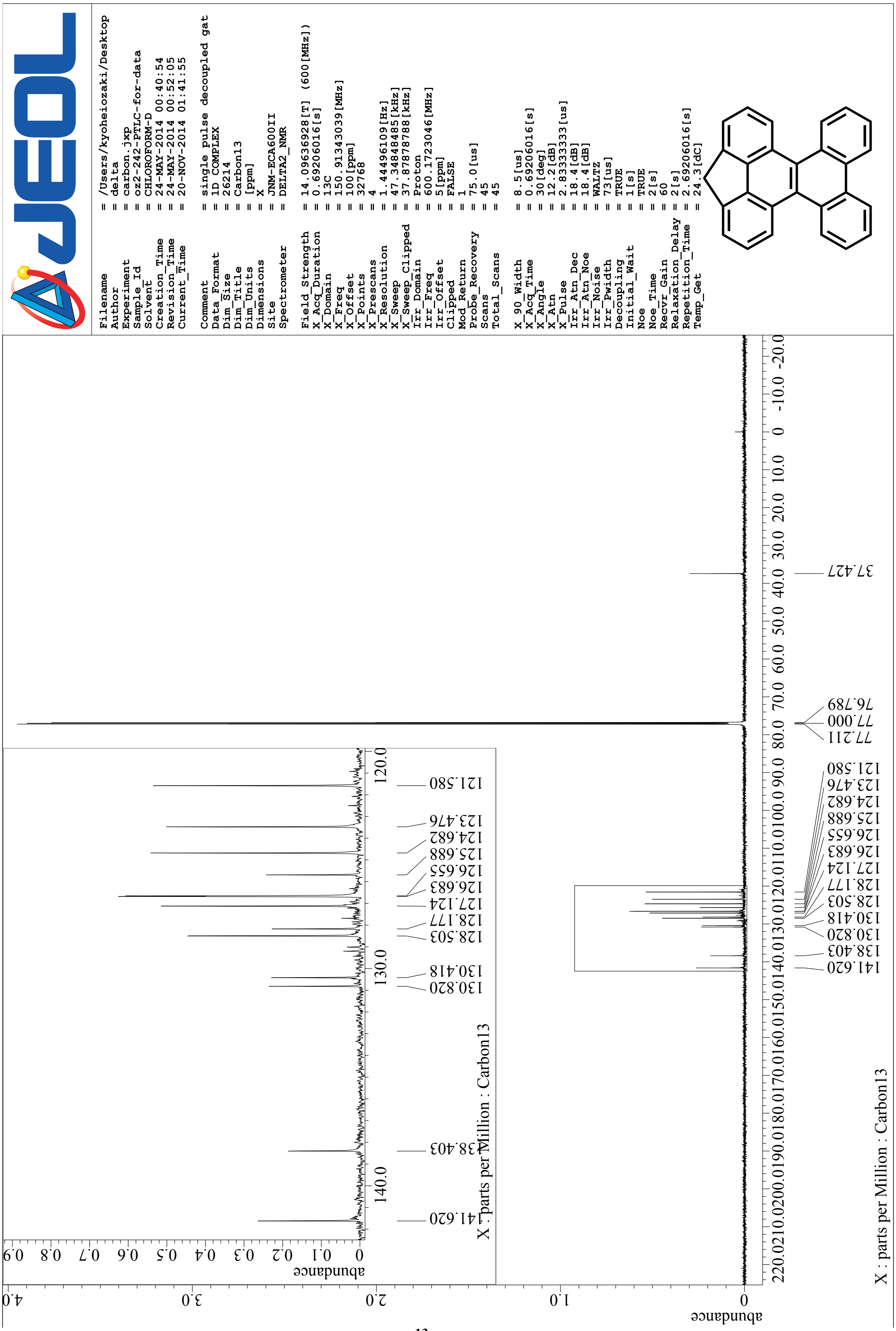

Supplementary Figure 28. ^{13}**C NMR (150 MHz, CDCl$_3$) of 3ca**

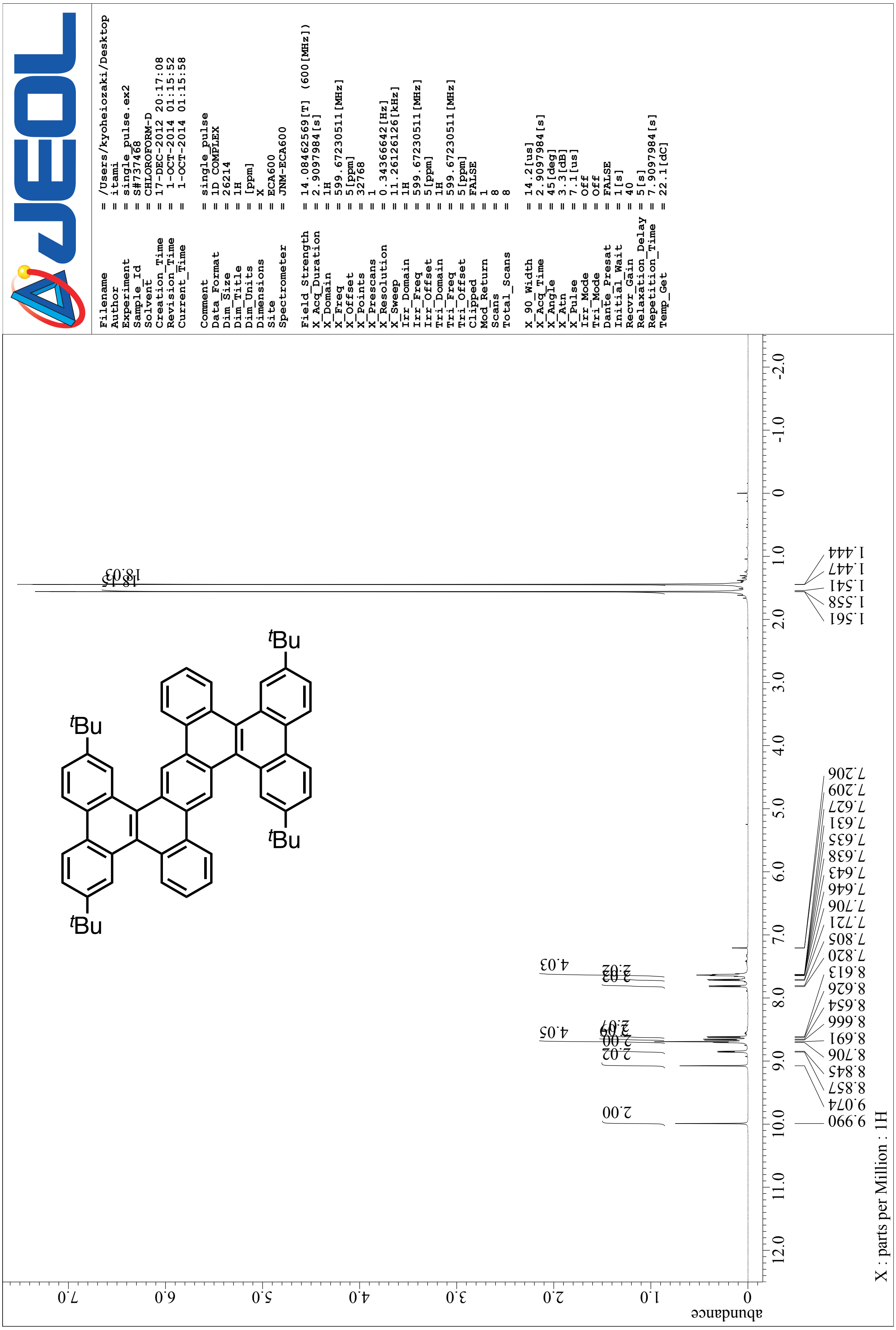

Supplementary Figure 29. ^{1}**H NMR (600 MHz, $CDCl_3/CS_2$) of 5**

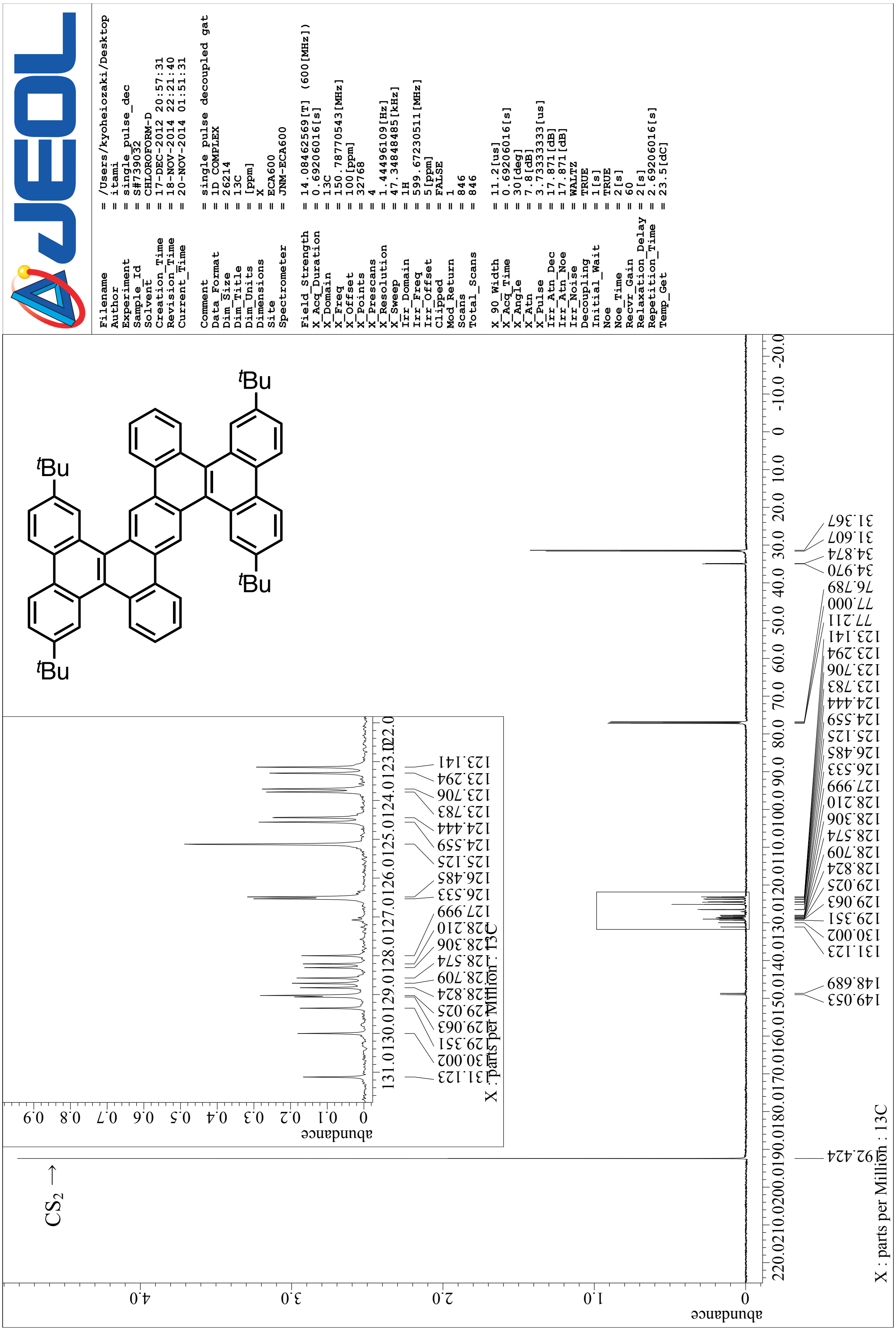

Supplementary Figure 30. ^{13}**C NMR (150 MHz,** $CDCl_3/CS_2$**) of 5**

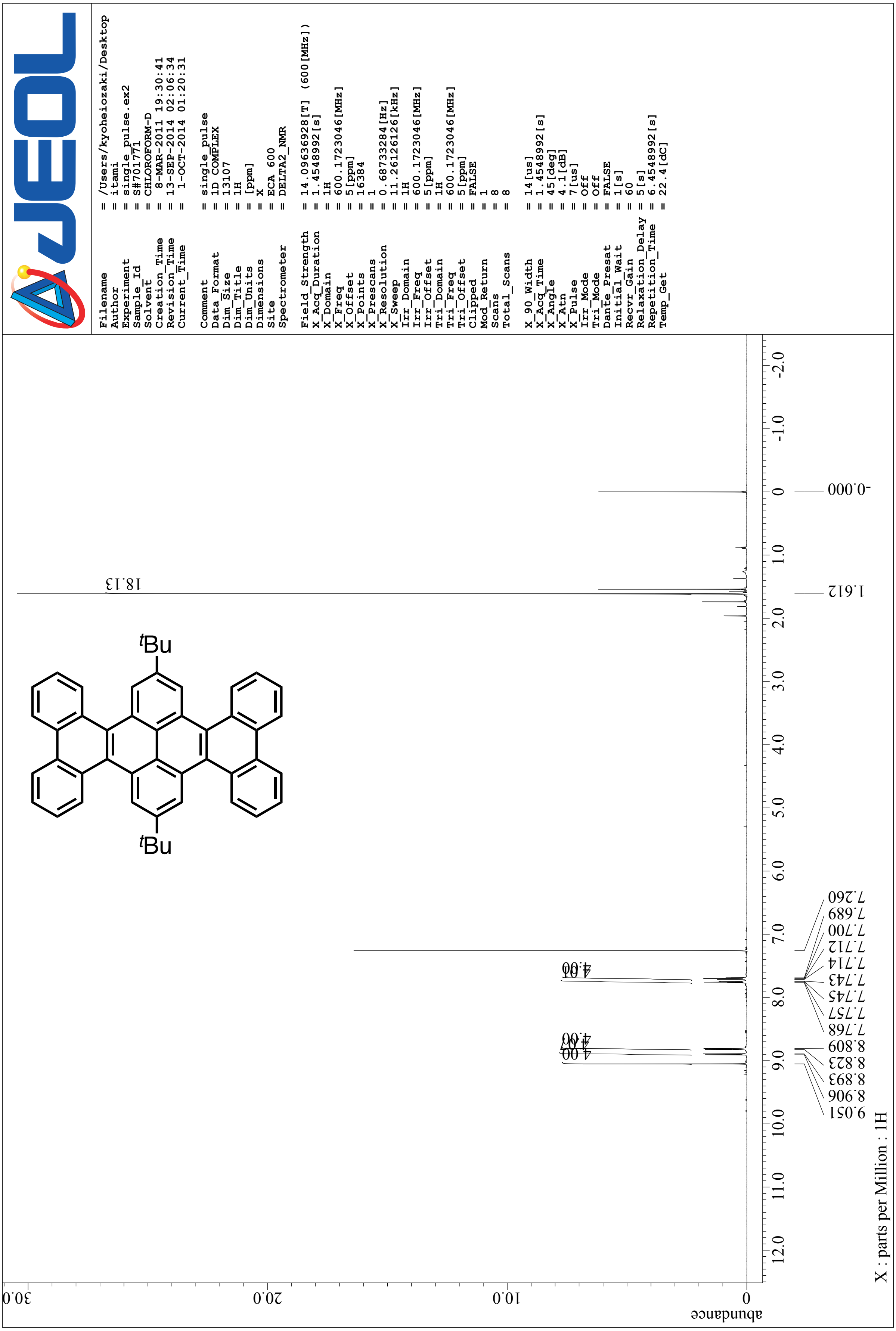

Supplementary Figure 31. ^{1}**H NMR (600 MHz, CDCl$_3$) of 7**

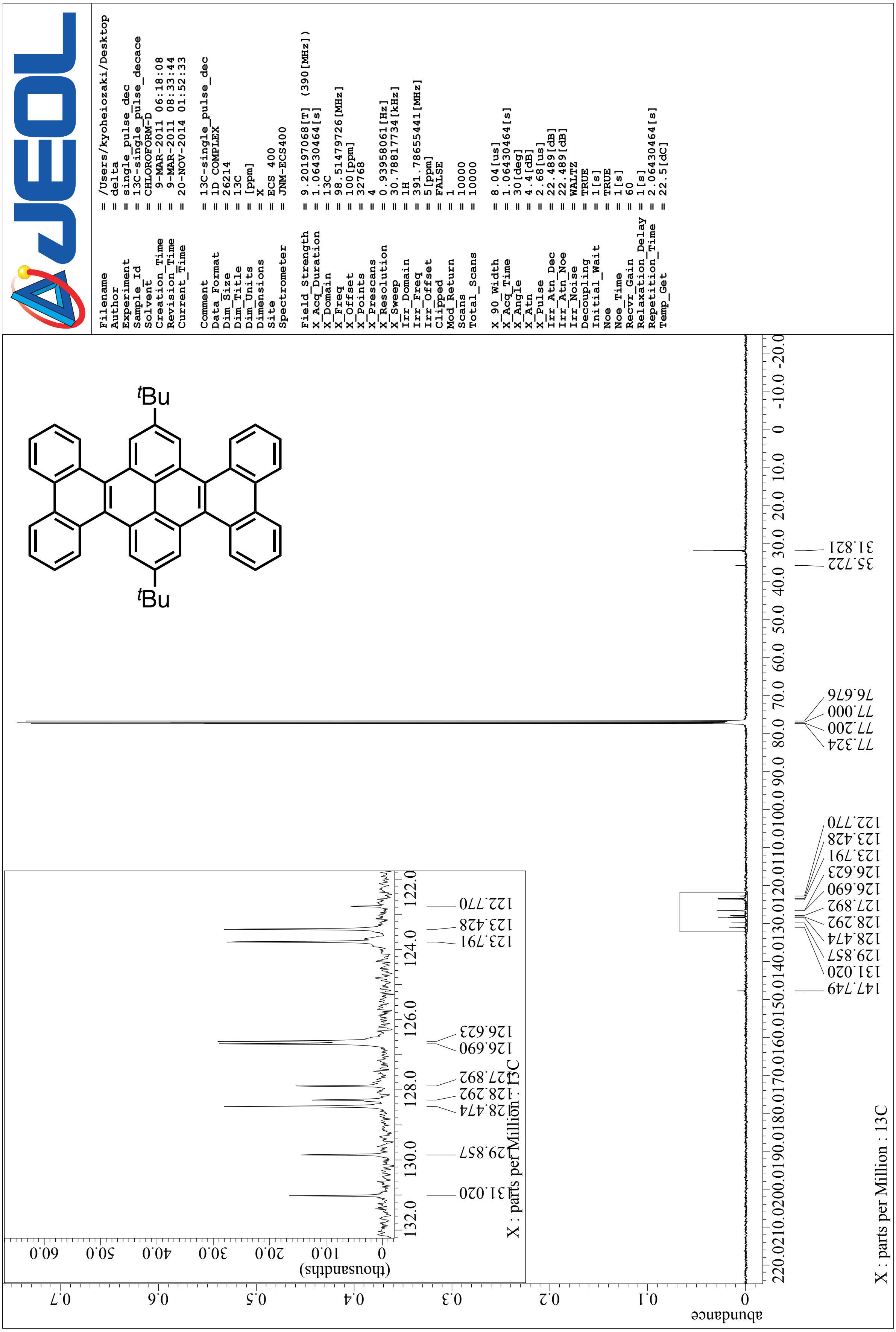

Supplementary Figure 32. ^{13}**C NMR (150 MHz, CDCl$_3$) of 7**

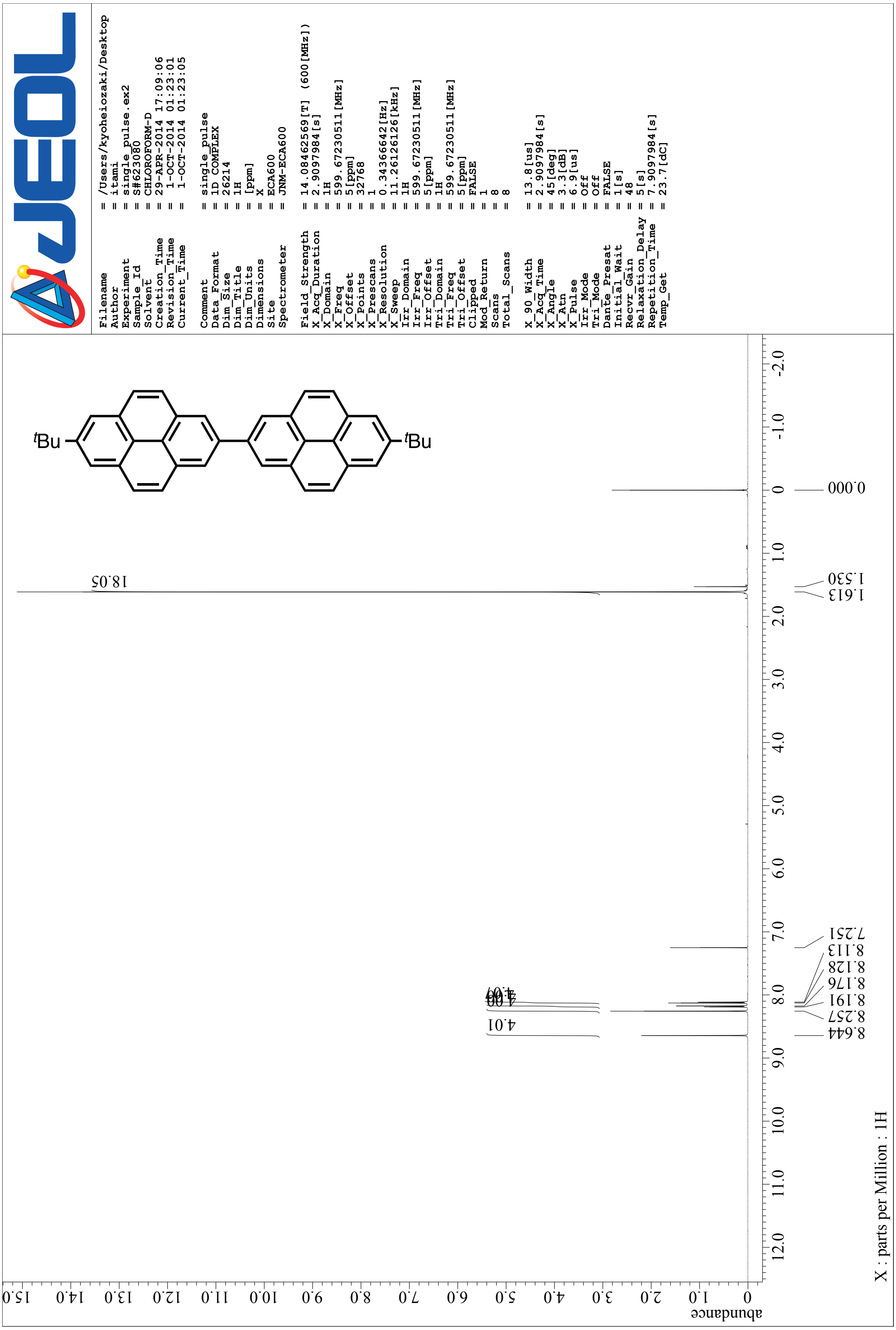

Supplementary Figure 33. ^{1}H NMR (600 MHz, $CDCl_3$) of 8

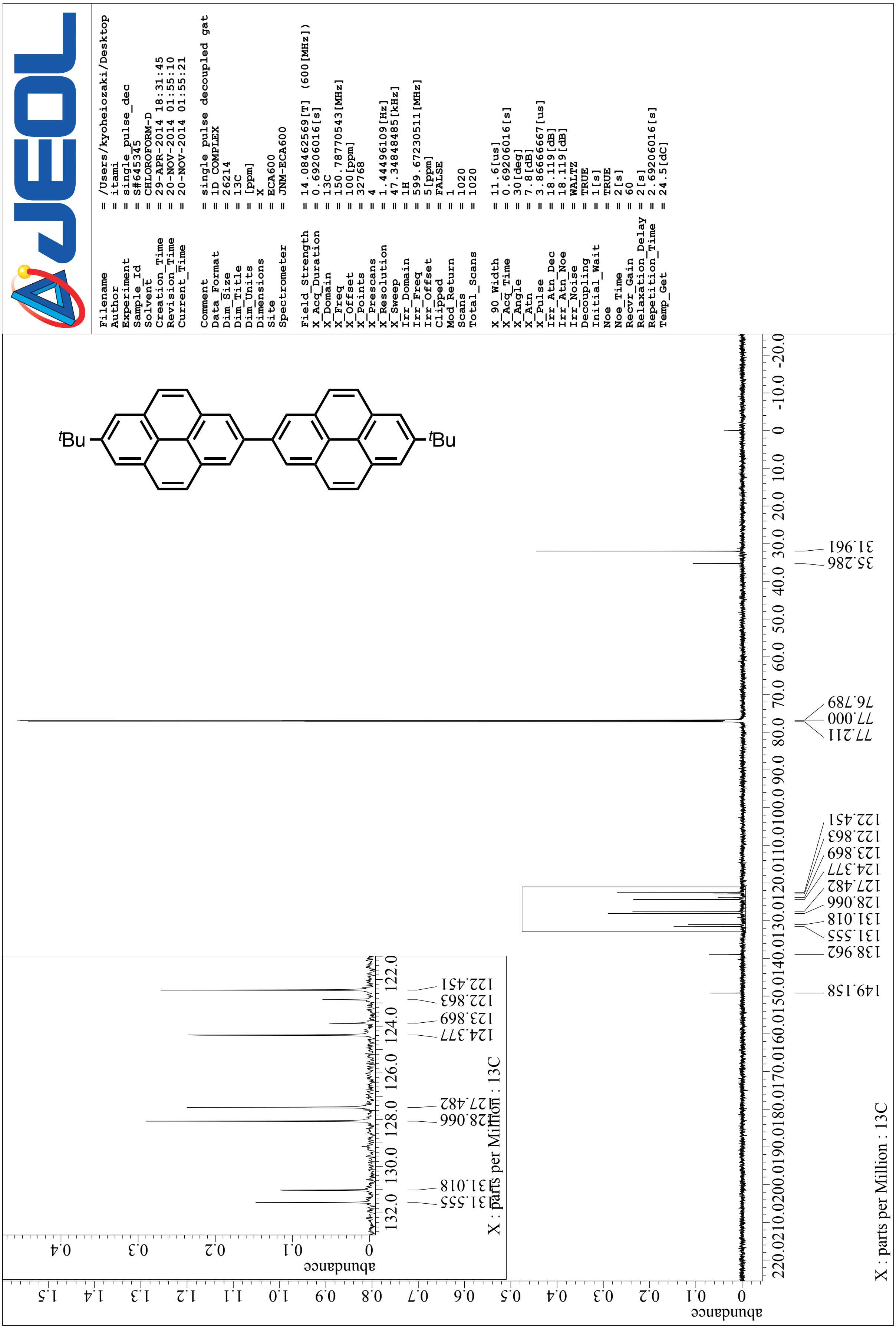

Supplementary Figure 34. ^{13}C **NMR (150 MHz,** $CDCl_3$**) of 8**

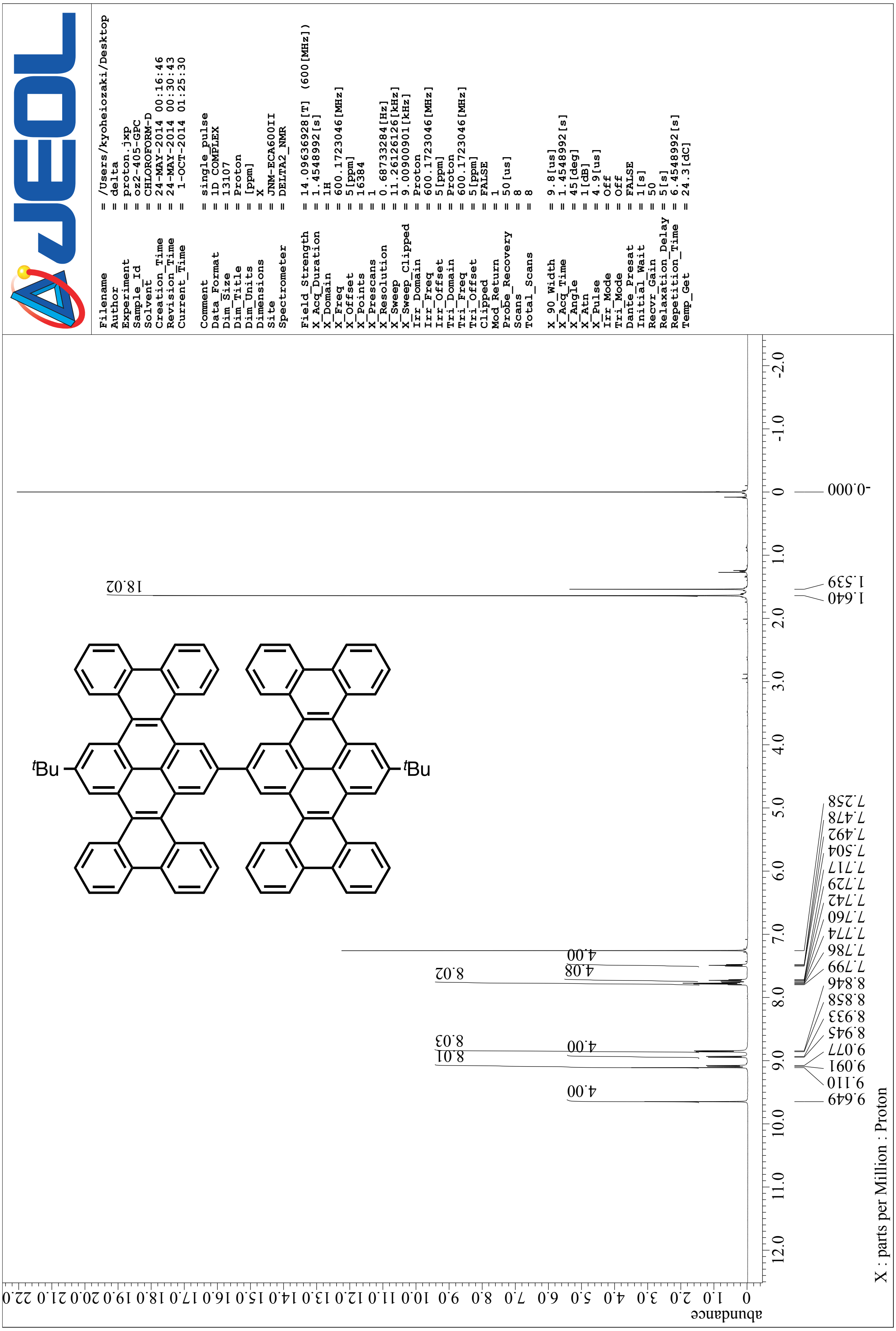

Supplementary Figure 35. ^{1}**H NMR (600 MHz,** $CDCl_3$**) of 9**

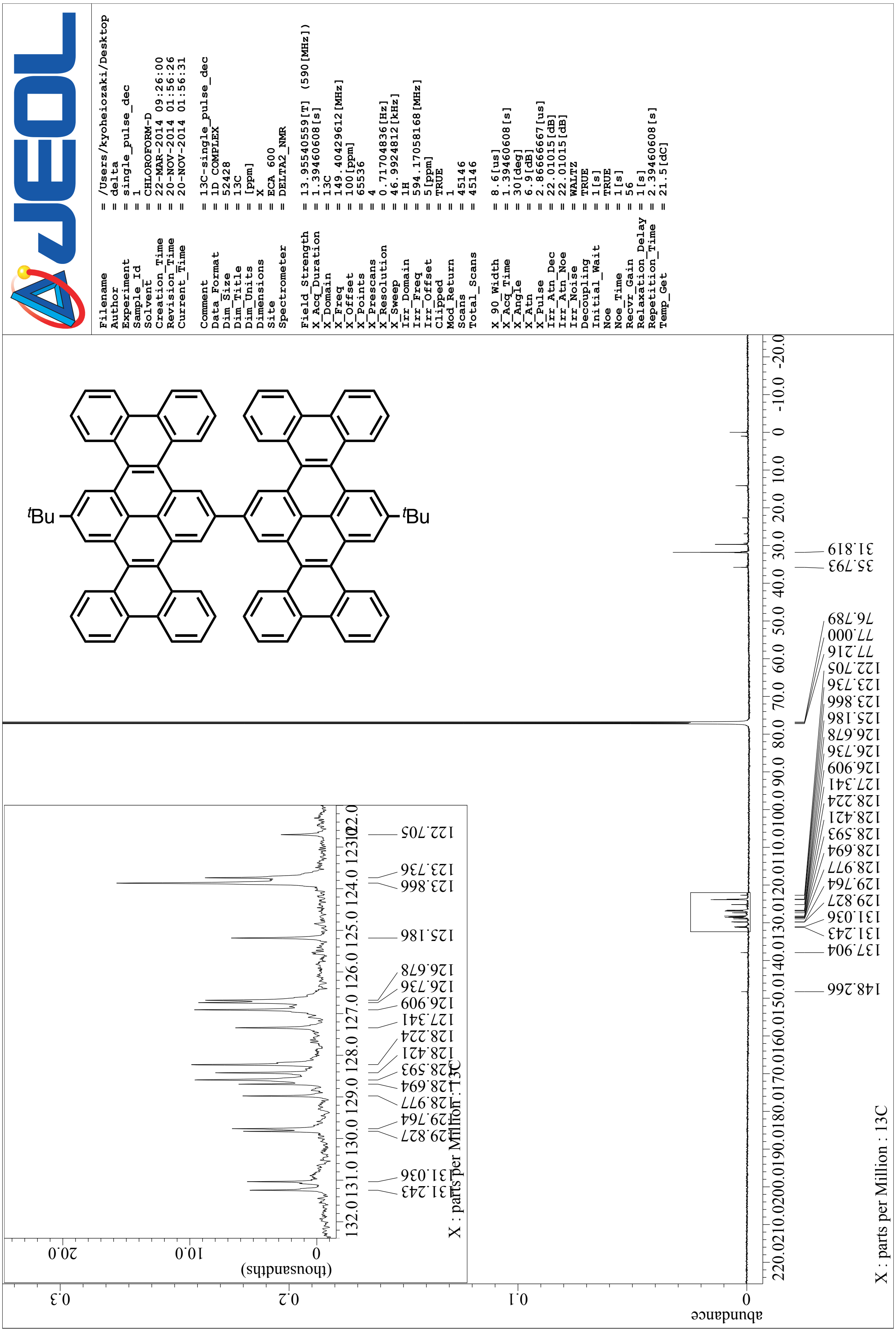

Supplementary Figure 36. ^{13}**C NMR (150 MHz, CDCl$_3$) of 9**

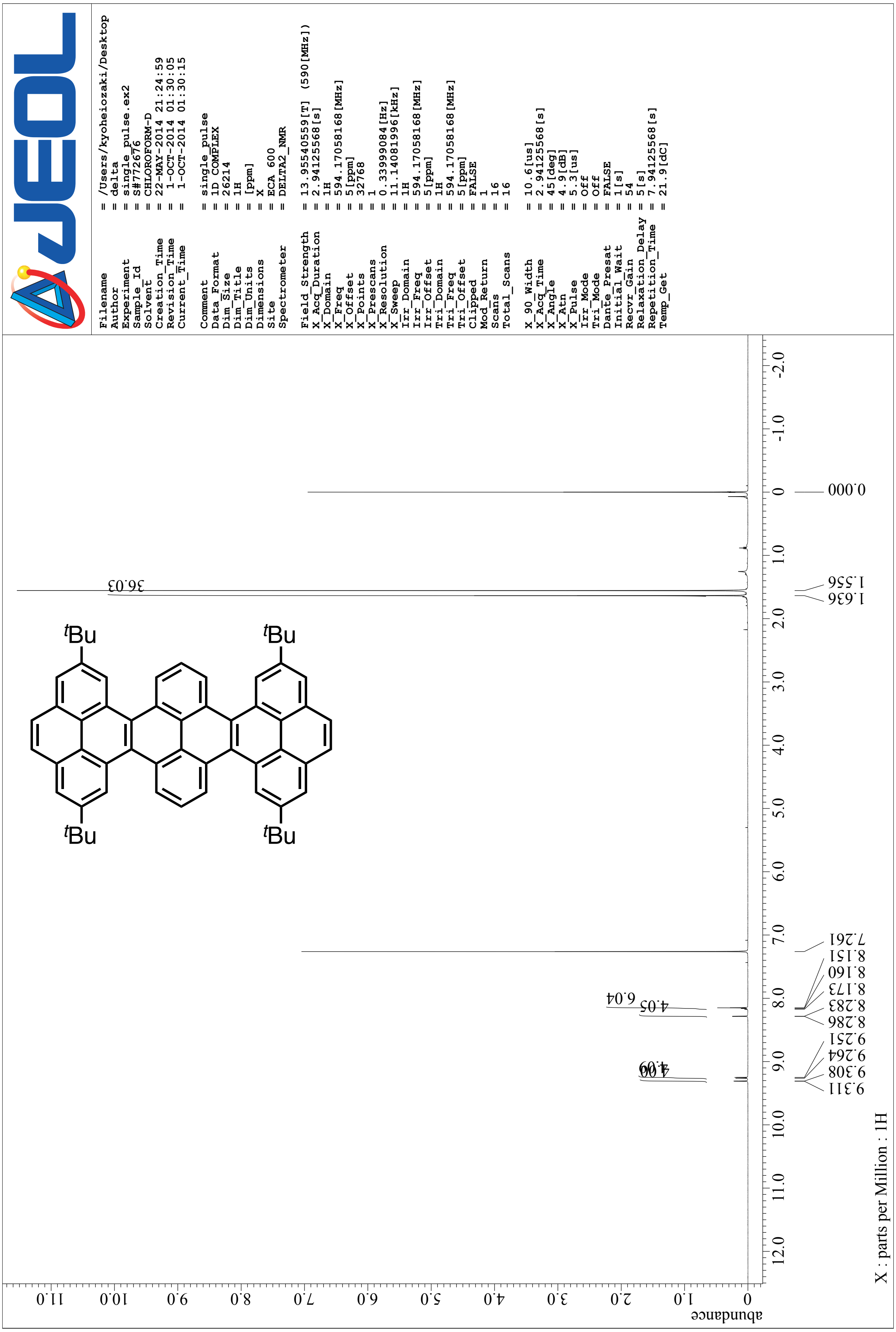

Supplementary Figure 37. ^{1}H NMR (600 MHz, $CDCl_3$) of 11

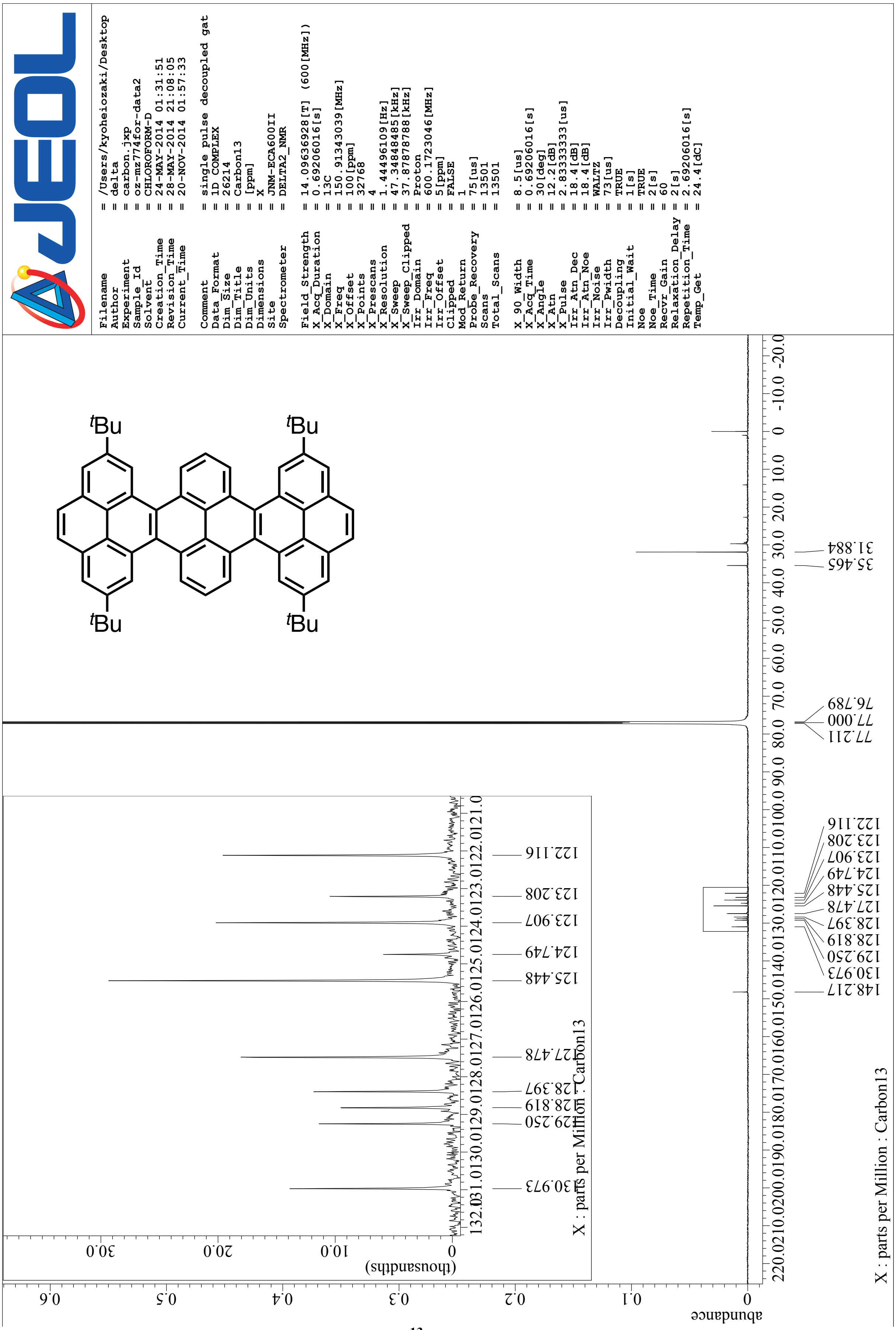

Supplementary Figure 38. ^{13}**C NMR (150 MHz, CDCl$_3$) of 11**

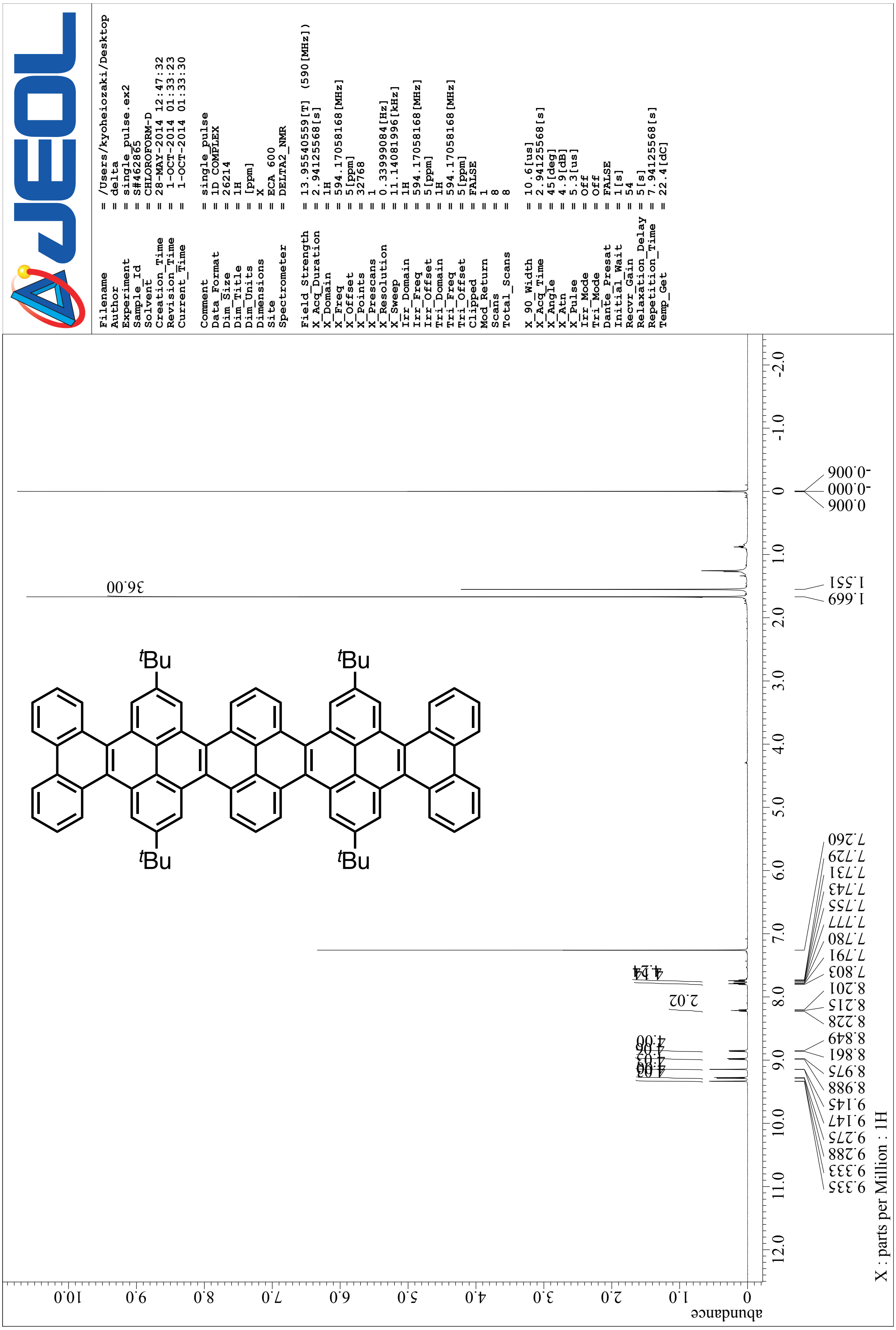

Supplementary Figure 39. **^{1}H NMR (600 MHz, $CDCl_3$) of 12**

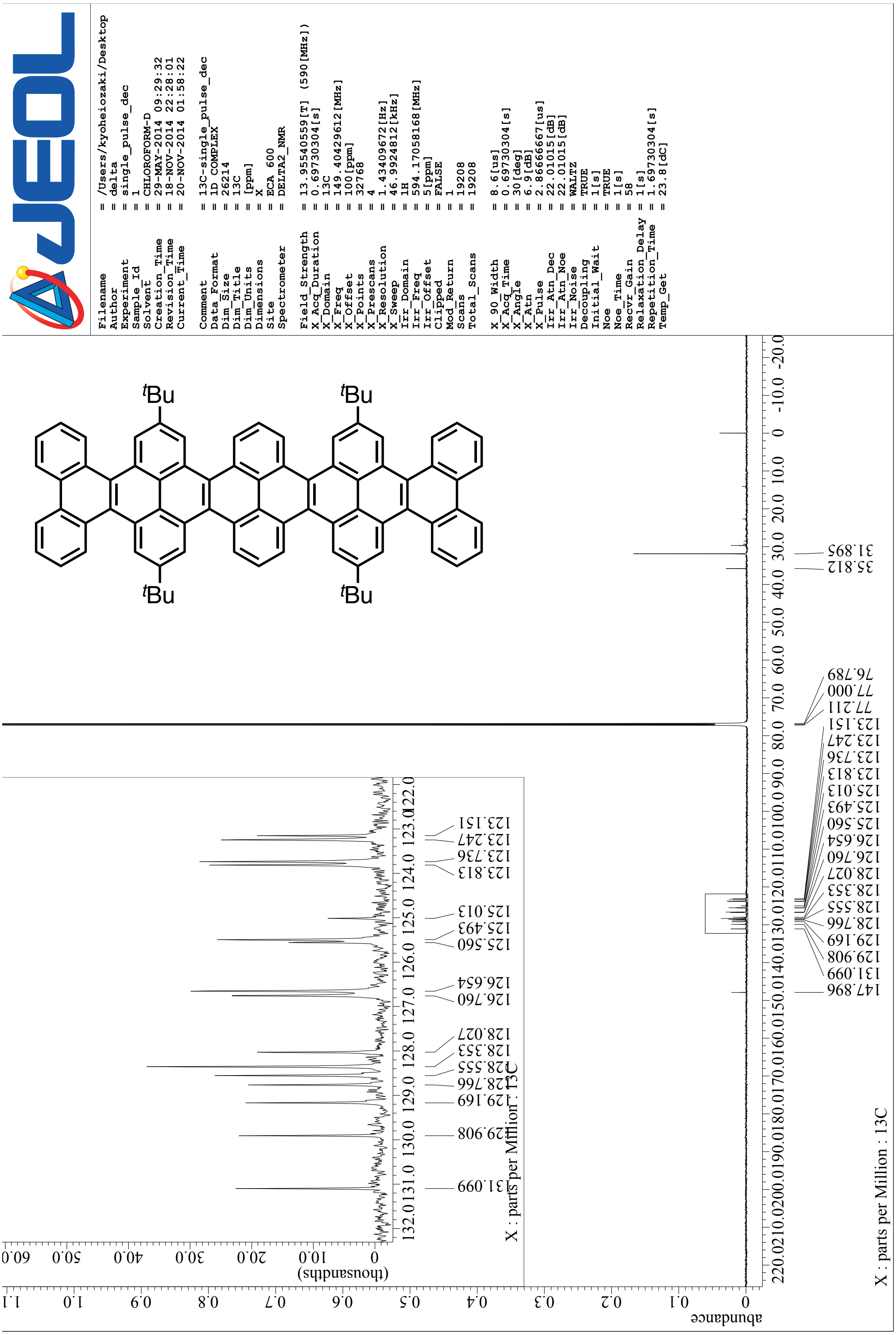

Supplementary Figure 40. ^{13}C NMR (150 MHz, $CDCl_3$) of 12

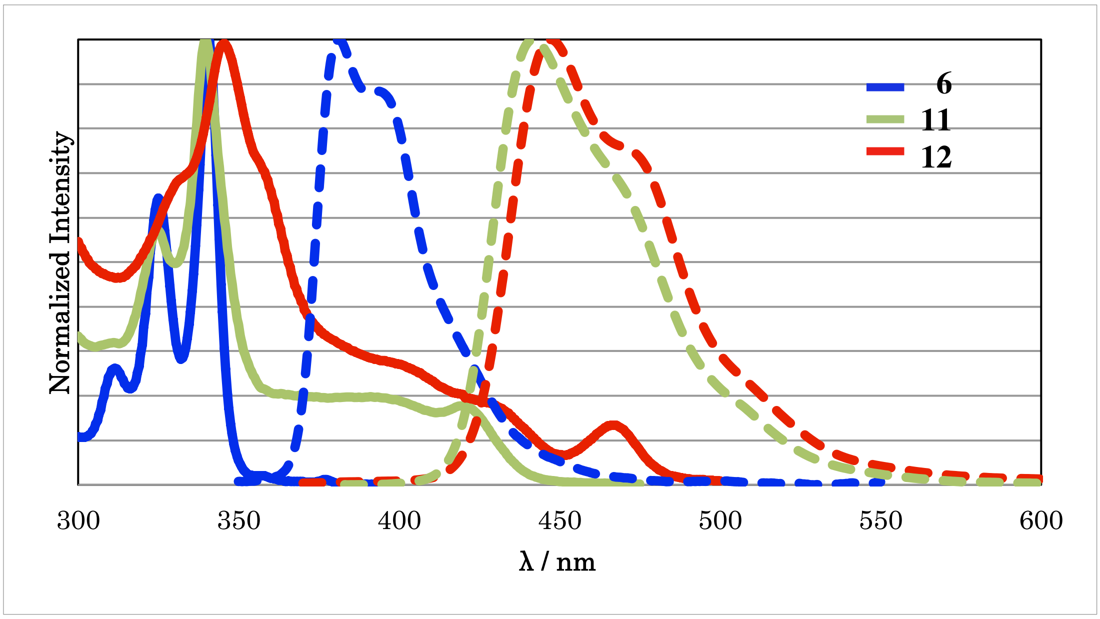

Supplementary Figure 41. UV-vis absorption (solid line) and fluorescence spectra (broken line) of **6**, **11**, and **12** in cyclohexane. Absorption and fluorescence spectra were normalized.

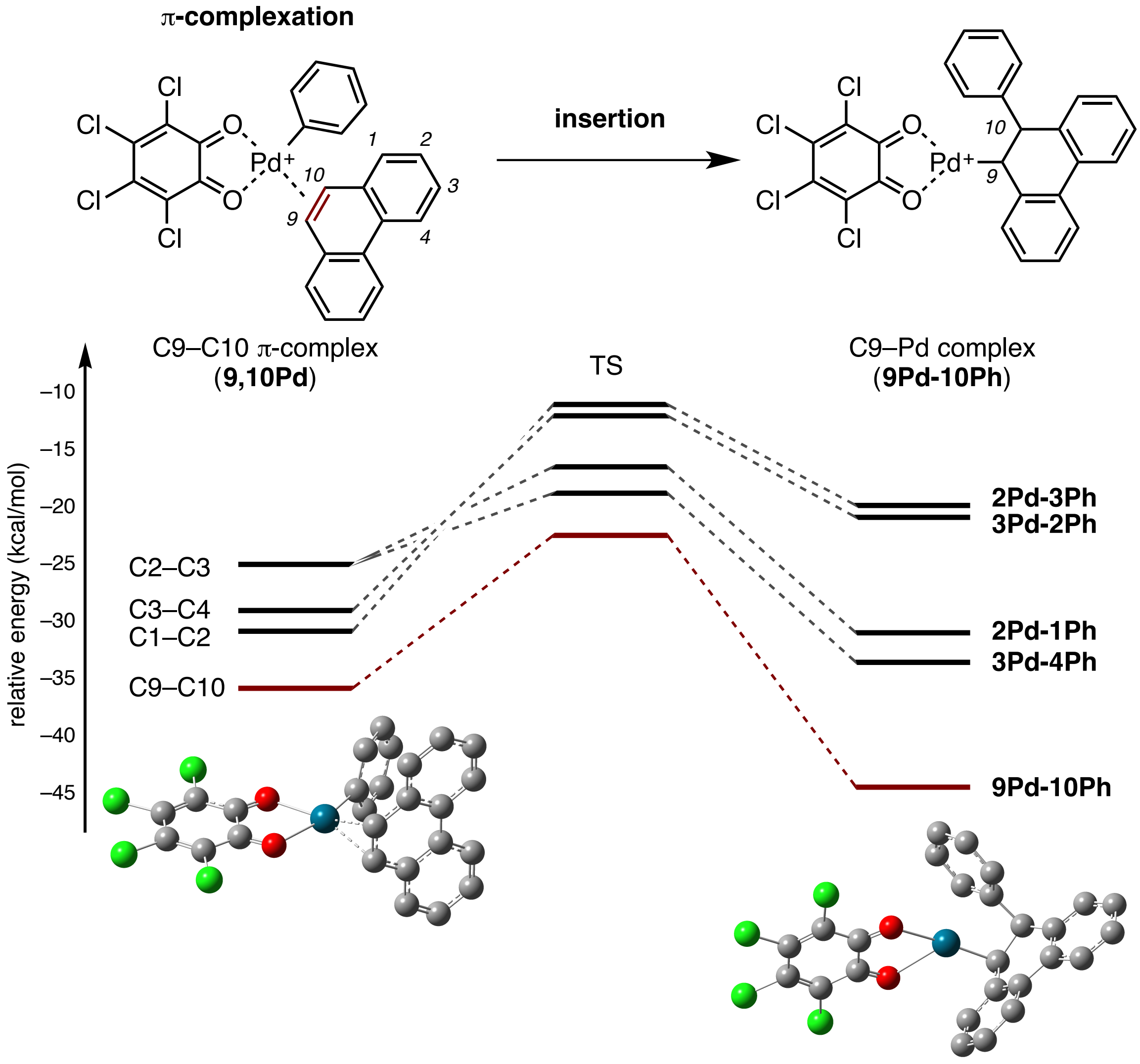

Supplementary Figure 42. Energy surface of π-complexation and insertion

Supplementary Table 1. Uncorrected and zero-point/thermal-corrected (353.15K) energies of stationary points (Hartree) and their Cartesian coordinates.[a]

compound	SCS-MP2	ZPE-corrected	H	G
**9,10Pd**	-3112.28758017	-3111.952546	-3111.911379	-3112.037355
**TS_9Pd-10Ph**	-3112.26739205	-3111.932653	-3111.892403	-3112.015648
**9Pd-10Ph**	-3112.30257031	-3111.965748	-3111.925322	-3112.049677
**1,2Pd**	-3112.27922752	-3111.944296	-3111.903161	-3112.029096
**TS_2Pd-3Ph**	-3112.25061561	-3111.916266	-3111.876115	-3111.998444
**2Pd-3Ph**	-3112.26477025	-3111.928666	-3111.888330	-3112.011000
**2,3Pd_1**	-3112.27230942	-3111.937552	-3111.896336	-3112.022723
**TS_2Pd-1Ph**	-3112.25914851	-3111.924700	-3111.884439	-3112.007596
**2Pd-1Ph**	-3112.28180495	-3111.945207	-3111.904938	-3112.027455
**2,3Pd_2**	-3112.27249533	-3111.937763	-3111.896527	-3112.023023
**TS_3Pd-4Ph**	-3112.26270610	-3111.928156	-3111.887933	-3112.010646
**3Pd-4Ph**	-3112.28587841	-3111.949187	-3111.908972	-3112.031041
**3-4Pd**	-3112.28066663	-3111.945701	-3111.904573	-3112.030501
**TS_3Pd-2Ph**	-3112.25108298	-3111.916719	-3111.876578	-3111.998839
**3Pd-2Ph**	-3112.26532032	-3111.929213	-3111.888909	-3112.011259

(a) E: electronic energy; ZPE: zero-point energy; H ($=E+ZPE+E_{vib}+E_{rot}+E_{trans}+RT$): sum of electronic and themal enthalpies; G ($=H–TS$): sum of electronic and thermal free energies

9,10Pd

O	-1.16586400	1.12955600	0.42833200
O	-1.26581600	-1.15066900	-0.94787600
C	-2.26847900	0.76384300	0.01020200
C	-3.48903000	1.50768100	0.23094600
C	-2.32588500	-0.54633800	-0.78043200
C	-4.66599800	1.01829200	-0.27448900
C	-3.60468600	-1.00147100	-1.28366100
C	-4.72417200	-0.24914100	-1.03932600
Cl	-3.37591400	2.96050900	1.11644400
Cl	-6.11381600	1.87233700	-0.02944200
Cl	-6.23535300	-0.76399800	-1.62032100
Cl	-3.62685900	-2.46587300	-2.15939100
Pd	0.58425000	-0.13359000	-0.00600600
C	1.92831300	-1.95620200	-0.01490400
C	2.14484500	-1.24904000	-1.20763800
C	2.96690900	-2.05302000	0.98095900
H	1.09629700	-2.65692800	0.05433200
C	1.82794500	1.06431600	0.93363700
C	3.41089900	-0.60645400	-1.46128500
H	1.48028700	-1.40625900	-2.05720500
C	2.75164900	-2.80781500	2.15141000
C	4.22201600	-1.42115200	0.75275400
C	2.36382200	2.14000400	0.23568700
C	1.98014000	0.91230100	2.30665600
C	4.44792700	-0.68598500	-0.48923100
C	3.63091900	0.05668400	-2.68510700
C	3.74694000	-2.93360600	3.10259700
H	1.79117300	-3.29725700	2.29454900
C	5.21405300	-1.56343200	1.74415600
C	3.07448000	3.10928700	0.95430500
H	2.24810900	2.23912800	-0.84067500
C	2.69413700	1.89203800	3.00751700
H	1.56660700	0.05855100	2.83759100
C	5.67571900	-0.06319800	-0.79243300
H	2.82991200	0.08799900	-3.41992000
C	4.84858200	0.65297600	-2.95530200
C	4.98255400	-2.29954100	2.89432900
H	3.57841500	-3.52138300	3.99980000
H	6.18406400	-1.09687100	1.61634900
C	3.23829500	2.98573600	2.33404500
H	3.49909600	3.95837200	0.42415800
H	2.82147800	1.78945900	4.08248600
C	5.87217500	0.59406700	-1.99568200
H	6.49364600	-0.09450500	-0.08183200
H	5.01534700	1.15711200	-3.90234600
H	5.76942000	-2.39301500	3.63710200
H	3.79194800	3.74158000	2.88469400
H	6.83157200	1.06027300	-2.20017100

TS_9Pd-10Ph

O	-1.14624800	1.36928500	0.15064300
O	-1.18847000	-1.12728600	-0.75391200
C	-2.23689700	0.80330700	0.11671200
C	-3.48394800	1.42284000	0.52504500
C	-2.25993300	-0.64074700	-0.38768000
C	-4.64122800	0.69328300	0.46885700
C	-3.51970400	-1.35190400	-0.41586700
C	-4.65901700	-0.70858300	-0.00746900
Cl	-3.41481000	3.03881200	1.06664700
Cl	-6.11365000	1.39350500	0.95438300
Cl	-6.15128900	-1.52242900	-0.04186000
Cl	-3.50233800	-2.96485100	-0.97818200
Pd	0.65716500	0.17094600	-0.59880600
C	2.18382300	-1.03121800	-1.33312400
C	3.14785800	0.03285400	-1.33709900
C	2.27975900	-2.08406800	-0.32886500
H	1.72055500	-1.29054300	-2.28947200
C	2.03884300	1.66383100	-0.56373000
C	4.30144000	-0.03454000	-0.45656300
H	3.28816400	0.56411100	-2.27299900
C	1.34657300	-3.14190200	-0.33284100
C	3.32162000	-2.05559200	0.63896200
C	1.84236900	2.63490400	-1.55686700
C	2.39739900	2.03222600	0.74086000
C	4.37637700	-1.03989700	0.54024700
C	5.34568600	0.89557200	-0.61180900
C	1.39931900	-4.13601000	0.62880900
H	0.57793200	-3.16306800	-1.10014300
C	3.33467100	-3.06721400	1.61700200
C	1.96207200	3.98593300	-1.22384500
H	1.59948600	2.34707300	-2.57734000
C	2.50358700	3.38384100	1.06115200
H	2.60284300	1.27464900	1.49346000
C	5.51687900	-1.05484900	1.36828700
H	5.26406300	1.65639800	-1.38407900
C	6.45848300	0.85190000	0.20836600
C	2.39020700	-4.08404000	1.61837800
H	0.67954300	-4.94871600	0.61628700
H	4.09918100	-3.07274000	2.38564200
C	2.28868600	4.35865800	0.08103000
H	1.80721100	4.74350900	-1.98791300
H	2.76713100	3.67696500	2.07420900
C	6.53514200	-0.12771400	1.20943900
H	5.62378900	-1.81172500	2.13693100
H	7.26513500	1.56666500	0.07793800
H	2.43322200	-4.85274500	2.38439800
H	2.39093000	5.41070200	0.33360400
H	7.40403300	-0.17087200	1.85964000

9Pd-10Ph

O	-0.82511600	0.73880900	0.61766600
O	-1.24056000	-0.97207000	-1.38524100
C	-1.98862100	0.43082200	0.35883100
C	-3.13346000	0.97017400	1.06478000
C	-2.22214100	-0.58454200	-0.76303200
C	-4.38930500	0.52393600	0.74724900
C	-3.58413900	-1.02536200	-1.02560900
C	-4.61723300	-0.48671500	-0.30838200
Cl	-2.83112500	2.12737900	2.28550800
Cl	-5.74363900	1.13070600	1.58089900
Cl	-6.21945100	-0.97355900	-0.62063400
Cl	-3.80149800	-2.18981800	-2.25569200
Pd	0.93600300	-0.02782200	-0.54084200
C	2.57988800	-0.87579600	-1.39764000
C	3.58572400	0.25300300	-1.28798300
C	2.46475800	-1.83246300	-0.32106000
H	2.26977400	-1.19118700	-2.39577000
C	2.67441500	1.44405100	-0.87617700
C	4.70174200	-0.01006500	-0.30831800
H	4.02006400	0.48123500	-2.26739000
C	1.38332800	-2.75772600	-0.34897600
C	3.35213500	-1.79614700	0.80083200
C	1.68554300	1.88319600	-1.80470600
C	2.85326300	2.18756700	0.31545100
C	4.56125400	-0.95749200	0.72655000
C	5.87695300	0.73805300	-0.41211800
C	1.13862200	-3.58893900	0.73917200
H	0.77886100	-2.83393900	-1.24931200
C	3.06110300	-2.63287500	1.88060300
C	0.94060800	3.05179500	-1.55210900
H	1.62030900	1.41589700	-2.78592800
C	2.10782200	3.33356800	0.54634800
H	3.60464500	1.87247300	1.03297200
C	5.62557500	-1.12270400	1.63180500
H	5.97727900	1.46234600	-1.21727200
C	6.91778900	0.56829600	0.49714100
C	1.97190400	-3.50836400	1.85510500
H	0.31757700	-4.29828300	0.71241200
H	3.69037400	-2.61936100	2.76378100
C	1.15340600	3.77211700	-0.38691200
H	0.22082000	3.39399000	-2.29088900
H	2.27541000	3.90511700	1.45530200
C	6.78669400	-0.36715700	1.52514200
H	5.56224000	-1.86889600	2.41657000
H	7.82824900	1.15158300	0.39746300
H	1.78916700	-4.14905600	2.71307600
H	0.59197600	4.68314600	-0.19864900
H	7.59557300	-0.51922700	2.23334100

1,2Pd

O	1.69889300	0.50507000	-0.30945400
O	0.96746500	-1.85102300	0.69278900
C	2.53996400	-0.40504800	-0.31209700
C	3.87651900	-0.24447100	-0.83314400
C	2.12236700	-1.75205500	0.26747400
C	4.74158100	-1.30892100	-0.78523200
C	3.08521700	-2.83071600	0.28691300
C	4.34170300	-2.61479900	-0.21927700
Cl	4.29518100	1.27924600	-1.47650000
Cl	6.32236400	-1.14465900	-1.38718200
Cl	5.48866600	-3.86925700	-0.20730400
Cl	2.58085200	-4.32171200	0.94753400
Pd	-0.26671000	0.07839600	0.49870800
C	-2.51980700	0.54943200	2.74043500
C	-1.84977400	-0.50462500	2.06703900
C	-3.60018100	1.16817300	2.15426400
C	-2.29845600	-0.89684800	0.78421200
C	-4.08348000	0.80180100	0.86948900
H	-4.10135100	1.95389700	2.70825700
C	-3.42564100	-0.25438000	0.17841900
H	-1.97537200	-1.84985100	0.36554400
C	-5.23128200	1.44018500	0.25752400
C	-3.91215700	-0.70053400	-1.08235200
C	-5.68513500	0.96936700	-1.01105100
C	-5.93003600	2.51254900	0.85769400
C	-5.00475600	-0.11068300	-1.65165500
H	-3.40805100	-1.52444000	-1.58077900
C	-6.80923600	1.57142000	-1.62148100
C	-7.02594900	3.08631500	0.24048400
H	-5.61306700	2.90679400	1.81686800
H	-5.37824800	-0.46275200	-2.60987500
H	-7.14533500	1.19765100	-2.58514200
C	-7.47283000	2.61421200	-1.00740000
H	-7.54538300	3.90806800	0.72449900
H	-8.33627300	3.06940400	-1.48313100
H	-1.15863600	-1.13776700	2.62075300
H	-2.19690600	0.84131300	3.73478200
C	-0.90875800	1.89729500	0.12084100
C	-1.47458700	2.16458800	-1.11958300
C	-0.61322400	2.89852800	1.03910800
C	-1.75749000	3.49672200	-1.44751600
H	-1.70314000	1.37197300	-1.82746200
C	-0.90378800	4.22385200	0.69309600
H	-0.16258200	2.67565000	2.00282200
C	-1.47536200	4.52117400	-0.54447700
H	-2.20210700	3.72266000	-2.41371400
H	-0.67568200	5.01947300	1.39841700

H	-1.69837300	5.55215500	-0.80576900

TS_2Pd-3Ph

O	-0.64776800	-0.25098400	-2.86963000
O	1.22236800	-2.45802500	-1.49569900
C	0.13715100	-1.20430400	-2.95767200
C	1.38370900	-1.15558000	-3.68792300
C	-0.26270200	-2.49937600	-2.27813700
C	2.09718000	-2.31363800	-3.86925800
C	0.49835500	-3.69426000	-2.55618100
C	1.64679300	-3.59684800	-3.30229800
Cl	1.85374300	0.34873500	-4.34734000
Cl	3.53618000	-2.29434600	-4.78085000
Cl	2.59917100	-4.98059400	-3.58175400
Cl	-0.04655500	-5.15804100	-1.86043800
Pd	-2.38396200	-0.51474200	-1.47442800
C	-4.63870000	0.53213800	-0.42759700
C	-4.03157100	-0.78192900	-0.22204900
C	-5.73395900	0.64482900	-1.32573200
C	-4.41860900	-1.82945800	-1.09740200
C	-6.11142300	-0.38553300	-2.17548200
H	-6.23874900	1.60372200	-1.36560000
C	-5.40903200	-1.64644800	-2.08145300
H	-4.01684200	-2.82672600	-0.93958200
C	-7.19740800	-0.25154300	-3.14667300
C	-5.77119500	-2.72566200	-2.94318000
C	-7.51713300	-1.36252600	-3.97728000
C	-7.94583800	0.93104100	-3.30166200
C	-6.78047100	-2.58678200	-3.84788700
H	-5.23376400	-3.66572900	-2.85573400
C	-8.56048200	-1.25611900	-4.92075100
C	-8.96856100	1.01526100	-4.23388400
H	-7.73770900	1.80015400	-2.68665600
H	-7.05152200	-3.41969400	-4.49162500
H	-8.79330100	-2.11463900	-5.54517700
C	-9.28031500	-0.08269600	-5.05065000
H	-9.53239900	1.93849200	-4.32905300
H	-10.08314700	-0.01040500	-5.77800900
H	-3.57674400	-1.01536100	0.74041200
H	-4.61367100	1.23283800	0.40064300
C	-3.07130800	1.41189000	-1.38797900
C	-3.33737800	1.97154200	-2.65106900
C	-2.25846700	2.08826300	-0.45985500
C	-2.74257600	3.17900700	-2.99495800
H	-3.99460500	1.46169200	-3.35081800
C	-1.66956300	3.30311900	-0.82338400
H	-2.09742500	1.68387500	0.53671400
C	-1.91152800	3.84628200	-2.08349600
H	2.92992500	3.60877500	-3.97569100
H	-1.03415900	3.82469400	-0.11234100
H	-1.46553200	4.79846800	-2.35760600

2Pd-3Ph

O	1.51798300	0.83758900	-0.31378100
O	0.93359700	-1.31012900	1.14305500
C	2.30110100	-0.12838000	-0.35812800
C	3.56299200	-0.11192500	-1.05500800
C	1.86743900	-1.39609200	0.32306500
C	4.23419000	-1.29596700	-1.24847900
C	2.56161800	-2.62010500	0.01797000
C	3.72479400	-2.56316500	-0.71355700
Cl	4.09960500	1.38210900	-1.69298100
Cl	5.68494700	-1.31755400	-2.14627300
Cl	4.62137300	-3.98565400	-1.00077100
Cl	1.94808900	-4.07124000	0.68829700
Pd	-0.18544800	0.63790400	1.09828300
C	-2.45873700	1.73354200	2.09138600
C	-1.82827400	0.37710800	2.33812900
C	-3.62245100	1.71225200	1.16657500
C	-2.20492900	-0.70664900	1.51080500
C	-3.97515000	0.64354900	0.39805300
H	-4.17143700	2.64613400	1.08895900
C	-3.19360000	-0.58887800	0.51819500
H	-1.77533900	-1.68693800	1.70008000
C	-5.10131800	0.67924800	-0.55270800
C	-3.49830800	-1.69386600	-0.32182600
C	-5.34880000	-0.46248100	-1.36295600
C	-5.94454300	1.78991200	-0.70069800
C	-4.52351500	-1.62807400	-1.22274300
H	-2.90696900	-2.60007600	-0.22793900
C	-6.41016600	-0.45379000	-2.29001300
C	-6.98957500	1.78123500	-1.61799100
H	-5.80467400	2.67681300	-0.09138900
H	-4.74247600	-2.48745600	-1.85179800
H	-6.58326500	-1.33625300	-2.90025800
C	-7.22517800	0.65792100	-2.42076100
H	-7.62904000	2.65445100	-1.70698800
H	-8.04273800	0.65798100	-3.13487400
H	-1.41369100	0.16826200	3.32367500
H	-2.75750500	2.22000700	3.02912400
C	-1.26785300	2.56045400	1.48381300
C	-1.34815700	3.23650100	0.23871300
C	-0.13055000	2.79877700	2.30740400
C	-0.34361400	4.10691900	-0.15448100
H	-2.20861600	3.08044300	-0.40551300
C	0.87192300	3.69103100	1.89500100
H	-0.09250100	2.38548400	3.31278400
C	0.76454400	4.34303700	0.67419000
H	-0.42348500	4.61901700	-1.10947300
H	1.71912100	3.87911100	2.54864800
H	1.53404200	5.04324600	0.36156400

2,3Pd_1

O	-1.46856800	1.00530700	0.38066500
O	-1.57184600	-1.27980300	-0.97716700
C	-2.57674500	0.45946300	0.25887600
C	-3.79079800	1.00786500	0.81199600
C	-2.63294000	-0.85222800	-0.50958900
C	-4.96898900	0.32508500	0.63743500
C	-3.90567400	-1.52281200	-0.65008400
C	-5.02640200	-0.95299900	-0.10068200
Cl	-3.67717000	2.48470200	1.65943900
Cl	-6.41374500	0.94779300	1.28140100
Cl	-6.53478500	-1.72112300	-0.26098800
Cl	-3.92619800	-2.99685700	-1.51116700
Pd	0.23685200	0.05543200	-0.51780700
C	2.80657300	-0.13432600	-2.23177500
C	1.68985900	-0.93495400	-1.93450600
C	3.96036700	-0.16127800	-1.43757600
H	2.79256400	0.49453700	-3.11795000
C	1.74765400	-1.78919800	-0.78998500
C	5.09242000	0.64598400	-1.78193100
C	4.01507500	-1.01870300	-0.28583500
C	2.89056900	-1.80825500	0.01031600
H	0.97042000	-2.53198500	-0.62655500
C	6.22641500	0.60680600	-1.03193800
H	5.02824100	1.28717200	-2.65648200
C	5.22861300	-1.05104500	0.51366100
H	2.90790300	-2.48705100	0.85554100
C	6.32998200	-0.23212600	0.12563900
H	7.08279300	1.21859500	-1.30305400
C	5.37263000	-1.86217000	1.66056400
C	7.51981500	-0.25501700	0.88553500
C	6.54768800	-1.86825300	2.39003300
H	4.55928900	-2.50060100	1.98848800
H	8.35160500	0.37201400	0.57567700
C	7.63131100	-1.05987900	2.00209700
H	6.63348300	-2.50380800	3.26640900
H	8.55150600	-1.07135400	2.57847700
H	0.92933000	-1.08916700	-2.69929600
C	1.43053300	1.50392700	0.06707700
C	2.10547100	1.38604700	1.27492200
C	1.42101800	2.68017600	-0.67434300
C	2.80201700	2.50184700	1.75653400
H	2.09288600	0.46358900	1.84891600
C	2.12461100	3.78390800	-0.17680300
H	0.88538200	2.75832600	-1.61728100
C	2.81360700	3.69392400	1.03285100
H	3.32832000	2.42983300	2.70529500
H	2.12910500	4.71115100	-0.74477600
H	3.35681400	4.55478200	1.41329900

TS_2Pd-1Ph

O	-1.48557900	1.14979800	0.27640600
O	-1.56477800	-1.11452600	-1.11761600
C	-2.55179000	0.53410200	0.25714300
C	-3.76447700	0.99746500	0.90253000
C	-2.57609500	-0.80068800	-0.48443500
C	-4.87193000	0.19108700	0.89993200
C	-3.77042600	-1.61402100	-0.42033300
C	-4.87363800	-1.12961700	0.23416400
Cl	-3.71266500	2.52207400	1.66735600
Cl	-6.29562600	0.69717700	1.68285500
Cl	-6.30078700	-2.05308300	0.28958800
Cl	-3.73671600	-3.12681100	-1.21282500
Pd	0.21810600	0.26681000	-0.95459100
C	2.72794600	0.34218300	-1.82311100
C	1.70041600	-0.64005900	-2.09632100
C	3.81520700	0.01519900	-0.93152100
H	2.95184700	1.05916500	-2.60650700
C	1.71141300	-1.85547000	-1.32941200
C	4.91258400	0.90898000	-0.80628900
C	3.77685700	-1.18344100	-0.18039300
C	2.68789500	-2.08461300	-0.38996800
H	0.95824400	-2.61125800	-1.52973800
C	5.94510200	0.62521000	0.04316900
H	4.92069300	1.82094700	-1.39713600
C	4.85755100	-1.48034100	0.73628100
H	2.64893700	-3.01286600	0.16878400
C	5.94620800	-0.56041200	0.83625400
H	6.78782900	1.30658900	0.12579200
C	4.89282100	-2.64279100	1.54259400
C	7.01691000	-0.83443400	1.71974100
C	5.94866200	-2.88575100	2.39901600
H	4.08572000	-3.36594100	1.50128200
H	7.83938400	-0.12657800	1.78035800
C	7.02166900	-1.97758900	2.49003500
H	5.95145800	-3.78630500	3.00600200
H	7.84809000	-2.18005000	3.16464600
H	1.22419300	-0.63313000	-3.07874400
C	1.58026600	1.77142300	-0.80525900
C	1.99772500	2.03017200	0.50923500
C	1.23592600	2.82279800	-1.67114000
C	2.01191000	3.34358500	0.96994600
H	2.31038700	1.21746700	1.16008200
C	1.25818500	4.13593700	-1.19425000
H	0.96081500	2.62542300	-2.70479700
C	1.64339000	4.39451000	0.12121700

H	2.31999700	3.55044000	1.99170500
H	0.98604900	4.95300400	-1.85750000
H	1.67314200	5.41805500	0.48511700

2Pd-1Ph

O	-1.39063100	1.29368000	0.36852800
O	-1.37342800	-0.90912700	-1.11970300
C	-2.43740300	0.64157900	0.30088200
C	-3.68719100	1.04992200	0.90616400
C	-2.38690700	-0.67850300	-0.44491400
C	-4.74286000	0.17440000	0.91514200
C	-3.51192400	-1.57779900	-0.35153800
C	-4.65249500	-1.15528600	0.28489800
Cl	-3.73950400	2.58996200	1.64390000
Cl	-6.20279600	0.60698700	1.67903200
Cl	-6.01669900	-2.17207200	0.34893400
Cl	-3.37370500	-3.10105900	-1.11547300
Pd	0.34292400	0.51244000	-0.90388600
C	2.94644600	0.60827000	-1.69173700
C	1.82673100	-0.36444900	-2.02653500
C	4.04497300	0.05976500	-0.81804200
H	3.39479600	1.01590600	-2.60555300
C	1.78353000	-1.61831500	-1.33228100
C	5.21532800	0.82493800	-0.66280500
C	3.89825800	-1.15862900	-0.13614500
C	2.72840900	-1.95324500	-0.39280900
H	0.99936800	-2.32679500	-1.58307000
C	6.22913600	0.39832800	0.16720300
H	5.31946200	1.75916100	-1.20906700
C	4.94332900	-1.61398400	0.75301500
H	2.61553100	-2.90351500	0.11743800
C	6.12117500	-0.81291000	0.89503700
H	7.13364600	0.99249300	0.26974200
C	4.86914000	-2.81651000	1.49682900
C	7.16556900	-1.23760400	1.75529700
C	5.89957300	-3.20464400	2.32966600
H	3.99582700	-3.45559100	1.42783500
H	8.05312400	-0.61689000	1.84545800
C	7.05961200	-2.41342500	2.46245600
H	5.81502800	-4.13196100	2.88874600
H	7.86243100	-2.73383400	3.11937600
H	1.42611400	-0.33488800	-3.04143900
C	2.15796600	1.76755700	-0.99683100
C	2.37312100	2.16541700	0.34963600
C	1.28343700	2.55714500	-1.80053300
C	1.76409000	3.30644200	0.85048200
H	3.04203200	1.58629300	0.97863600
C	0.68484200	3.71647700	-1.27633700
H	1.18704200	2.34290500	-2.86290700
C	0.92667700	4.08858900	0.03847100
H	1.94880600	3.60267500	1.87947700
H	0.05117900	4.32508600	-1.91540300
H	0.47387400	4.99070400	0.44033700

2,3Pd_2

O	-1.42463200	1.07619600	0.21561800
O	-1.66370500	-1.50272000	-0.39052800
C	-2.57111500	0.62379800	0.07723000
C	-3.76032800	1.42846400	0.22139300
C	-2.70710700	-0.85518000	-0.25737800
C	-4.98789800	0.83422200	0.06703400
C	-4.03115700	-1.41779300	-0.40101200
C	-5.12420300	-0.60323800	-0.24692800
Cl	-3.55580900	3.08276600	0.58617200
Cl	-6.40488800	1.75740000	0.23891600
Cl	-6.69014700	-1.24268000	-0.41818800
Cl	-4.14234200	-3.08169300	-0.76620800
Pd	0.24137100	-0.26137200	-0.06468800
C	2.95508200	-1.22328600	-1.15954300
C	1.70766300	-1.78358400	-0.81277200
C	3.99946500	-1.10868900	-0.23685600
H	3.09984600	-0.91315300	-2.18842800
C	1.50099800	-2.25725000	0.52060200
C	3.77360800	-1.58443300	1.10006400
C	5.29923100	-0.55193500	-0.58609000
C	2.52609400	-2.13628400	1.45157100
H	0.60620000	-2.82369000	0.76657900
C	4.82724500	-1.51614400	2.06339400
C	6.31736600	-0.51246200	0.41025100
C	5.60449700	-0.05090800	-1.86851800
H	2.38425000	-2.51122900	2.46172200
C	6.04489100	-1.00607700	1.72677200
H	4.64218700	-1.88735600	3.06756400
C	7.58969000	0.01251900	0.09381600
C	6.85792100	0.45947600	-2.15722100
H	4.85670600	-0.05644300	-2.65426800
H	6.84151700	-0.96740300	2.46548300
H	8.35573600	0.03151800	0.86452600
C	7.86071100	0.49232800	-1.17214000
H	7.06728300	0.83668500	-3.15389800
H	8.84209900	0.89255600	-1.40801200
H	1.04440000	-2.10575900	-1.61557100
C	1.54180600	1.18176700	0.23744200
C	2.04267600	1.38426100	1.51679800
C	1.77993700	2.06668100	-0.80846500
C	2.81472000	2.52872800	1.75350600
H	1.83858500	0.68853600	2.32636200
C	2.55596700	3.20380500	-0.55232400
H	1.37973200	1.89606100	-1.80490300
C	3.07236700	3.43255100	0.72309100
H	3.20507000	2.70874200	2.75230200
H	2.75387600	3.90504400	-1.35945000
H	3.67368900	4.31720200	0.91452800

TS_3Pd-4Ph

O	-1.38083700	1.10179200	0.11692000
O	-1.72858000	-1.40282400	-0.70688000
C	-2.52986900	0.66272600	0.11567700
C	-3.69400000	1.44088700	0.49435100
C	-2.72253800	-0.79497000	-0.30460600
C	-4.92077600	0.83310500	0.52857700
C	-4.04448200	-1.37784400	-0.22199100
C	-5.09721700	-0.59173100	0.16878400
Cl	-3.44216600	3.07665800	0.90920400
Cl	-6.29613800	1.71738600	0.99905300
Cl	-6.66392100	-1.24835200	0.24471000
Cl	-4.20858400	-3.02148200	-0.65573000
Pd	0.25291600	-0.30563100	-0.65438400
C	2.81587900	-0.83876000	-1.14941300
C	1.65051400	-1.68059500	-1.34551900
C	3.72434800	-1.10094700	-0.05958000
H	3.23570600	-0.39595500	-2.04495800
C	1.34518800	-2.68033500	-0.35753400
C	3.36975000	-2.08843400	0.88910500
C	4.96620000	-0.38499800	0.08071200
C	2.16323400	-2.83972600	0.73067700
H	0.48561300	-3.32636800	-0.50643400
C	4.24360000	-2.37233500	1.98055600
C	5.82581700	-0.70780200	1.17589700
C	5.37786400	0.63290400	-0.81369000
H	1.92648400	-3.59317900	1.47761500
C	5.42893000	-1.70820100	2.11308100
H	3.95523700	-3.13582300	2.69773300
C	7.05877500	-0.02991900	1.32064600
C	6.58545700	1.28082600	-0.64790000
H	4.74114600	0.92536900	-1.64204500
H	6.09434900	-1.93698700	2.94150400
H	7.70585400	-0.29256000	2.15342300
C	7.43738400	0.94658000	0.42379100
H	6.88065400	2.05581700	-1.34922900
H	8.38727000	1.45934800	0.54173900
H	1.29388600	-1.82338000	-2.36783000
C	1.84067200	0.96334300	-0.69490600
C	2.17682800	1.50012600	0.55680900
C	1.76267600	1.78548300	-1.83211500
C	2.37017900	2.87388800	0.67667500
H	2.28871400	0.85280100	1.42299800
C	1.96167200	3.16166800	-1.69565700
H	1.55182000	1.36203300	-2.81173700
C	2.26074400	3.70339900	-0.44516800
H	2.61753700	3.29807800	1.64645700
H	1.89228900	3.80393100	-2.56998300
H	2.42740800	4.77255700	-0.34446400

3Pd-4Ph

O	-1.26965100	1.34727900	0.15154700
O	-1.50738500	-1.15403400	-0.71529100
C	-2.40041900	0.85635000	0.13551700
C	-3.60535300	1.58198500	0.48326100
C	-2.51882000	-0.60807700	-0.25696500
C	-4.79257300	0.90270700	0.57237600
C	-3.78972700	-1.27545300	-0.09735100
C	-4.88487500	-0.54205900	0.28395400
Cl	-3.44896100	3.24777100	0.82621900
Cl	-6.21117500	1.72279800	1.03599600
Cl	-6.40491400	-1.29472700	0.42380200
Cl	-3.85809100	-2.94407200	-0.46086600
Pd	0.41246400	-0.00117200	-0.67218600
C	3.04632400	-0.52808300	-1.11644000
C	1.79368800	-1.36415200	-1.35221600
C	3.95431700	-1.00212800	-0.00898800
H	3.62170500	-0.45348100	-2.04646900
C	1.46689800	-2.41480300	-0.42930100
C	3.52693500	-2.00996400	0.86535700
C	5.23360200	-0.40320100	0.17097300
C	2.26572100	-2.67060700	0.65280000
H	0.58856600	-3.02459700	-0.61926800
C	4.36145500	-2.42690500	1.94337200
C	6.06143300	-0.83959800	1.25992700
C	5.73336400	0.62109700	-0.68243700
H	1.98172400	-3.45405500	1.35164700
C	5.58930900	-1.85679800	2.13475100
H	4.00796900	-3.21093700	2.60752300
C	7.33587000	-0.25238100	1.44285600
C	6.97718000	1.17447400	-0.47512900
H	5.13444600	0.97726400	-1.51433600
H	6.22368600	-2.17801100	2.95639200
H	7.95657000	-0.59608500	2.26607300
C	7.78794700	0.73539500	0.59495400
H	7.34036400	1.95155300	-1.14122300
H	8.76905600	1.17615600	0.74470400
H	1.49305400	-1.50486800	-2.39263400
C	2.42353400	0.87725400	-0.82785200
C	2.57853700	1.55958000	0.40843500
C	1.78589100	1.56711000	-1.90172900
C	2.14219500	2.86929700	0.54811200
H	3.07039200	1.05874500	1.23652000
C	1.36276900	2.89837500	-1.74322500

H	1.74868900	1.11092000	-2.88887900
C	1.54275300	3.54389100	-0.52722600
H	2.28074100	3.38011300	1.49703800
H	0.91319500	3.41920800	-2.58405800
H	1.22581400	4.57621800	-0.40794900

3,4Pd

O	-1.27399200	-1.06967000	0.42837700
O	-1.08925800	0.90227900	-1.35147100
C	-2.31543700	-0.47225800	0.12613800
C	-3.60513600	-0.79518600	0.69017800
C	-2.20914500	0.65712500	-0.89497100
C	-4.70392300	-0.07666200	0.29126300
C	-3.40909800	1.37227800	-1.27006500
C	-4.60458300	1.01783200	-0.69893400
Cl	-3.67330300	-2.06457200	1.82760400
Cl	-6.23310700	-0.43631300	0.93901600
Cl	-6.02677300	1.84475900	-1.12505500
Cl	-3.24923000	2.62763700	-2.41552900
Pd	0.57760700	-0.45974800	-0.53141500
C	2.05083100	-0.06345500	-2.24564800
C	2.96810100	-1.09745300	-2.57367000
C	2.34183100	0.82963900	-1.18503700
C	4.14145400	-1.20353500	-1.86953800
C	3.55720500	0.71388400	-0.43779400
H	1.77364500	1.75472100	-1.12090300
C	4.46398800	-0.31865400	-0.80318300
H	4.86087700	-1.97542300	-2.13040500
C	3.90250900	1.62757000	0.62615200
C	5.70847600	-0.44602400	-0.11661000
C	5.16142100	1.47214000	1.28054100
C	3.05043700	2.67273400	1.05728700
C	6.04294000	0.41953100	0.88252500
H	6.38766000	-1.24184800	-0.40889000
C	5.52478800	2.36351600	2.31588400
C	3.42875100	3.53106700	2.07149800
H	2.07909700	2.81423600	0.59437500
H	6.99601400	0.32088100	1.39557700
H	6.48908400	2.23689300	2.80092100
C	4.67618800	3.37940400	2.70673200
H	2.75916400	4.32840600	2.38047900
H	4.96700400	4.05981200	3.50141800
H	1.25712300	0.18227300	-2.94952400
H	2.75269900	-1.76737400	-3.40001600
C	1.62642500	-1.80633400	0.44282500
C	1.67613100	-3.09953900	-0.06451100
C	2.12973100	-1.47303400	1.69431300
C	2.26005500	-4.09896900	0.72351900
H	1.27139000	-3.34649100	-1.04246700
C	2.70885200	-2.48732700	2.46759100
H	2.08787000	-0.45643700	2.07715200
C	2.77549400	-3.79353100	1.98350900
H	2.30387200	-5.11703800	0.34391400
H	3.10839300	-2.24348300	3.44900700
H	3.22711000	-4.57477000	2.58901800

TS_3Pd-2Ph

O	-1.10448600	-1.36787200	0.42608700
O	-0.88060100	0.54924000	-1.40752700
C	-2.09452400	-0.65766500	0.21444700
C	-3.38261500	-0.86451900	0.83930800
C	-1.92206500	0.51058300	-0.73888500
C	-4.36701600	0.07517600	0.66698200
C	-2.97674600	1.49223000	-0.83299000
C	-4.15985600	1.26827400	-0.17385200
Cl	-3.56890400	-2.24930200	1.82147500
Cl	-5.87110100	-0.11324900	1.44378500
Cl	-5.44187700	2.37801600	-0.32734200
Cl	-2.70584400	2.84948100	-1.83782000
Pd	0.70326200	-0.97612300	-0.85574800
C	2.40099800	-0.55936300	-1.99153400
C	3.26861600	-1.59143200	-1.42373100
C	2.39939200	0.72915800	-1.39010500
C	4.22673000	-1.19271500	-0.45671700
C	3.24529000	1.05556400	-0.31533000
H	1.79799200	1.50023100	-1.85876400
C	4.21326600	0.07261200	0.11736000
H	4.96014300	-1.92085100	-0.12025700
C	3.23046700	2.36168300	0.31783500
C	5.17762600	0.42584100	1.12516600
C	4.21478600	2.66045200	1.30450800
C	2.28023700	3.35628200	-0.00292600
C	5.18036400	1.66307400	1.68131000
H	5.90813400	-0.31998000	1.42587500
C	4.22998600	3.93260700	1.90952100
C	2.31402000	4.59921000	0.60393800
H	1.49963600	3.15412100	-0.72873700
H	5.91781800	1.92192800	2.43648400
H	4.98909700	4.15023600	2.65610900
C	3.29680400	4.89388100	1.56388200
H	1.57481600	5.34898100	0.33807500
H	3.32009600	5.87149100	2.03598900
H	2.04635900	-0.67120400	-3.01620200
H	3.52397800	-2.43807200	-2.05270000
C	1.84498900	-2.61295300	-0.39917700
C	1.35172900	-3.67133000	-1.18467500
C	2.05501800	-2.78115200	0.98182600
C	1.01951900	-4.88242700	-0.57005900
H	1.24310300	-3.56122000	-2.26108300
C	1.71854600	-3.99146900	1.57483600
H	2.47058000	-1.97217200	1.57708800
C	1.20360900	-5.04218900	0.80175400
H	0.63121300	-5.69996800	-1.17187900
H	1.86324900	-4.12317500	2.64409500
H	0.96075300	-5.99018400	1.27400000

3Pd-2Ph

O	-0.98775100	-1.39428000	0.54612000
O	-0.73080800	0.43751500	-1.36624200
C	-1.97642400	-0.68868700	0.27828200
C	-3.28335400	-0.87936100	0.85748700
C	-1.76640600	0.45587500	-0.67362800
C	-4.23893800	0.09338500	0.68430400
C	-2.77066500	1.48402200	-0.75312800
C	-3.97761400	1.29009000	-0.12293000
Cl	-3.53066100	-2.27212000	1.81897900
Cl	-5.76398400	-0.06515500	1.43233900
Cl	-5.22004900	2.44688200	-0.28661200
Cl	-2.43677700	2.84904800	-1.73308100
Pd	0.80303200	-1.07794500	-0.73287400
C	2.49227500	-0.67716800	-1.86522700
C	3.39401200	-1.71563900	-1.22650700
C	2.46767700	0.64091300	-1.34314000
C	4.37619700	-1.14475500	-0.27306500
C	3.28384500	1.05091400	-0.27661200
H	1.85195800	1.36977800	-1.85872900
C	4.32192100	0.13578600	0.19802600
H	5.15503500	-1.81028600	0.09428300
C	3.20424100	2.37536700	0.29872200
C	5.29633200	0.60948100	1.15766500
C	4.19649400	2.78515800	1.23981300
C	2.17748700	3.28712100	-0.03818400
C	5.23962200	1.87072300	1.64037100
H	6.07617600	-0.07578000	1.47782200
C	4.14502200	4.08221500	1.77677500
C	2.14569400	4.55898400	0.50517800
H	1.38729200	2.99099100	-0.71977700
H	5.97904000	2.21923200	2.35611000
H	4.90811900	4.38934500	2.48668700
C	3.13782800	4.96314800	1.41296900
H	1.34848200	5.24354600	0.23223500
H	3.11181300	5.96290100	1.83624400
H	2.18734200	-0.82752600	-2.90038900
H	3.92422800	-2.31406300	-1.97859600
C	2.36724900	-2.66098700	-0.50278400
C	1.44628800	-3.38869800	-1.30992200
C	2.43941700	-2.97013800	0.88013200
C	0.64459100	-4.39308900	-0.74367500
H	1.44958900	-3.25771800	-2.38972500
C	1.63292800	-3.95882100	1.42132000
H	3.14188600	-2.43815400	1.51524000
C	0.73941700	-4.67785200	0.61143100
H	-0.03253600	-4.95588700	-1.38015100
H	1.70471200	-4.18651300	2.48134700
H	0.12889100	-5.46420600	1.04628400

Supplementary Table 2. Effect of catalyst [a]

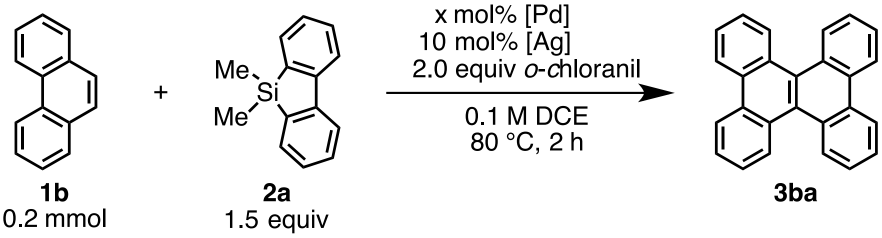

entry	[Pd]	x	[Ag]	yield [b]
1	$Pd(OAc)_2$	5 mol%	none	nd
2	$Pd(OAc)_2$	5 mol%	AgOTf	12%
3	$Pd(OCOCF_3)_2$	5 mol%	AgOTf	23%
4	$PdCl_2$	5 mol%	AgOTf	36%
5	$PdCl_2$	5 mol%	$AgBF_4$	37%
6	$PdCl_2$	5 mol%	$AgPF_6$	2%
7	$PdCl_2$	5 mol%	$AgSbF_6$	41%
8	$Pd(CH_3CN)_4(BF_4)_2$	5 mol%	none	48%
9	$Pd(CH_3CN)_4(SbF_6)_2$	5 mol%	none	43% (43%)
10	$Pd(CH_3CN)_4(SbF_6)_2$	10 mol%	none	(33%)
11	$Pd(CH_3CN)_4(SbF_6)_2$	20 mol%	none	(25%)
12	$Pd(CH_3CN)_4(SbF_6)_2$	30 mol%	none	(19%)

(a) Reaction conditions: phenanthrene **1b** (0.2 mmol), dimethyldibenzosilole **2a** (1.5 equiv), Pd catalyst (x mol%), silver salt (10 mol%), *o*-chloranil (2.0 equiv), 1,2-dichloroethane (2 mL), 80 °C, 2 h. (b) Isolated yield. The numbers in the parentheses are GC yield calculated using *n*-dodecane as an internal standard.

Supplementary Table 3. Amount of dibenzosilole and *o*-chloranil [a]

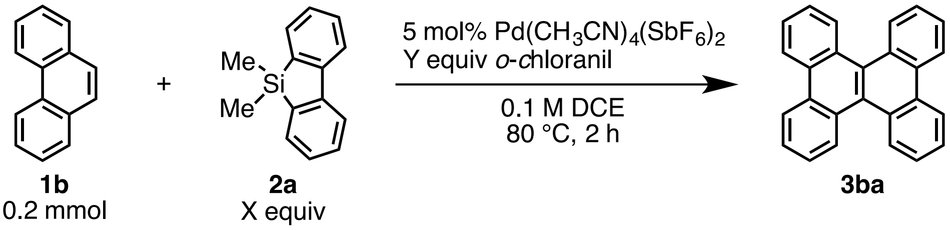

entry	X equiv	Y equiv	GC yield [b]
1	1.0	2.0	18%
2	1.25	2.0	25%
3	1.5	2.0	48% [c]
4	2.0	2.0	38%
5	2.5	2.0	44%
6	1.5	1.0	21%
7	1.5	1.5	27%
8	1.5	3.0	39%

(a) Reaction conditions: phenanthrene **1b** (0.2 mmol), dimethyldibenzosilole **2a** (X equiv), $Pd(CH_3CN)_4(SbF_6)_2$ (5 mol%), *o*-chloranil (Y equiv), 1,2-dichloroethane (2 mL), 80 °C, 2 h. (b) GC yield was calculated using *n*-dodecane as an internal standard. (c) Isolated yield.

Supplementary Table 4. Effect of solvent, concentration and reaction temperature [a]

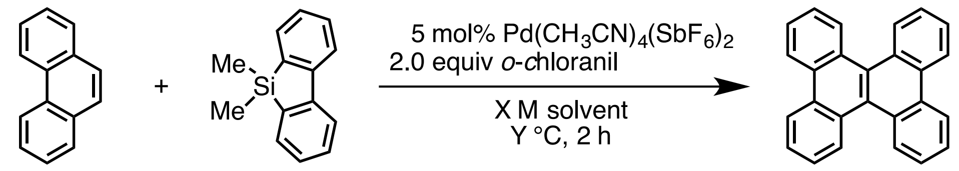

1b 0.2 mmol + **2a** 1.5 equiv → **3ba**

entry	solvent	X (M)	Y	GC yield [b]
1	DCE	0.1	80 °C	48% [c]
2	*o*-$Cl_2C_6H_4$	0.1	80 °C	23%
3	PhCl	0.1	80 °C	28%
4	$PhCF_3$	0.1	80 °C	41%
5	PhF	0.1	80 °C	31%
6	C_6F_6	0.1	80 °C	3%
7	toluene	0.1	80 °C	23%
8	DCE	0.2	80 °C	38%
9	DCE	0.05	80 °C	40%
10	DCE	0.025	80 °C	40%
11	DCE	0.02	80 °C	35%
12	DCE	0.1	140 °C	40%
13	DCE	0.1	120 °C	38%
14	DCE	0.1	100 °C	47%
15	DCE	0.1	60 °C	37%
16	DCE	0.1	40 °C	12%
17	DCE	0.1	rt	5%

(a) Reaction conditions: phenanthrene **1b** (0.2 mmol), dimethyldibenzosilole **2a** (1.5 equiv), $Pd(CH_3CN)_4(SbF_6)_2$ (5 mol%), *o*-chloranil (2.0 equiv), solvent (X M), Y °C, 2 h. (b) GC yield was calculated using *n*-dodecane as an internal standard. (c) Isolated yield.

Supplementary Methods

1. General

Unless otherwise noted, all materials including dry solvents were obtained from commercial suppliers and used without further purification. $PdCl_2$ was purchased from Wako. $AgSbF_6$ was purchased from Aldrich. *o*-Chloranil was purchased from TCI and recrystallized from benzene before use. Unless otherwise noted, all reactions were performed with dry solvents under an atmosphere of nitrogen or argon in oven-dried glassware with standard vacuum-line techniques. All work-up and purification procedures were carried out with reagent-grade solvents in air

Analytical thin-layer chromatography (TLC) was performed using E. Merck silica gel 60 F_{254} precoated plates (0.25 mm). Preparative thin-layer chromatography (PTLC) was performed using Wako-gel® B5-F silica coated plates (0.75 mm) prepared in our laboratory. Preparative gel permeation chromatography (GPC) was performed with a JAI LC-9204 instrument equipped with JAIGEL-1H/JAIGEL-2H columns using chloroform as an eluent. Gas chromatography (GC) analysis was conducted on a Shimadzu GC-2010 instrument equipped with a HP-5 column (30 m × 0.25 mm, Hewlett-Packard). The developed chromatogram was analyzed by UV lamp (254 nm and 365 nm). High-resolution mass spectra (HRMS) were obtained from a JMS-T100TD instrument (DART), Bruker Daltonics Ultraflex III TOF/TOF (MALDI-TOF-MS) and Thermo Fisher Scientific Exactive (ESI, APCI). Nuclear magnetic resonance (NMR) spectra were recorded on a JEOL ECS-600 (^{1}H 600 MHz, ^{13}C 150 MHz, ^{19}F 565 MHz) spectrometer and a JEOL ECA 600II with Ultra COOL™ probe (^{1}H 600 MHz, ^{13}C 150 MHz). Chemical shifts for ^{1}H NMR are expressed in parts per million (ppm) relative to tetramethylsilane (d 0.00 ppm). Chemical shifts for ^{13}C NMR are expressed in ppm relative to $CDCl_3$ (d 77.0 ppm). Chemical shifts for ^{19}F NMR are expressed in parts per million (ppm) relative to hexafluorobenzene (d –162.00 ppm) as an external standard. Data are reported as follows: chemical shift, multiplicity (s = singlet, d = doublet, dd = doublet of doublets, t = triplet, q = quartet, m = multiplet, br = broad signal), coupling constant (Hz), and integration.

2. Preparation of Substrates

2-1. Preparation of $Pd(CH_3CN)_4(SbF_6)_2$

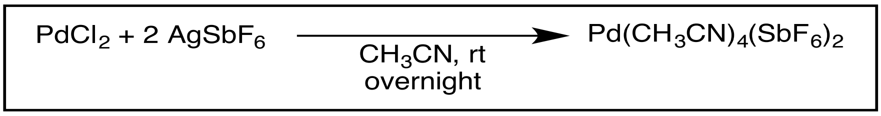

To a suspension of $PdCl_2$ (177 mg, 1.0 mmol) in CH_3CN (10 mL) was added $AgSbF_6$ (687 mg, 2.0 mmol), and the resultant mixture was stirred at room temperature overnight. The reaction mixture was filtrated, then Et_2O was added into the filtrate to recrystallize $Pd(CH_3CN)_4(SbF_6)_2$. The complex was collected by filtration (710 mg, 96%).

2-2. Preparation of 2,7-di-*tert*-butylphenanthrene (1a)

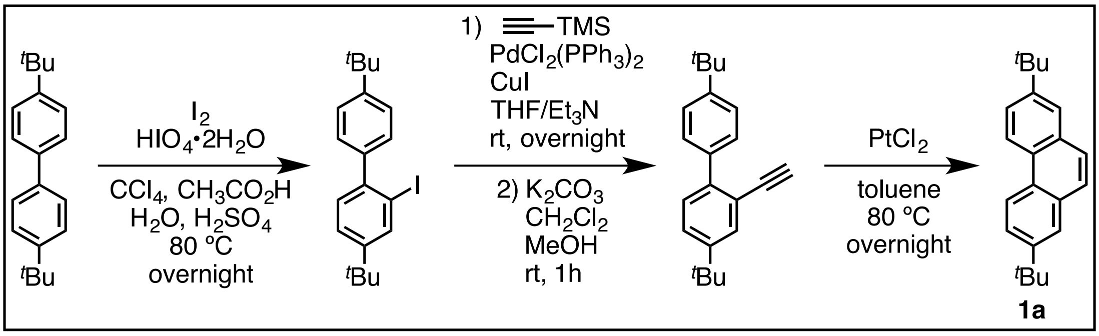

Synthesis of 4,4'-di-*tert*-butyl-2-ethynyl-1,1'-biphenyl[1]

To a solution of 4,4'-di-*tert*-butylbiphenyl (13.3 g 50 mmol) in $CCl_4/CH_3CO_2H/H_2O/H_2SO_4$ (20 mL/25 mL/5 mL/1 mL) were added iodine (12.7 g, 50 mmol) and $HIO_4 \cdot 2H_2O$ (5.7 g, 25 mmol), and the resulting reaction mixture was stirred at 80 °C overnight. After cooled to room temperature, sat. $NaHCO_3$ aq. was added to the reaction mixture and it was extracted with CH_2Cl_2. Combined organic layer was dried over Na_2SO_4, filtrated and concentrated under reduced pressure to give 4,4'-di-*tert*-butyl-2-iodo-1,1'-biphenyl. This crude product was used for the next step without purification.

A solution of 4,4'-di-*tert*-butyl-2-iodo-1,1'-biphenyl, trimethylsilylacetylene (7.4 g, 75 mmol), $PdCl_2(PPh_3)_2$ (1.75 g, 2.5 mmol), and CuI (476 mg, 2.5 mmol) in THF/triethylamine (100 mL/100 mL) was stirred at room temperature overnight. 1N HCl aq. was added to the reaction mixture and it was extracted with CH_2Cl_2. Combined organic layer was dried over Na_2SO_4, filtrated and concentrated under reduced pressure. The crude mixture was dissolved in $MeOH/CH_2Cl_2$ (50 mL/50 mL), and K_2CO_3 (6.9 g, 50 mmol) was added. Resulting suspension was stirred at room temperature for 1 h. Water was added and the organic layer was extracted with CH_2Cl_2. The combined organic phases were dried over $MgSO_4$, filtrated and concentrated under reduced pressure. Obtained crude 4,4'-di-*tert*-butyl-2-ethynyl-1,1'-biphenyl was used for the next step without purification.

PtCl$_2$-catalyzed synthesis of 2,7-di-*tert*-butylphenanthrene (1a)[2]

A solution of 4,4'-di-*tert*-butyl-2-ethynyl-1,1'-biphenyl and PtCl$_2$ (664 mg 2.5 mmol) in toluene (100 mL) was stirred at 80 °C overnight. The solvent was removed under reduced pressure. The residue was purified by silica gel column chromatography (eluent: hexane), and further purified by recrystallization from CH$_2$Cl$_2$/MeOH to obtain 2,7-di-*tert*-butylphenanthrene (**1a**) (5.0 g, 17 mmol, total 34% yield in 4 steps). ^{1}H NMR (600 MHz, CDCl$_3$) d 8.57 (d, J = 9.0 Hz, 2H), 7,82 (d, J = 2.4 Hz, 2H), 7.71 (dd, J = 9.0, 2.4 Hz, 2H), 7.70 (s, 2H), 1.45 (s, 18H); ^{13}C NMR (150 MHz, CDCl$_3$) d 148.9, 131.7, 128.1, 127.0, 124.8, 124.1, 122.3, 34.7, 31.4. HRMS (DRAT, ESI$^+$) *m/z* calcd for C$_{22}$H$_{27}$ [M+H]$^+$: 291.2113, found: 291.2112.

2-3. Preparation of silicon-bridged aromatics

Silicon-bridged aromatics **2a**[3], **2b**[4], **2c**[4], **2d**[4], **2e**[4], **2f**[4], **2g**[3], **2h**[4], **2i**[4], **2j**[4], **2k**[4], **2l**[4], **2m**[4], **4**[4], and **10**[5] were prepared from the corresponding biphenyls according to reported method.

Synthesis of 5,5-dimethyl-3,7-bis(4,4,5,5-tetramethyl-1,3,2-dioxaborolan-2-yl)-5*H*-dibenzo [*b*,*d*]siloles (2f)[6]

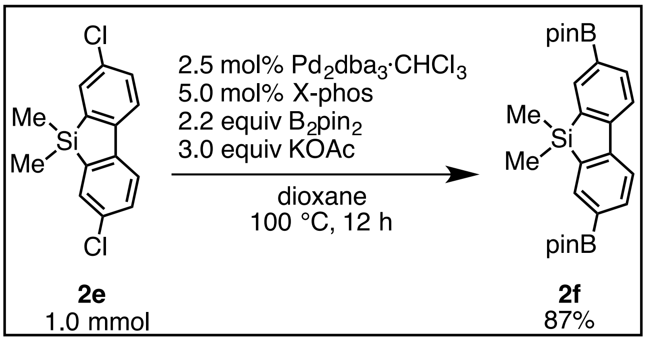

A solution of **2e** (279mg, 1.0 mmol), bis(pinacolate)diboron (559 mg, 2.2 mmol), Pd$_2$(dba)$_3$·CHCl$_3$ (tris(dibenzylideneacetone)dipalladium·CHCl$_3$, 26 mg, 0.025 mmol), XPhos (2-dicyclohexylphosphino-2',4',6'-triisopropylbiphenyl, 24 mg, 0.05 mmol), potassium acetate (294 mg, 3 mmol) in dry 1,4-dioxane (10 mL) was heated with stirring at 80 °C for 16 h. After the reaction mixture was cooled down to room temperature, the reaction was quenched with water. Then the mixture was extracted with EtOAc. The combined organic phases was dried over Na$_2$SO$_4$, filtered and concentrated under reduced pressure. The crude product was passed through a short pad of silica gel (eluent: EtOAc). After the organic solvent was removed under reduced pressure, the residue was purified by recrystallization from EtOH to yield **2f** (400 mg, 87%) as a white solid.

^{1}H NMR (600 MHz, CDCl$_3$) d 8.10 (s, 2H), 7.90 (dd, J = 7.9, 1.2 Hz, 2H), 7.86 (d, J = 7.2 Hz, 2H), 1.37 (s, 24H), 0.42 (s, 6H); ^{13}C NMR (150 MHz, CDCl$_3$) d 150.3, 139.2, 138.6, 136.9, 127.9 (br), 120.5, 83.7, 24.8, –3.2. HRMS (ESI$^+$) *m/z* calcd for C$_{26}$H$_{37}$O$_4$B$_2$Si [M+H]$^+$: 463.2642, found: 463.2626.

3. APEX Reaction of PAHs with Siloles

3-1. General procedure

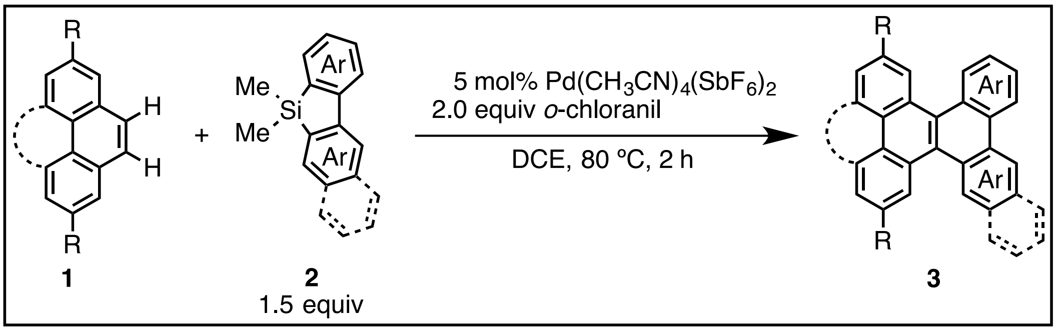

A solution of phenanthrene derivative **1** (0.20 mmol, 1.0 equiv), silicon-bridged aromatics **2** (0.30 mmol, 1.5 equiv), $Pd(CH_3CN)_4(SbF_6)_2$ (7.4 mg, 10 mmol, 5 mol%), and *o*-chloranil (98 mg, 0.40 mmol, 2.0 equiv) in DCE (2 mL) was stirred at 80 °C in the screw cap glass tube. After 2 hours, the reaction mixture was cooled to room temperature, and then passed through a short pad of silica gel (eluent: CH_2Cl_2). After the organic solvent was removed under reduced pressure, the residue was purified by PTLC or silica gel column chromatography to yield π-extended PAH.

3,14-Di-*tert*-butyldibenzo[*g*,*p*]chrysene (3aa)

PTLC: hexane only, Yield: 77.0 mg, 88%.

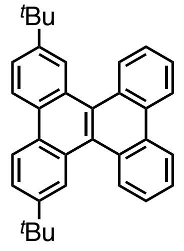

^{1}H NMR (600 MHz, $CDCl_3$) d 8.693 (dd, J = 10.4, 1.2 Hz, 4H), 8.687 (s, 2H), 8.58 (d, J = 9.0 Hz, 2H), 7.70 (dd, J = 8.4, 1.8 Hz, 2H), 7.65 (t, J = 6.9 Hz, 2H), 7.62 (t, J = 7.5 Hz, 2H), 1.44 (s, 18H); ^{13}C NMR (150 MHz, $CDCl_3$) d 148.8, 130.8, 129.5, 128.7, 128.6, 127.9, 126.40, 126.37, 125.2, 124.4, 123.6, 123.1, 35.0, 31.4. One aromatic carbon signal can be overlapped. HRMS (DART, ESI$^+$) *m/z* calcd for $C_{34}H_{33}$ $[M+H]^+$: 441.2582, found: 441.2579.

Dibenzo[*g*,*p*]chrysene (3ba)[7] CAS:191-68-4

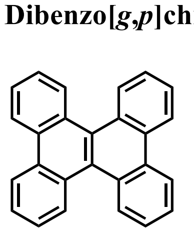

PTLC: hexane only, Yield: 31.3 mg, 48%

6,11-Di-*tert*-butyl-3-(trifluoromethyl)dibenzo[*g*,*p*]chrysene (3ab)

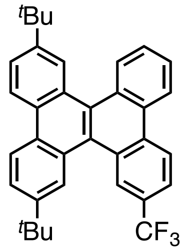

PTLC: hexane only, Yield: 90.5 mg, 89%.

^{1}H NMR (600 MHz, $CDCl_3$) d 9.03 (s, 1H), 8.78 (d, J = 8.4 Hz, 1H), 8.75–8.69 (m, 2H), 8.68 (d, J = 1.8 Hz, 1H), 8.62–8.57 (m, 3H), 7.86 (dd, J = 8.7, 1.5 Hz, 1H), 7.74 (dd, J = 8.4, 1.8 Hz, 1H), 7.73 (dd, J = 8.7, 2.1 Hz, 1H), 7.71–7.67 (m, 2H), 1.442 (s,

9H), 1.440 (s, 9H); ^{13}C NMR (150 MHz, CDCl$_3$) d 149.5, 149.1, 132.8, 130.2, 129.9, 129.0, 128.9, 128.8 (2C), 128.7, 128.4, 128.20 ($^2J_{C–F}$ = 32.1 Hz), 128.17, 127.4, 127.3, 126.7, 126.3 ($^3J_{C–F}$ = 4.3 Hz),125.2, 125.02, 124.96, 124.8, 124.6 ($^1J_{C–F}$ = 271 Hz), 124.3, 124.0, 123.25, 123.22, 122.2 ($^3J_{C–F}$ = 4.4 Hz), 35.04, 35.02, 31.4, 31.3; ^{19}F NMR (565 MHz, CDCl$_3$) d –62.25 (s). HRMS (DART, ESI$^+$) *m/z* calcd for $C_{35}H_{32}F_3$ [M+H]$^+$: 509.2456, found: 509.2456.

6,11-Di-*tert*-butyl-3-fluorodibenzo[g,p]chrysene (3ac)

PTLC: hexane only, Yield: 84.9 mg, 93%.

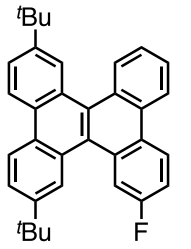

^{1}H NMR (600 MHz, CDCl$_3$) d 8.70–8.65 (m, 3H), 8.65 (d, *J* = 2.4 Hz, 1H), 8.63 (dd, *J* = 8.4, 1.2 Hz, 1H), 8.60 (dd, *J* = 9.0, 1.2 Hz, 2H), 8.38 (dd, *J* = 11.7, 2.7 Hz, 1H), 7.74 (dt, *J* = 8.4, 2.3 Hz, 2H), 7.67 (td, *J* = 7.8, 1.2 Hz, 1H), 7.62 (td, *J* = 7.5, 1.2 Hz, 1H), 7.40 (ddd, *J* = 9.0, 7.8, 3.0 Hz, 1H), 1.46 (s, 9H), 1.45 (s 9H); ^{13}C NMR (150 MHz, CDCl$_3$) d 161.5 ($^1J_{C–F}$ = 242.9 Hz), 149.2, 149.0, 131.0 ($^3J_{C–F}$ = 8.6 Hz), 130.5, 129.1, 129.0, 128.9, 128.7, 128.6, 128.5 (2C), 127.3, 127.2 ($^4J_{C–F}$ = 2.8 Hz), 126.7, 126.2, 125.8 ($^3J_{C–F}$ = 8.6 Hz), 125.4, 124.8, 124.7, 124.5, 123.5, 123.2, 123.1, 114.7 ($^2J_{C–F}$ = 23.1 Hz), 113.7 ($^2J_{C–F}$ = 23.0 Hz), 35.0, 31.4; ^{19}F NMR (565 MHz, CDCl$_3$) d –114.87 (t, *J* = 13.0 Hz). HRMS (DART, ESI$^+$) *m/z* calcd for $C_{34}H_{32}F$ [M+H]$^+$: 459.2488, found: 459.2484.

6,11-Di-*tert*-butyl-3-chlorodibenzo[*g*,*p*]chrysene (3ad)

PTLC: hexane only, Yield: 84.0 mg, 88%.

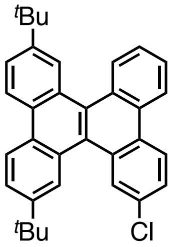

^{1}H NMR (600 MHz, CDCl$_3$) d 8.76 (s, 1H), 8.74–8.70 (m, 2H), 8.68–8.65 (m, 2H), 8.64 (d, *J* = 8.4 Hz, 1H), 8.61 (d, *J* = 8.4 Hz, 2H), 7.75 (dt, *J* = 9.0, 2.1 Hz, 2H), 7.71–7.65 (m, 2H), 7.63 (dd, *J* = 8.7, 2.1 Hz, 1H), 1.50 (s, 9H), 1.48 (s, 9H); ^{13}C NMR (150 MHz, CDCl$_3$) d 149.2, 149.0, 132.4, 130.6, 130.2, 129.5, 129.0, 128.9 (2C), 128.7, 128.6, 128.5, 128.3, 128.1, 126.8, 126.7, 126.6, 126.5, 125.3, 125.2, 124.9, 124.8, 124.6, 123.5, 123.2 (2C), 35.04, 35.02, 31.42, 31.39. HRMS (DART, ESI$^+$) *m/z* calcd for $C_{34}H_{32}Cl$ [M+H]$^+$: 475.2193, found: 475.2179.

3,14-Di-*tert*-butyl-6,11-dichlorodibenzo[*g*,*p*]chrysene (3ae)

Silica gel column chromatography: hexane/EtOAc = 10:1 to 5:1, Yield: 71.4 mg, 70%.

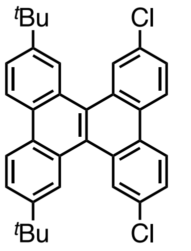

^{1}H NMR (600 MHz, CDCl$_3$) d 8.72 (d, *J* = 1.8 Hz, 2H), 8.64 (d, *J* = 1.8 Hz, 2H), 8.62 (d, *J* = 9.0 Hz, 2H), 8.58 (d, *J* = 9.0 Hz, 2H), 7.77 (dd, *J* = 9.0, 1.8 Hz, 2H), 7.63 (dd, *J* = 9.0, 2.4 Hz, 2H), 1.48 (s, 18H); ^{13}C NMR (150 MHz, CDCl$_3$) d 149.3, 132.7, 130.5, 128.7, 128.5, 128.3, 128.1, 127.7, 126.8, 125.0 (2C), 124.9, 123.2, 35.0, 31.4. HRMS (DART, ESI$^+$) *m/z* calcd for $C_{34}H_{31}Cl_2$ [M+H]$^+$: 509.1803, found: 509.1805.

2,2'-(6,11-Di-*tert*-butyldibenzo[*g*,*p*]chrysene-3,14-diyl)bis(4,4,5,5-tetramethyl-1,3,2-dioxaborolane) (3af)

Silica gel column chromatography: hexane/EtOAc = 10:1 to 6:1, Yield: 34.1 mg, 25%.

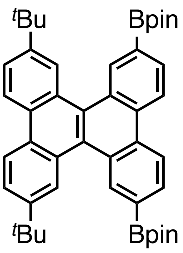

^{1}H NMR (600 MHz, $CDCl_3$) d 9.31 (s, 2H), 8.78 (d, *J* = 1.8 Hz, 2H), 8.74 (d, *J* = 8.0 Hz, 2H), 8.61 (d, *J* = 9.0 Hz, 2H), 8.05 (dd, *J* = 7.8, 0.6 Hz, 2H), 7.73 (dd, *J* = 8.4, 1.8 Hz, 2H), 1.49 (s, 18H), 1.37 (s, 24H); ^{13}C NMR (150 MHz, $CDCl_3$) d 148.7, 136.9, 132.6, 131.4, 129.1, 128.7, 128.6, 127.9, 126.6 (br), 126.1, 124.2, 122.9, 122.8, 83.9, 35.1, 31.5, 25.0. HRMS (ESI$^+$) *m/z* calcd for $C_{46}H_{55}O_4B_2$ $[M+H]^+$: 693.4281, found: 693.4282.

3,6,11,14-Tetra-*tert*-butyldibenzo[*g*,*p*]chrysene (3ag)

PTLC: hexane only, Yield: 69.0 mg, 63%.

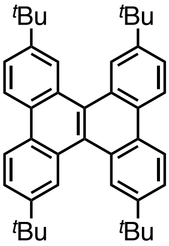

^{1}H NMR (600 MHz, $CDCl_3$) d 8.65 (d, *J* = 1.8 Hz, 4H), 8.59 (d, *J* = 8.4 Hz, 4H), 7.71 (dd, *J* = 8.7, 1.5 Hz, 4H), 1.44 (s, 36H); ^{13}C NMR (150 MHz, $CDCl_3$) d 148.8, 129.1, 128.5, 128.4, 124.9, 124.3, 123.2, 35.1, 31.6. HRMS (DART, ESI$^+$) *m/z* calcd for $C_{42}H_{49}$ $[M+H]^+$: 553.3834, found: 553.3854.

6,11-Di-*tert*-butyl-3-methoxydibenzo[*g*,*p*]chrysene (3ah)

PTLC: hexane/CH_2Cl_2 = 4:1, Yield: 69.6 mg, 74%.

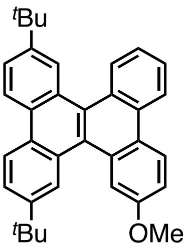

^{1}H NMR (600 MHz, $CDCl_3$) d 8.77 (d, *J* = 1.2 Hz, 1H), 8.69 (d, *J* = 1.2 Hz, 1H), 8.66 (d, *J* = 8.4 Hz, 1H), 8.63–8.58 (m, 4H), 8.17 (d, *J* = 3.0 Hz, 1H), 7.75–7.71 (m, 2H), 7.65 (t, *J* = 7.5 Hz, 1H), 7.57 (t, *J* = 7.5 Hz, 1H), 7.30 (dd, *J* = 9.0, 2.4 Hz, 1H), 3.94 (s, 3H) 1.46 (s, 9H), 1.45 (s, 9H); ^{13}C NMR (150 MHz, $CDCl_3$) d 158.3, 148.9, 148.8, 130.9, 130.7, 128.9, 128.8 (3C), 128.7, 128.6, 128.5, 127.7, 126.5, 125.5, 125.4, 125.2, 124.9, 124.50, 124.48, 124.39, 123.3, 123.11, 123.06, 116.4, 109.9, 55.4, 35.1, 35.0, 31.6, 31.4. HRMS (DART, ESI$^+$) *m/z* calcd for $C_{35}H_{35}O$ $[M+H]^+$: 471.2688, found: 471.2681.

6,11-Di-*tert*-butyl-1-methoxydibenzo[g,p]chrysene (3ai)

PTLC: hexane only, Yield: 53.4 mg, 57%.

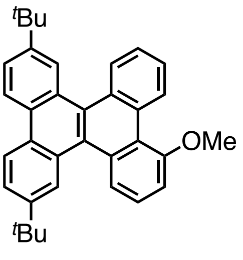

^{1}H NMR (600 MHz, $CDCl_3$) d 9.43–9.39 (m, 1H), 8.67–8.64 (m, 2H), 8.63–8.60 (m, 1H), 8.55 (dd, *J* = 9.0, 1.2 Hz, 2H), 8.29 (d, *J* = 8.4 Hz, 1H), 7.69 (t, *J* = 7.8 Hz, 2H), 7.60–7.56 (m, 2H), 7.53 (t, *J* = 8.1 Hz, 1H), 7.15 (d, *J* = 7.8 Hz, 1H), 4.10 (s, 3H), 1.43 (s, 18H); ^{13}C NMR (150 MHz, $CDCl_3$) d 157.8, 148.8, 148.6, 132.0, 130.3, 129.2, 128.8, 128.75, 128.69 (2C), 128.6,

128.4, 127.9 (2C), 126.7, 126.2, 125.5, 125.2 (2C), 124.4, 124.2, 123.04, 123.02, 121.2, 120.7, 108.5, 55.9, 34.9 (2C), 31.4 (2C). HRMS (DART, ESI$^+$) *m/z* calcd for $C_{35}H_{35}O$ [M+H]$^+$: 471.2688, found: 471.2679.

3,16-Di-*tert*-butyltribenzo[*a*,*c*,*f*]tetraphene (3aj)

PTLC: hexane only, Yield: 46.4 mg, 47%.

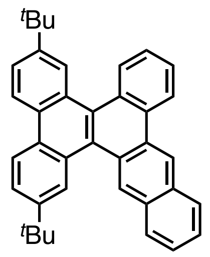

^{1}H NMR (600 MHz, CDCl$_3$) d 9.15 (s, 1H), 9.12 (s, 1H), 8.89 (s, 1H), 8.80 (d, *J* = 7.8 Hz, 1H), 8.69 (s, 1H), 8.63–8.57 (m, 3H), 8.12 (d, *J* = 7.8 Hz, 1H), 7.97 (d, *J* = 8.4 Hz, 1H), 7.72 (dd, *J* = 8.4, 1.8 Hz, 1H), 7.70 (dd, *J* = 8.7, 1.5 Hz, 1H), 7.67 (t, *J* = 7.5 Hz, 1H), 7.62 (t, *J* = 7.5 Hz, 1H), 7.58–7.50 (m, 2H), 1.45 (s, 9H), 1.44 (s, 9H); ^{13}C NMR (150 MHz, CDCl$_3$) d 148.9, 148.8, 132.0, 131.6, 131.3, 130.2, 129.9, 128.9, 128.8, 128.7, 128.6, 128.42, 128.39, 128.2, 128.1, 128.0, 127.9 (2C), 126.8, 126.7, 126.0, 125.9, 125.1, 124.9, 124.5, 124.4, 124.3, 123.1, 122.2, 35.0 (2C), 31.44, 31.42. One aromatic carbon signal can be overlapped. HRMS (DART, ESI$^+$) *m/z* calcd for $C_{38}H_{35}$ [M+H]$^+$: 491.2739, found: 491.2722.

***4H*-Benzo[*p*]indeno[7,1,2-*ghi*]chrysene (3ca)**

A solution of 4*H*-Cyclopenta[*def*]phenanthrene **1c** (19 mg, 0.10 mmol, 1.0 equiv), **2a** (32 mg, 0.15 mmol, 1.5 equiv), Pd(CH_3CN)$_4$(SbF_6)$_2$ (3.7 mg, 5 mmol, 5 mol%), and *o*-chloranil (49 mg, 0.20 mmol, 2.0 equiv) in DCE (1 mL) was stirred at 80 °C in the screw cap glass tube. After 2 hours, the reaction mixture was cooled to room temperature, and then passed through a short pad of silica gel (eluent: CH_2Cl_2). After the organic solvent was removed under reduced pressure, the residue was purified by silica gel column chromatography (eluent: hexane/CH_2Cl_2 = 7:1) to give **3ca** (17.3 mg, 51%).

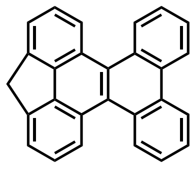

^{1}H NMR (600 MHz, CDCl$_3$) d 9.10 (dd, *J* = 7.8, 1.2 Hz, 2H), 8.79 (dd, *J* = 7.8, 1.2 Hz, 2H), 8.72 (d, *J* = 7.8 Hz, 2H), 7.81–7.76 (m, 4H), 7.75–7.68 (m, 4H), 4.45 (s, 2H); ^{13}C NMR (150 MHz, CDCl$_3$) d 141.6, 138.4, 130.8, 130.4, 128.5, 128.2, 127.1, 126.68, 126.66, 125.7, 124.7, 123.5, 121.6, 37.4. HRMS (DART, ESI$^+$) *m/z* calcd for $C_{27}H_{17}$ [M+H]$^+$: 341.1330, found: 341.1331.

3-2. 2:1 APEX reaction of 1a with 4.

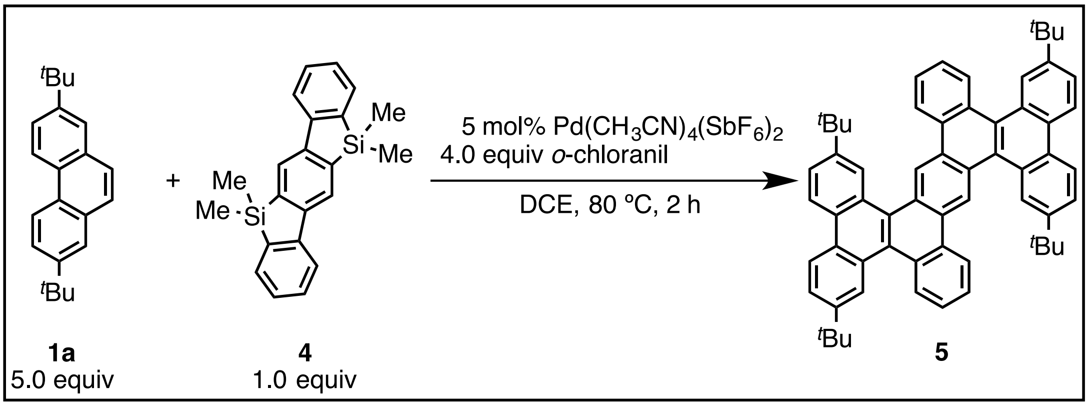

A solution of 2,7-di-*tert*-butylphenanthrene (**1a**) (145 mg, 0.5 mmol, 5.0 equiv), **4** (34.3 mg, 0.1 mmol, 1.0 equiv), $Pd(CH_3CN)_4(SbF_6)_2$ (7.4 mg, 10 mmol, 5 mol%), and *o*-chloranil (98 mg, 0.4 mmol, 4.0 equiv) in DCE (2 mL) was stirred at 80 °C for 2 h in the screw cap glass tube. After the reaction mixture was cooled to room temperature, the mixture was passed through a short pad of silica gel (eluent: CH_2Cl_2). After the organic solvent was removed under reduced pressure, the residue was purified by silica gel column chromatography (eluent: hexane/CH_2Cl_2 = 19:1) to give **5** (55.3 mg, 69% yield).

^{1}H NMR (600 MHz, $CDCl_3/CS_2$) d 9.90 (s, 2H), 9.07 (s, 2H), 8.85 (d, J = 7.8 Hz, 2H), 8.72–8.68 (m, 4H), 8.66 (d, J = 8.4 Hz 2H), 8.62 (d, J = 9.0 Hz, 2H), 7.81 (d, J = 9.0 Hz, 2H), 7.71 (d, J = 9.0 Hz, 2H), 7.66–7.61 (m, 4H), 1.56 (s, 18H), 1.44 (s, 18H); ^{13}C NMR (150 MHz, $CDCl_3/CS_2$) d 149.1, 148.7, 131.1, 130.0, 129.4, 129.1, 129.0, 128.8, 128.7, 128.6, 128.3, 128.2, 128.0, 126.53, 126.49, 125.1 (2C), 124.6, 124.4, 123.8, 123.7, 123.3, 123.1, 35.0, 34.9, 31.6, 31.4. HRMS (APCI) *m/z* calcd for $C_{62}H_{59}$ $[M+H]^+$: 803.4611, found: 803.4576.

3-3. 1:2 APEX reaction of 6 with 2a (gram-scale synthesis of 10,21-di-*tert*-butylhexabenzo[*a*,*c*,*fg*,*j*,*l*,*op*]tetracene (7))

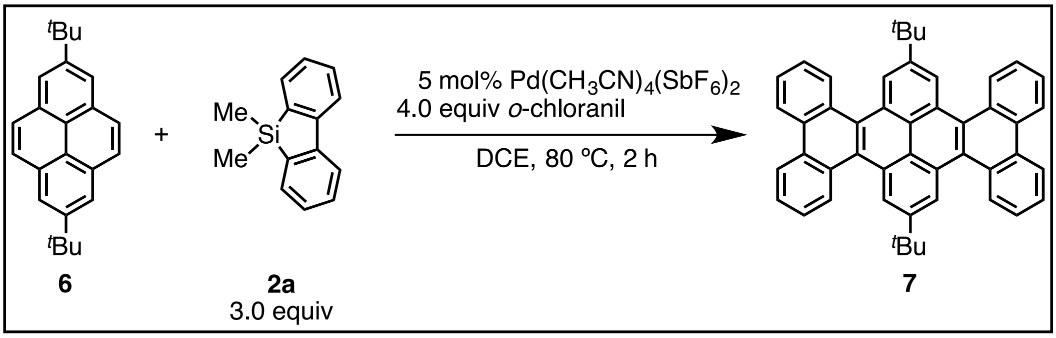

A solution of 2,7-di-*tert*-butylpyrene (**6**, 1.57 g, 5.0 mmol, 1.0 equiv), **2a** (3.15 g, 15 mmol, 3.0 equiv), $Pd(CH_3CN)_4(SbF_6)_2$ (185.5 mg, 0.25 mmol, 5 mol%), and *o*-chloranil (4.92 g, 20 mmol, 4.0 equiv) in DCE (50 mL) was stirred at 80 °C in the screw cap glass tube. After 8 h, the reaction mixture was cooled to room temperature, and then the organic solvent was removed under reduced pressure.

The residue was purified by silica gel column chromatography (eluent: hexane/CH_2Cl_2 = 9:1) to give 10,21-di-*tert*-butylhexabenzo[*a,c,fg,j,l,op*]tetracene (**7**) (2.6 g, 83% yield). ^{1}H NMR ($CDCl_3$, 600 MHz) d 9.05 (s, 4H), 8.90 (d, *J* = 8.2 Hz, 4H), 8.82 (d, *J* = 8.2 Hz, 4H), 7.76 (t, *J* = 8.2 Hz, 4H), 7.70 (t, *J* = 8.2 Hz, 4H), 1.61 (s, 18H); ^{13}C NMR ($CDCl_3$, 100 MHz) d 147.7, 131.0, 129.9, 128.5, 128.3, 127.9, 126.7, 126.6, 123.8, 123.4, 122.8, 35.7, 31.8. HRMS (DART, ESI$^+$) *m/z* calcd for $C_{48}H_{39}$ [M+H]$^+$: 615.3052, found: 615.3067.

3-4. Preparation of 7,7'-di-*tert*-butyl-2,2'-bipyrene (8).

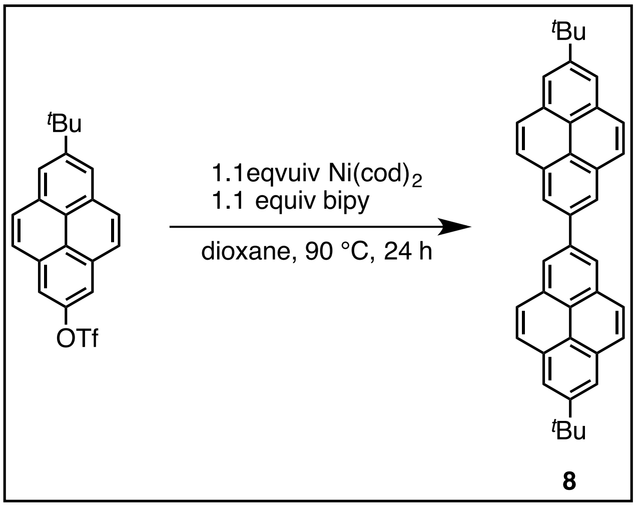

A solution of 7-(*tert*-butyl)pyren-2-yl trifluoromethanesulfonate (81 mg, 0.20 mmol, 1.0 equiv), bis(1,5-cyclooctadiene)nickel (60 mg, 0.22 mmol, 1.1 equiv), and 2,2'-bipyridyl (34 mg, 0.22 mmol) in dry 1,4-dioxane (2 mL) was stirred at 90 °C in 20-mL glass vessel tubes equipped with J. Young® O-ring tap containing a magnetic stirring bar. After 14 h, the reaction mixture was cooled to room temperature, passed through a short pad of silica gel (eluent: CH_2Cl_2). After cooled down to room temperature, the mixture was concentrated under reduced pressure. The mixture was extracted with $CHCl_3$. The combined organic phase was dried over Na_2SO_4 and concentrated under reduced pressure. The crude product was purified by silica gel column chromatography (eluent: hexane/CH_2Cl_2 = 6:1) to give **8** (102.6 mg, 99% yield).

^{1}H NMR (600 MHz, $CDCl_3$) d 8.64 (s, 4H), 8.26 (s, 4H), 8.18 (d, *J* = 9.0 Hz, 4H), 8.12 (d, *J* = 9.0 Hz, 4H), 1.61 (s, 18H); ^{13}C NMR (150 MHz, $CDCl_3$) d 149.2, 139.0, 131.6, 131.1, 128.1, 127.5, 124.4, 123.9, 122.9, 122.5, 35.3, 32.0. HRMS (APCI) *m/z* calcd for $C_{40}H_{35}$ [M+H]$^+$: 515.2733, found: 515.2727.

3-5. 1:4 APEX reaction of 8 with 2a.

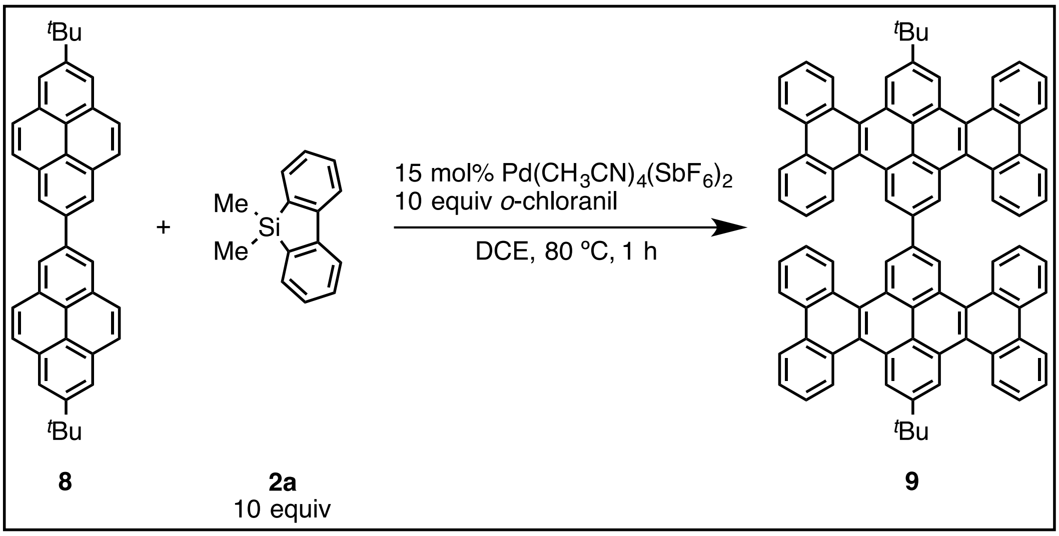

A solution of 7,7'-di-*tert*-butyl-2,2'-bipyrene (**8**) (51 mg, 0.10 mmol, 1.0 equiv), **2a** (210 mg, 1.0 mmol, 10 equiv), $Pd(CH_3CN)_4(SbF_6)_2$ (11 mg, 0.015 mmol, 15 mol%), and *o*-chloranil (245 mg, 1.0 mmol, 10 equiv) in DCE (4 mL) was stirred at 80 °C in the screw cap glass tube. After 1 h, the reaction mixture was cooled to room temperature, and the residue was subjected to silica gel column chromatography (eluent: hexane/CH_2Cl_2 = 4:1), and then purified by PTLC (eluent: $CHCl_3$) to give 21,21'-di-*tert*-butyl-10,10'-bihexabenzo[*a*,*c*,*fg*,*j*,*l*,*op*]tetracene (**9**) (34.3 mg, 31% yield).

^{1}H NMR (600 MHz, $CDCl_3$) d 9.65 (s, 4H), 9.11 (s, 4H), 9.08 (d, J = 8.4 Hz, 4H), 8.94 (d, J = 7.2 Hz, 4H), 8.85 (d, J = 7.2 Hz, 8H), 7.80–7.76 (m, 8H), 7.73 (t, J = 7.5 Hz, 4H), 7.49 (t, J = 7.8 Hz, 4H), 1.64 (s, 18H); ^{13}C NMR (150 MHz, $CDCl_3$) d 148.3, 137.9, 131.2, 131.0, 129.83, 129.76, 129.0, 128.7, 128.6, 128.4, 128.2, 127.3, 126.9, 126.74, 126.68, 125.2, 123.9, 123.7, 122.7, 35.8, 31.8. HRMS (APCI) *m/z* calcd for $C_{88}H_{59}$ $[M+H]^+$: 1115.4611, found: 1115.4556.

3-6. 2:1 APEX reaction of 6 with 10.

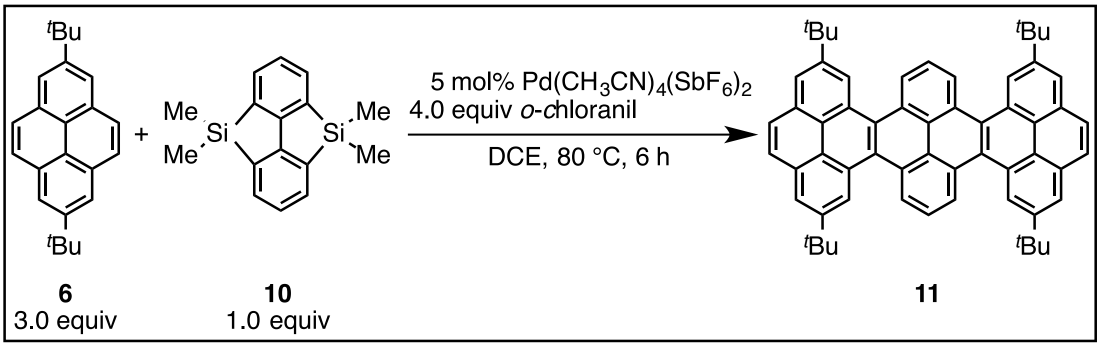

A solution of 2,7-di-*tert*-butylpyrene (**6**) (47 mg, 0.15 mmol, 3.0 equiv), 4,4,8,8-tetramethyl-4,8-dihydro-4,8-disilacyclopenta[*def*]fluorene (**10**) (13 mg, 0.05 mmol, 1.0 equiv), $Pd(CH_3CN)_4(SbF_6)_2$ (2 mg, 2.5 mmol, 5 mol%), and *o*-chloranil (49 mg, 0.2 mmol, 4.0 equiv) in DCE (2 mL) was stirred at 80 °C in the screw cap glass tube. After 6 h, the reaction mixture was cooled to room temperature, and the residue was subjected to silica gel column chromatography (eluent:

hexane/CH_2Cl_2 = 4:1), and then purified by preparative recycling gel permeation chromatography ($CHCl_3$) to give 2,7,13,18-tetra-*tert*-butylhexabenzo[*de,hi,lm,qr,uv,yz*]hexacene (**11**) (20.6 mg, 53% yield).

^{1}H NMR (600 MHz, $CDCl_3$) d 9.31 (d, J = 1.8 Hz, 4H), 9.26 (d, J = 8.4 Hz, 4H), 8.28 (d, J = 1.8 Hz, 4H), 8.18–8.14 (m, 6H), 1.64 (s, 36H); ^{13}C NMR (150 MHz, $CDCl_3$) d 148.2, 131.0, 129.3, 128.8, 128.4, 127.5, 125.4 (2C), 124.7, 123.9, 123.2, 122.1, 35.5, 31.9. HRMS (APCI) *m/z* calcd for $C_{60}H_{55}$ $[M+H]^+$: 775.4298, found: 775.4269.

3-7. 1:2 APEX reaction of 11 with 2a.

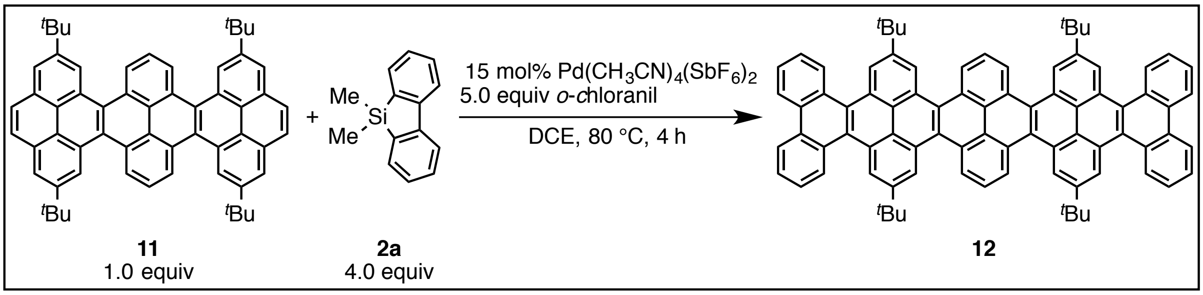

A solution of **11** (18 mg, 22.8 mmol, 1.0 equiv), **2a** (19 mg, 91.3 mmol, 4.0 equiv), $Pd(CH_3CN)_4(SbF_6)_2$ (2.0 mg, 3.4 mmol, 15 mol%), and *o*-chloranil (28 mg, 114 mmol, 5.0 equiv) in DCE (0.5 mL) was stirred at 80 °C in the screw cap glass tube. After 4 h, the reaction mixture was cooled to room temperature, and the residue was subjected to silica gel column chromatography (eluent: hexane/CH_2Cl_2 = 6:1), and then purified by preparative recycling gel permeation chromatography (eluent: $CHCl_3$) to give 10,16,27,33-tetra-*tert*-butyldecabenzo[*a*,*a$_1$b$_1$*,*c*,*e$_1$f$_1$*,*fg*,*jk*,*no*,*r*,*t*,*wx*]octacene (**12**) (4.1 mg, 17% yield).

^{1}H NMR (600 MHz, $CDCl_3$) d 9.33 (d, J = 1.2 Hz, 4H), 9.28 (d, J = 7.8 Hz, 4H), 9.15 (d, J = 1.2 Hz, 4H), 8.98 (d, J = 7.8 Hz, 4H), 8.85 (d, J = 7.2 Hz, 4H), 8.22 (t, J = 8.1 Hz, 2H), 7.79 (td, J = 7.2, 1.8 Hz, 4H), 7.74 (td, J = 7.8, 1.2 Hz, 4H), 1.67 (s, 36H); ^{13}C NMR (150 MHz, $CDCl_3$) d 147.9, 131.1, 129.9, 129.2, 128.8, 128.6, 128.4 (2C), 128.0, 126.8, 126.7, 125.6, 125.5, 125.0, 123.8, 123.7, 123.25, 123.15, 35.8, 31.9. HRMS (APCI) *m/z* calcd for $C_{84}H_{67}$ $[M+H]^+$: 1075.5237, found: 1075.5209.

4. Photophysical Measurement

UV/vis absorption spectra of **6**, **11** and **12** in chloroform were recorded on a JASCO UV-570 spectrometer with a resolution of 0.5 nm. Emission spectrum of **6**, **11** and **12** in chloroform were measured with a JASCO FP-6600 spectrometer with a resolution of 0.4 nm upon excitation at 280 nm for **6**, 325 nm for **11** and 345 nm for **12**. Dilute solution in spectral grade chloroform in a 1 cm square quartz cell was used for measurements. For details, see Supplementary Figure 41.

5. Computational Study

Computational methodology

All the calculations were carried out using the Gaussian 09 program package[8]. Geometry optimizations were performed at the density functional theory (DFT) level using the Becke's three-parameter hybrid functional[9], PW91 non-local correlation functional[10] (B3PW91) in conjunction with following basis sets; LanL2DZ[11,12] with effective core potential (ECP) for Pd, 6-31+G*[13,14,15] for O, C on phenanthrene and phenyl group due to the necessity of diffuse function to describe the extra electron placed far from the nuclei in the atoms, 6-31G* for Cl and other C atoms, 6-31G** for H on phenanthrene and 6-31G for other H atoms (called BS1). All structures were optimized without any symmetry assumptions and verified to be minima (no imaginary frequencies) or transition states (one imaginary frequency) on the free energy surface with analytical frequency calculations with B3PW91/BS1 level of theory. The transition states were confirmed by full intrinsic reaction coordinate[16,17] (IRC) calculations at the B3PW91/BS1 level. Full IRC calculations allowed displaying the direct connection between transition states and their corresponding reactants and products. For single-point energy calculations we employed the spin-component-scaled Møller-Plesset second-order perturbation theory[18] (SCS-MP2) to obtain more reliable energy because it usually gives results close to the experimental value. With SCS-MP2 theory we used larger basis sets; Stuttgard-Dresden basis set[19] (SDD) with ECP for Pd, 6-311+G* for O, C on phenanthrene and phenyl group, 6-311G* for Cl and other C atoms, 6-311G** for H (called BS2). Zero-point energy, enthalpy, and Gibbs free energy at 353.15 K and 1 atm were estimated from the gas-phase studies. For calculation details, see Supplementary Figure 42 and Supplementary Table 1.

Supplementary References

(1) Tashiro, M. & Yamato, T. Studies on selective preparation of aromatic compounds. 20. Selective preparation of 2-mono- and 2,2'-disubstituted biphenyl using the *tert*-butyl group as a positional protective group. *J. Org. Chem.* **44**, 3037–3041 (1979).

(2) Fürstner, A. & Mamane, V. Flexible synthesis of phenanthrenes by a $PtCl_2$-catalyzed cycloisomerization reaction. *J. Org. Chem.* **67**, 6264–6267 (2002).

(3) Neugebauer, W., Kos, A. J. & Schleyer, P. v. R. Regioselektive dimetallierung von aromaten. Bequemer zugang zu 2,2′-disubstituierten biphenylderivaten. *J. Organomet. Chem.* **228**, 107–118 (1982).

(4) Ureshino, T., Yoshida, T., Kuninobu, Y. & Takai, K. Rhodium-catalyzed synthesis of silafluorene derivatives via cleavage of silicon−hydrogen and carbon−hydrogen bonds. *J. Am. Chem. Soc.* **132**, 14324–14326 (2010).

(5) Shimizu, M., Tatsumi, H., Mochida, K., Oda, K. & Hiyama, T. Silicon-bridge effects on photophysical properties of silafluorenes. *Chem. Asian J.* **3**, 1238–1247 (2008).

(6) Omachi, H., Matsuura, S., Segawa, Y. & Itami, K. A modular and size-selective synthesis of [*n*]cycloparaphenylenes: A step toward the selective synthesis of [*n*,*n*] single-walled carbon nanotubes. *Angew. Chem. Int. Ed.* **49**, 10202–10205 (2010).

(7) Li, C.-W., Wang, C.-I., Liao, H.-Y., Chaudhuri, R. & Liu, R.-S. Synthesis of dibenzo[*g,p*]chrysenes from bis(biaryl)acetylenes via sequential ICl-induced cyclization and Mizoroki−Heck coupling. *J. Org. Chem.* **72**, 9203–9207 (2007).

(8) Frisch, M. J., Trucks, G. W., Schlegel, H. B., Scuseria, G. E., Robb, M. A., Cheeseman, J. R., Montgomery, J., J. A., Vreven, T., Kudin, K. N., Burant, J. C., Millam, J. M., Iyengar, S. S., Tomasi, J., Barone, V., Mennucci, B., Cossi, M., Scalmani, G., Rega, N., Petersson, G. A., Nakatsuji, H., Hada, M., Ehara, M., Toyota, K., Fukuda, R., Hasegawa, J., Ishida, M., Nakajima, T., Honda, Y., Kitao, O., Nakai, H., Klene, M., Li, X., Knox, J. E., Hratchian, H. P., Cross, J. B., Bakken, V., Adamo, C., Jaramillo, J., Gomperts, R., Stratmann, R. E., Yazyev, O., Austin, A. J., Cammi, R., Pomelli, C., Ochterski, J. W., Ayala, P. Y., Morokuma, K., Voth, G. A., Salvador, P., Dannenberg, J. J., Zakrzewski, V. G., Dapprich, S., Daniels, A. D., Strain, M. C., Farkas, O., Malick, D. K., Rabuck, A. D., Raghavachari, K., Foresman, J. B., Ortiz, J. V., Cui, Q., Baboul, A. G., Clifford, S., Cioslowski, J., Stefanov, B. B., Liu, G., Liashenko, A., Piskorz, P., Komaromi, I., Martin, R. L., Fox, D. J., Keith, T., Al-Laham, M. A., Peng, C. Y., Nanayakkara, A., Challacombe, M., Gill, P. M. W., Johnson, B., Chen, W., Wong, M. W., Gonzalez, C. & Pople, J. A. *Gaussian09, Revision E.01* (Gaussian, Inc./Wallingford CT, 2004).

(9) Becke, A. D. Density−functional thermochemistry. III. The role of exact exchange. *J. Chem. Phys.* **98**, 5648–5652 (1993).

(10) Perdew, J. P. *Unified Theory of Exchange and Correlation Beyond the Local Density*

Approximation/Electronic Structure of Solids (Eds: Ziesche, P. & Eschrig, H.) 11–20 (Akademie Verlag/Berlin, 1991).

(11) Dunning, T. H., Jr & Hay, P. J. *Methods of Electronic Structure Theory/Modern Theoretical Chemistry* (Ed: Schaefer, H. F., III), **3**, 1–27 (Springer, 1977).

(12) Hay, P. J. & Wadt, W. R. *Ab initio* effective core potentials for molecular calculations. Potentials for the transition metal atoms Sc to Hg. *J. Chem. Phys.* **82**, 270–283 (1985).

(13) Petersson, G. A., Bennett, A., Tensfeldt, T. G., Al-Laham, M. A., Shirley, W. A. & Mantzaris, J. A complete basis set model chemistry. I. The total energies of closed-shell atoms and hydrides of the first-row elements. *J. Chem. Phys.* **89**, 2193–2218 (1989).

(14) Petersson, G. A. & Al-Laham, M. A. A complete basis set model chemistry. II. Open-shell systems and the total energies of the first-row atoms. *J. Chem. Phys.* **94**, 6081–6090 (1991).

(15) Clark, T., Chandrasekhar, J., Spitznagel, G. W. & Schleyer, P. v. R. Efficient diffuse function-augmented basis sets for anion calculations. III. The 3-21+G basis set for first-row elements, Li–F. *J. Comp. Chem.* **4**, 294–301 (1983).

(16) Dunning, T. H., Jr & Hay, P. J. *Methods of Electronic Structure Theory/Modern Theoretical Chemistry* (Ed: Schaefer, H. F., III), **3**, 1–27 (Springer, 1977).

(17) Hay, P. J. & Wadt, W. R. *Ab initio* effective core potentials for molecular calculations. Potentials for the transition metal atoms Sc to Hg. *J. Chem. Phys.* **82**, 270–283 (1985).

(18) Grimme, S. Improved second-order Møller–Plesset perturbation theory by separate scaling of parallel- and antiparallel-spin pair correlation energies. *J. Chem. Phys.* **118**, 9095–9102 (2003).

(19) Fuentealba, P., Preuss, H., Stoll, H. & Von Szentpály, L. A proper account of core-polarization with pseudopotentials: single valence-electron alkali compounds. *Chem. Phys. Lett.* **89**, 418–422 (1982).
